# Phenotypic analysis of various *Clostridioides difficile* ribotypes reveals consistency among core processes

**DOI:** 10.1128/aem.00964-25

**Published:** 2025-06-24

**Authors:** Merilyn A. Beebe, Daniel Paredes-Sabja, Larry K. Kociolek, César Rodríguez, Joseph A. Sorg

**Affiliations:** 1 Department of Biology, Texas A&M University 168542, College Station, Texas, USA; 2 Division of Pediatric Infectious Diseases, Ann & Robert H. Lurie Children's Hospital of Chicago 2429, Chicago, Illinois, USA; 3 Facultad de Microbiología and Centro de Investigación en Enfermedades Tropicales, Universidad de Costa Rica 27915, San José, Costa Rica; University of Nebraska-Lincoln, Lincoln, Nebraska, USA

**Keywords:** physiology, ribotype, clade, phenotypes, *Clostridium difficile*

## Abstract

*Clostridioides difficile* infections (CDI) cause almost 300,000 hospitalizations per year, of which ~15%–30% are the result of recurring infections. The prevalence and persistence of CDI in hospital settings have resulted in an extensive collection of *C. difficile* clinical isolates and their classification, typically by ribotype. While much of the current literature focuses on one or two prominent epidemic ribotypes (e.g., RT027), recent years have seen several other ribotypes dominate the clinical landscape (e.g., RT106 and RT078). Some ribotypes are associated with severe disease and/or increased recurrence rates, but why certain ribotypes are more prominent or harmful than others remains unknown. Because *C. difficile* has a large, open pan-genome, this observed relationship between ribotype and clinical outcome could be a result of the genetic diversity of *C. difficile*. Thus, we hypothesize that the core biological processes of *C. difficile* are conserved across ribotypes/clades. We tested this hypothesis by observing the growth kinetics, sporulation, germination, production of toxin A and toxin B, bile acid sensitivity, bile salt hydrolase activity, and surface motility of 15 strains belonging to various ribotypes spanning each known *C. difficile* clade. In viewing these phenotypes across each strain, we see that core phenotypes (growth, germination, sporulation, and resistance to bile salt toxicity) are remarkably consistent across clades/ribotypes. This suggests that variations observed in the clinical setting may be due to unidentified factors in the accessory genome or due to unknown host factors.

**IMPORTANCE:**

*Clostridioides difficile* infections impact thousands of individuals every year, many of whom experience recurring infections. Clinical studies have reported an unexplained correlation between some clades/ribotypes of *C. difficile* and disease severity/recurrence. Here, we demonstrate that *C. difficile* strains across major clades/ribotypes are consistent in their core phenotypes. This suggests that such phenotypes are not responsible for variations in disease severity/recurrence and are ideal targets for the development of therapeutics meant to treat *C. difficile*-related infections.

## INTRODUCTION

*Clostridioides difficile* is a gram-positive, anaerobic, endospore-forming pathogen with two major life stages: the metabolically active vegetative cell and the dormant spore (1, 2). The spore is the transmissible form and provides extreme resistance to antibiotics, environmental stresses, and disinfection techniques (2–4). When a patient experiencing antibiotic-induced dysbiosis ingests *C. difficile* spores, the spores traverse the gut and germinate into the vegetative form (5–7). In the absence of a healthy microbiome, vegetative cells more efficiently colonize the host and can produce toxins that induce the symptoms characteristic of *C. difficile* infection (CDI) (diarrhea, pseudomembranous colitis, etc.) (8). These vegetative cells will form new spores which can remain in the gut or pass into the environment contributing to the spread/recurrence of disease (6). Of approximately 300,000 CDI-related hospitalizations in the US, about 15%–30% of patients experienced disease recurrence; the chance of recurrence increases with each subsequent infection (9, 10).

The primary virulence factors in CDI are two large exotoxins: TcdA (toxin A) and TcdB (toxin B) (8). These toxins are encoded within a 19.6 kb pathogenicity locus (*PaLoc*) that includes a positive (*tcdR*) and negative (*tcdC*) regulator of expression and a holin-like protein (*tcdE*) (11). Diagnosis of CDI is typically confirmed by testing fecal samples of patients presenting with CDI symptoms for the presence of one or both C. *difficile* toxins (12). The presence of toxins is known to be variable between species and is often used to characterize *C. difficile* strains (*e.g*., toxinotyping) (13).

In 2006, the first fully assembled *C. difficile* genome was published (14). Currently, there are greater than 19,000 *C*. *difficile* genomes deposited in the NCBI database and greater than 31,000 deposited in Enterobase (15, 16). Broadly, *C. difficile* genomes are ~4 Mbp in size and contain ~4 k genes (14, 17–19). About 25%–50% of these genes belong to the core genome (genes shared among sequenced strains), while the pan-genome (all genes both shared and unique found in sequenced strains) is generally agreed to consist of ~6 k genes with no currently defined limit (20–22). In addition, greater than 11% of any given *C. difficile* genome consists of mobile elements (transposons, prophage, a prophage-like *sigK* intervening [skin] element, etc.) (14). Phylogenetic analyses group *C. difficile* strains into five main clades and three cryptic clades (23, 24). Most studies agree that clades 1 and 2 are closely related, while clade 5 exhibits the most genetic distinction, with recent studies suggesting that clade 5 is undergoing speciation (22, 25, 26). The relatively small size of the core genome, open pan-genome, large number of mobile elements, and observed evolutionary distance all indicate that *C. difficile*, as a species, has substantial genetic variation between its members.

In the clinical setting, *C. difficile* strains are typically classified using various typing methods (ribotyping, restriction endonuclease analysis, multilocus sequence typing [MLST], toxinotyping, serotyping, etc.) (13, 27–31). Of these, ribotyping remains the most popular and clinically relevant. Historically, ribotype 027 (RT027) strains have been associated with the worst CDI outbreaks and thus are generally the most well studied (18, 19, 32, 33). However, the prominent strains isolated from more recent outbreaks belong to less well-studied ribotypes (e.g., RT106 or RT078) (34–42). Because *C. difficile* is so genetically diverse, ribotype-specific clinical outcomes may be attributable to processes encoded by the accessory genome and not to functions encoded in the core genome. Thus, we hypothesize that processes central to *C. difficile* biology (e.g., vegetative growth, sporulation, germination, and resistance to bile salt toxicity) are conserved across ribotype/clade. To test this hypothesis, we collected strains from ribotypes belonging to each of the five classical clades. We then measured their growth in rich and minimal media, spore production, response to germinants, production of toxin A and toxin B, bile salt hydrolase activity (BSHA), resistance to bile acid toxicity, and surface motility. Our analyses indicate that, while the strains show variations in their ability to process taurocholate (TA)-conjugated bile salts and in their ability to metabolize various carbohydrates, they exhibit remarkably consistent core phenotypes with no strong patterns across ribotypes/clades. These results support the hypothesis that despite the evolutionary variation observed between *C. difficile* strains, certain processes that are central to *C. difficile* biology remain consistent across ribotype/clade.

## MATERIALS AND METHODS

### Bacterial strains and growth conditions

*C. difficile* R20291 was used as an experimental control for all assays performed in this study. *C. difficile* strains LC5624, LK3P-030, and LK3P-081 (all identified as ST-42 by *in silico* MLST and presumed to be RT106) were obtained from pediatric patients receiving care at the Ann and Rober H. Lurie Children’s Hospital of Chicago, Chicago, IL, USA. *C. difficile* strains M68 and M120 were obtained from the collection of Dr. Trevor Lawley (Sanger Institute, Hinxton, UK). The remaining strains were collected by Dr. Daniel Parades-Sabja (Texas A&M University, College Station, TX, USA) and César Rodríguez (Facultad de Microbiología and Centro de Investigación en Enfermedades Tropicales, Universidad de Costa Rica, San José, Costa Rica) from various outbreaks in Central/South America.

Strains were routinely grown anaerobically (Coy Laboratories, model B, atmospheric H_2_ was kept between 3% and 4% using 10% H_2_, 5% CO_2_, and 85% N_2_ gas mix) at 37°C. Each strain was grown on/in either brain heart infusion medium (Becton, Dickinson and Company, Franklin Lakes, NJ, USA) supplemented with yeast extract and 0.1% (wt/vol) L-cysteine (BHIS) and, where indicated, with 11 mM theophylline, 10 µg/mL thiamphenicol, 50 µg/mL kanamycin, and/or 250 µg/mL D-cycloserine. For some growth assays, strains were grown in *C. difficile* minimal medium (CDMM; 1% [wt/vol] casamino acids, 2 mM L-tryptophan, 4 mM L-cysteine, 35.2 mM Na_2_HPO_4_·7H_2_O, 60 mM NaHCO_3_, 7 mM KH_2_PO_4_, 20 mM NaCl, 45 mM glucose [or indicated carbohydrate], 400 µM [NH_4_]_2_SO_4_, 177 µM CaCl_2_·2H_2_O, 96 µM MgCl_2_·6H_2_O, 50.6 µM MnCl_2_·4H_2_O, 4.2 µM CoCl_2_·6H_2_O, 34 µM FeSO_4_·7H_2_O, 4 µM D-biotin, 2 µM calcium-D-pantothenate, and 6 µM pyridoxine). Where indicated, the pH of BHIS was adjusted using acetic acid/sodium hydroxide or supplemented with 0.1% TA (GoldBio S-121-100) in a solid medium and 1 mM TA, taurodeoxycholic acid (TDCA; Millipore Sigma 580221-50GM), hyodeoxycholic acid (HDCA; MP Biomedicals 157514), cholic acid (CA; Sigma Aldrich C1129-100G), chenodeoxycholic acid (CDCA; ACROS Organics RK-88245-39), or deoxycholic acid (DCA; Sigma Aldrich D2510-100G) for liquid cultures. Except for TA, which is soluble in water, bile acids stocks were made in dimethylsulfoxide (DMSO).

### Plasmid and strain construction

Theophylline-mediated allelic exchange plasmids were constructed as described previously (43). Briefly, 500 bp regions up and downstream of the indicated genes were amplified via PCR using primers 3,814–3,817 for pMAB50 and primers 4,133–4,136 for pMAB55. The PCR products were inserted via Gibson assembly into pJB94 between the *Not*I and *Xho*I restriction sites. The resulting plasmids were transformed into *Escherichia coli* DH5α.

The allelic exchange plasmids were then transformed into *E. coli* HB101 for conjugation into *C. difficile*. For each conjugation, 1 mL of the log-phase culture of *E. coli* HB101 harboring the appropriate plasmid was pelleted and mixed with 500 µL of a log-phase *C. difficile* culture. The mixture was spotted onto a pre-reduced BHIS plate supplemented with theophylline (Theo) and incubated overnight. The subsequent growth was suspended in 500 µL BHIS and spread across five pre-reduced, BHIS Theo plates supplemented with thiamphenicol, kanamycin, and D-cycloserine. Growth was monitored for 4 days, and colonies that appeared were tested for the presence of the plasmid using primers 173 and 174 for *catP*. Following confirmation of a successful conjugation, plasmid integration was selected for by passaging on BHIS supplemented with thiamphenicol and confirmed via PCR using primers 2,993 and 3,819 for pMAB50 and primers 2,980 and 3,819 for pMAB55. Colonies that harbored integrated plasmids were then plated on BHIS Theo to promote the excision of the plasmid from the genome. Mutations were screened via PCR using primers 2,980 and 2,981 for the *C. difficile* Δ*sigG* strain, which was then cured of the allelic exchange plasmid via repeated passages on BHIS (no antibiotic) medium. Using the same method, a *tcdR* deletion was introduced (and confirmed using primers 2,992 and 2,993) to generate the *C. difficile* Δ*sigG*Δ*tcdR* strain.

### Whole-genome sequencing

Genomic DNA was extracted for all strains except *C. difficile* R20291 (GenBank accession number NZ_CP029423.1), *C. difficile* LC5624 (GenBank accession number CP022524.1), *C. difficile* M68 (GenBank accession number NC_017175.1), and *C. difficile* M120 (GenBank accession number NC_017174.1) for which genomes are already published. Briefly, each strain was grown for 18 hours in a BHIS medium. The cells were pelleted, lysed, and subjected to phenol-chloroform extraction. This genomic DNA was sent to SeqCoast Genomics (Portsmouth, NH, USA) for long-read Oxford Nanopore sequencing. The long-read data were assembled using the Flye plugin for *de novo* assembly in Geneious Prime (version 2024.0.5) (44, 45). To improve genome quality, Illumina sequencing reads for each strain were mapped to the assembled genomes using BBMapper (46). The genome alignments were performed using the MAUVE plugin (47). Phylogenies were generated using the Geneious Tree Builder with a Tamura-Nei genetic distance model and the neighbor-joining method for three individual locally collinear blocks (LCBs) produced by the MAUVE alignment.

### Core genome analysis

The core genome (defined here as the genes present in all strains under study) was determined using the finalized genome for all strains except *C. difficile* R20291 and Spine (version 0.3.1) (48). For each strain, the resulting files were concatenated in Geneious Prime and annotated against *C. difficile* CD630 (GenBank accession number NC_009089) in keeping with the annotation of the *C. difficile* core genome multilocus sequence typing scheme (20). The results of this analysis can be found in Table S1.

### Protein alignment

The genes encoding each of the indicated proteins were extracted from the assembled and MAUVE-aligned genomes. Their sequences were translated and aligned using Clustal Omega in Geneious Prime (49). The amino acids were shaded according to similarity (Pam140 Score Matrix) with white indicating 100% amino acid identity across all observed strains and black indicating <60% identity.

### Growth curves and doubling times

For growth curves in a BHIS medium, strains were grown in 5 mL pre-reduced BHIS liquid medium for 12 hours. Cultures were back diluted to an OD_600_ = 0.05 in 5 mL and allowed to grow to an OD_600_ = 0.5. This log-phase culture was then used to inoculate the experimental culture (40 mL) to an OD_600_ of 0.05 at *T*_0_. OD_600_ measurements were taken every 30 minutes for 8 hours using a Biowave Cell Density Meter CO8000. When the OD_600_ reached ≥0.3, the OD measurements across 2 hours of growth were used to calculate doubling time.

For growth curves in CDMM, strains were grown in 5 mL pre-reduced CDMM (made with a final concentration of either 45 mM glucose/fructose, 53 mM xylose, or 50 mM trehalose, where indicated) for 24 hours. Cultures were back diluted in 5 mL to an OD_600_ = 0.05 and grown for 12 hours to an OD_600_ ≈ 0.5–0.7. The culture was then used to inoculate 3 mL of the indicated medium to a starting OD_600_ of 0.05 in a 12-well plate. The plate was then placed in a Stratus Microplate Reader (Cerillo, Charlottesville, VA, USA) (located in the anaerobic chamber) where OD_600_ measurements were collected every 3 minutes for 22 hours. Each time point represents the average reading from three different sensors measuring a single well. Due to random blockage of sensors during the assay, the data were filtered using the *z*-score method. Doubling times were calculated using the most linear portion of the OD curves and the formula *t*_2_ = ln(2)/(*k*), where *k* is the growth rate determined using the formula *k* = LN(OD_t2_/OD_t1_)/(t2 − t1).

### Spore purification

Spores were purified as previously described (50–57). Briefly, the indicated strains were plated onto 10 pre-reduced BHIS plates and incubated for 5 days. The growth from each plate was scraped into 1 mL 18 MΩ dH_2_O and stored at 4°C. After a minimum period of 24 hours, cells were resuspended in 1 mL 18 MΩ dH_2_O and centrifuged for 1 minute at 14,000 × *g*. The supernatant was removed, and subsequent washes with 18 MΩ dH_2_O were performed to separate the spores from vegetative cells/cell debris in distinct layers that were gradually removed, and the cell pellets were combined with each wash. Final contaminants were removed by placing the cells on 9 mL of 50% sucrose and centrifuging for 20 minutes at 4,000 × *g* and 4°C. The supernatant was discarded, and the pellet (containing the purified spores) was washed thrice with 18 MΩ dH_2_O to remove the remaining sucrose. The final pellet was resuspended in 1 mL 18 MΩ dH_2_O and stored at 4°C.

### Sporulation

The sporulation assay was performed as previously described, with some slight modifications (3, 58). A 16-hour culture of the indicated strain was back diluted in 5 mL to an OD_600_ = 0.05 and allowed to grow to an OD_600_ = 0.5. One hundred microliter of the log-phase culture was plated on pre-reduced BHIS agar medium and incubated for 48 hours. One-quarter of the plate was scraped into 1 mL of 1× phosphate-buffered saline (PBS, pH 7.4), and 500 µL of this suspension was treated with 100% ethanol for 20 minutes. The treated cells were serially diluted in 1× PBS with 0.1% TA and plated on pre-reduced BHIS TA plates. The plates were incubated for 48 hours prior to CFU enumeration.

### Germination assay

Germination was assessed using an OD-based assay as described previously (51, 52). Briefly, samples of purified spores were adjusted to an OD_600_ of 0.5. The spores were heat treated at 65°C for 30 minutes. For each of the tested germinant concentrations, 5 µL of the spore sample was suspended in 95 µL of the appropriate germination buffer. For all samples, the buffer contained 0.5 M HEPES and 50 mM NaCl, pH 7.2. For co-germinant (glycine) efficiency, the buffer contained 10 mM TA and various amounts of glycine (final concentrations of 0 mM, 0.1 mM, 0.2 mM, 0.4 mM, 0.6 mM, 0.8 mM, 1 mM, 2 mM, or 5 mM). For bile-acid germinant (TA) efficiency, the germination buffer contained 100 mM glycine, 10% (vol/vol) DMSO (to control for the DMSO used to dissolve CDCA), and various amounts of TA (final concentrations of 0 mM, 0.1 mM, 0.2 mM, 0.5 mM, 1 mM, 2 mM, 5 mM, or 10 mM). For CDCA, the germination buffer was supplemented with 100 mM glycine and 1 mM CDCA and TA to a final concentration of 1 mM, 2 mM, 5 mM, 10 mM, 15 mM, 20 mM, 30 mM, or 50 mM. The OD_600_ of each sample was recorded every 30 seconds over the course of 1 hour using a SpectraMax M3 spectrophotometer (Molecular Devices, San Jose, CA, USA). The plate was shaken vigorously 5 seconds before each measurement.

A germination curve was generated for each strain/germinant combination by plotting the OD_600_ at a given time (*T*_*x*_) divided by the OD_600_ at time zero (*T*_0_) vs time (the controls for all strains/germinants and an example for R20291 with varying concentration of glycine are shown in Fig. S1) (51, 52, 59–62). Germinant sensitivity was calculated using the maximum slope for each germination curve. The slope was plotted against (co)-germinant concentration to generate a Michaelis-Menten graph. A Lineweaver-Burke plot was generated, and from this, the Ki/EC_50_ was calculated. As this is a multi-enzyme/protein process, this does not provide a bona fide *K*_*m*_. Here, EC_50_ is defined as the concentration of germinant that produces half the maximum germination rate. This value is used here and in many prior studies to provide a measurement for spore interactions with germinants (51, 52, 59–65). The efficiency of the competitive inhibitor was calculated as previously described using the following equation *K*_*i*_ = (inhibitor)/([K_CDCA_/K_TA_] − 1) (59–62).

### ELISAs

Each strain (including *C. difficile* R20291 and the *C. difficile* R20291 Δ*sigG*Δ*tcdR* negative control) was grown in 5 mL pre-reduced BHIS broth for ~4 hours until log-phase growth was reached. Each culture was then back diluted in 5 mL BHIS to an OD_600_ = 0.05 and incubated for 48 hours. At 24 and 48 hours, 250 µL samples of culture were removed from the anaerobic chamber and stored at −80°C. Samples were thawed at room temperature and combined with 1 mL of the 1× fecal extraction buffer from the Fecal *C. difficile* Toxin A (KT-834) or Toxin B (KT-835) enzyme-linked immunosorbent assay (ELISA) kit (Epitope Diagnostics San Diego, CA, USA). The samples were centrifuged for 5 minutes at 900 × *g* and 100 µL of the clarified sample was added to a toxin A or toxin B antibody-coated microplate in duplicate. The plate was incubated for 1 hour, static, at room temperature. Each well was washed five times with 350 µL of 1× ELISA wash solution prior to treatment with 100 µL of the tracer antibody. The plate was then incubated for 30 minutes, static, at room temperature. The wash was repeated five more times, and 100 µL of the ELISA HRP substrate was added to each well. The plate was incubated for 20 minutes, static, at room temperature prior to the addition of 100 µL of ELISA stop solution. The absorbance of each well was read at 450 nm using a SpectraMax M3 spectrophotometer. The average absorbance value of the technical triplicates for each strain was normalized to the absorbance value of the *C. difficile* R20291 Δ*sigG*Δ*tcdR* negative control strain.

### Bile salt sensitivity

Each strain was grown in 5 mL pre-reduced BHIS broth for 16 hours, back diluted in 5 mL to an OD_600_ = 0.05, and allowed to grow to an OD_600_ = 0.5. Fifty microliter of these cultures was added to pre-reduced BHIS broth of the indicated pH and bile acid concentration (obtained through a series of 1:1 dilutions to a final volume of 500 µL). The samples were incubated for ~18 hours, and MICs were assessed by the presence/absence of growth as indicated by a visual inspection of culture turbidity.

### Bile salt hydrolase activity

Bile salt hydrolase activity assays were performed as described previously (54). Briefly, strains were grown in 5 mL BHIS liquid medium for 16 hours. A total of 10^8^ CFU from these cultures were transferred into 5 mL BHIS supplemented with 1 mM TA or TDCA and incubated for 24 hours after which the cultures were centrifuged for 10 min at 4,000 × *g*. The pellet was suspended in 100% methanol. One millimolar HDCA (an internal standard) was added to the supernatant before being lyophilized and suspended in methanol. The suspended pellet and dried supernatant were combined. Each strain was run alongside three *C. difficile* R20291 controls: a negative control without TA, a control with 1 mM each TA/TDCA, CA/DCA, and HDCA all added to the spent supernatant, and a positive control with 1 mM TA added before the 24-hour incubation (Fig. S2).

The bile salts found in each sample were separated by reverse-phase high-performance liquid chromatography using a Shimadzu Prominence system (Shimadzu, Kyoto, Japan) (54, 66–68). For each strain, 30 µL of the sample was separated on a Synchronis C18 column (4.6 by 250 mm, 5 µm particle size, ThermoFisher, Waltham, MA, USA) using a methanol-based mobile phase (53% methanol, 24% acetonitrile, and 30 mM ammonium acetate [pH 5.6]). A Sedere Sedex model 80 low temperature-evaporative light scattering detector using 50 psi Zero Grade air at 94°C detected the bile salt peaks. Percent deconjugation was calculated using the area under the peak for CA/DCA divided by the sum of the areas under the peaks for TA/TDCA and CA/DCA.

### Surface motility

Surface motility assays were performed as previously described (69). Briefly, strains were grown in 5 mL BHIS for 16 hours, diluted 1:50 in 5 mL fresh BHIS medium, and grown until the OD_600_ reached ~0.5. From these cultures, 10 µL was spotted onto pre-reduced BHIS 1.5% agar medium and incubated for 5 days. Plates were then imaged using a Bio-Rad GelDoc XR+ (Bio-Rad Laboratories, Hercules, CA, USA), and the growth diameters were quantified and normalized to *C. difficile* R20291.

### Statistical analysis

All data represent the average from three independent biological replicates with the error bars indicating the SEM. For all assays except toxin production, statistical significance was determined using the one-way analysis of variance (ANOVA) analysis function from GraphPad Prism (version 9.0.2 for Windows, GraphPad Software, San Diego, California USA). When results were compared to *C. difficile* R20291, Šidák’s multiple comparisons test was used, while Tukey’s multiple comparisons test was used when comparing between ribotypes/clades. For the toxin A or B ELISA results, a two-way repeated measures ANOVA analysis was performed using the analysis function in GraphPad Prism. Asterisks indicate *P*-values with, * ≤0.05, ** ≤0.02, *** ≤0.01, and **** ≤0.0001.

## RESULTS

### Strain collection and genomic analysis

**Fig 1:**
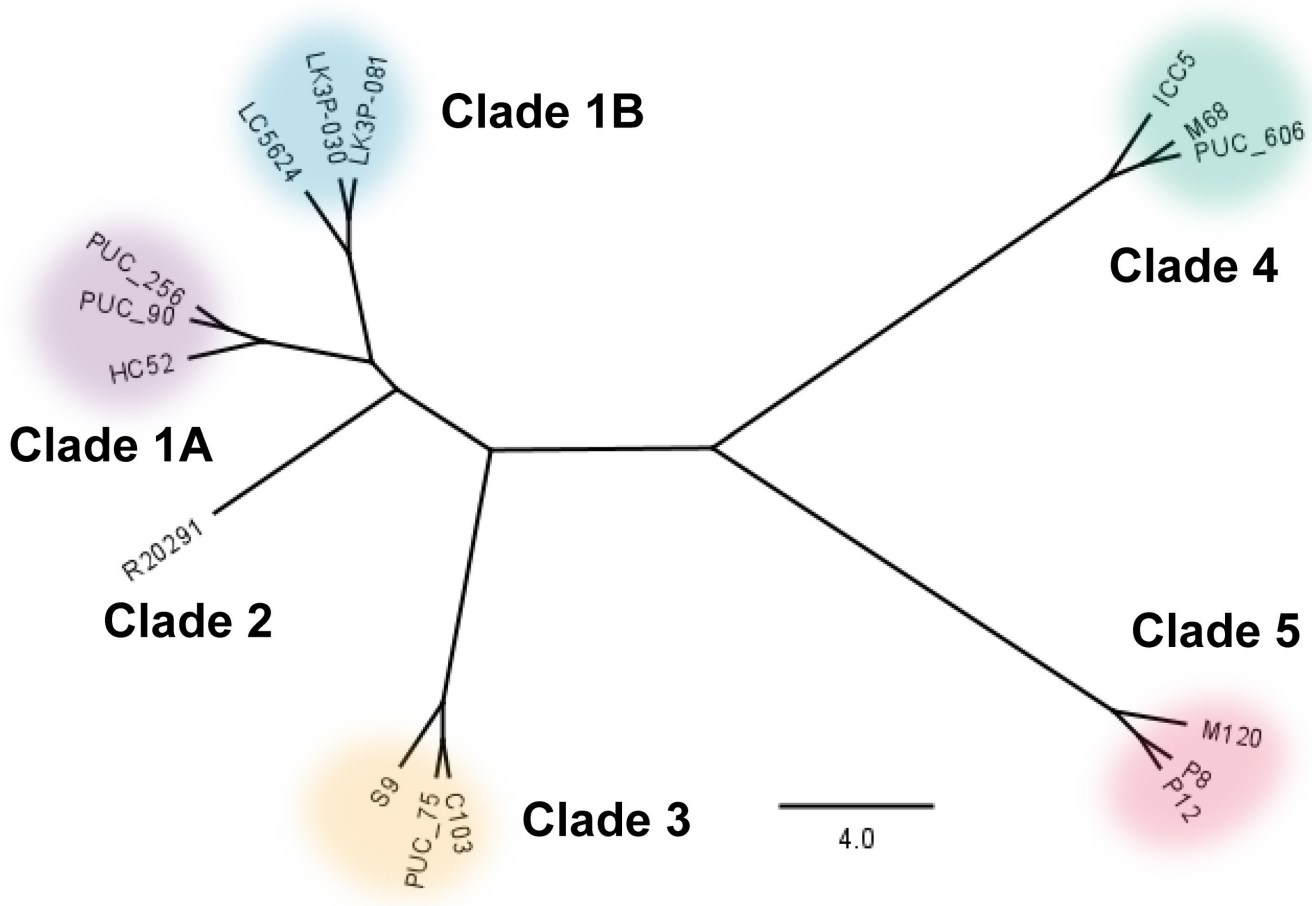
Phylogeny of strains used in this study. The neighbor-joining phylogeny generated for the strains in this study derived from LCB 60, a 764,748 bp segment of the MAUVE alignment representing approximately 25% of any given genome in the study. The phylogeny was created using the Geneious Tree Builder application in the Geneious Prime software using the Tamura-Nei genetic distance model. Strains are grouped by their respective ribotypes/clades, with the scale bar representing the number of substitutions per 1,000 bp.

To study phenotypic variation among the *C. difficile* clades, we collected 15 different clinical isolates of *C. difficile* (Table 1). *C. difficile* R20291 is a well-characterized member of clade 2 and was used as a control throughout (3, 43, 51–57, 66, 70–80). Each of the five main clades is represented in this study by strains belonging to six prominent ribotypes. The indicated ribotype was selected based on its clinical relevance (17, 34, 41, 81–83). Apart from those strains whose genomes have already been published (*C. difficile* R20291, *C. difficile* LC5624, *C. difficile* M68, and *C. difficile* M120), we assembled the genomes for each of the strains and deposited them in the NCBI database. Phylogenetic analysis clustered the strains into the five classical clades, consistent with previously published data (Fig. 1; Fig. S3) (22, 25, 26).

**TABLE 1:** Strains used in this study

Strain	Clade	Ribotype
R20291	2	027
PUC_256	1A	014-020
HC52	1A	014-020
PUC_90	1A	014-020
LC5624	1B	106
LK3P-030	1B	106
LK3P-081	1B	106
PUC_75	3	023
S9	3	023
C103	3	023
M68	4	017
PUC_606	4	017
ICC5	4	017
M120	5	078
P8_S146	5	078
P12_S145	5	078

### *C. difficile* growth is consistent in rich medium

**Fig 2:**
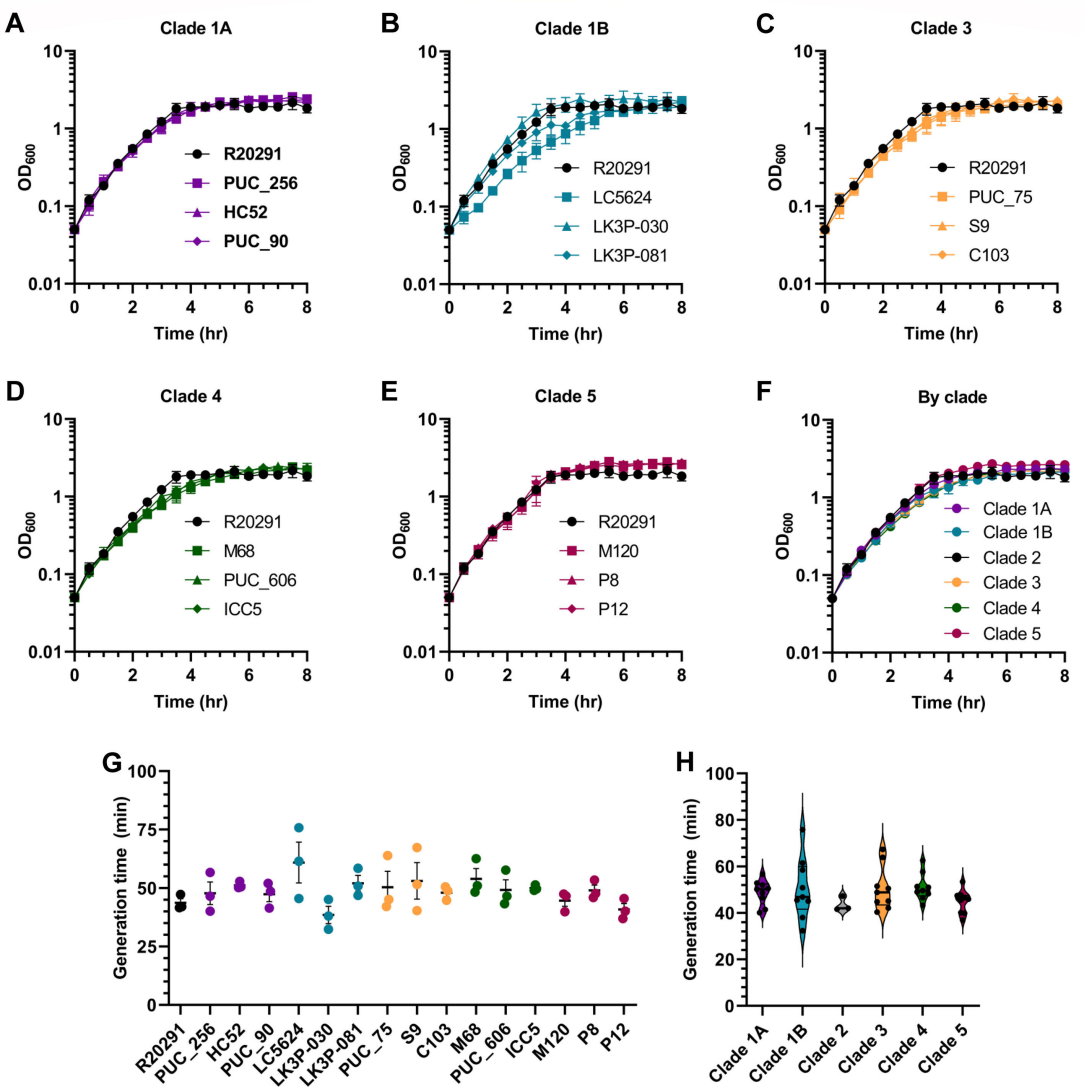
Growth of strains in rich medium. OD_600_ measurements for clade 1A (**A**), clade 1B (**B**), clade 3 (**C**), clade 4 (**D**), and clade 5 (**E**) strains were taken every 30 minutes over the course of 8 hours. These same data are shown in panel **F** grouped by ribotype/clade. Data from the most linear portion of the growth curve were used to calculate the doubling time presented by strain (**G**) and by ribotype/clade (**H**). Data points represent the average from independent biological triplicates with error bars representing the SEM. For panel **G**, Šidák’s multiple comparisons test (comparing all strains to *C. difficile* R20291) was used, while Tukey’s multiple comparisons test (comparing all clades to each other) was used for panel **H**. No statistically significant differences between strains were found.

In the laboratory setting, *C. difficile* is often cultured in a rich medium (BHIS). Because this was the medium in which all our assays would be performed, we first sought to determine if there were any inherent growth differences in this medium. Growth curves in a BHIS medium were obtained for each strain and indicated little variation in overall growth kinetics. We observed minimal differences between each strain in a given clade (Fig. 2A through E). All strains reached the stationary phase within 4 hours of growth and a maximum OD_600_ of 2.0–3.0. Moreover, there were no significant differences in growth when the data were grouped by clade (Fig. 2F). For a more objective comparison, we determined the generation times for each strain. The generation times for individual strains, including *C. difficile* R20291 (clade 2), were calculated at 40–60 minutes (Fig. 2G). The average generation time for each clade was ~50 minutes (Fig. 2H). Taken together, this indicates that none of the tested strains exhibited a notable change in growth rate in the BHIS medium relative to each other.

### Clade 4 strains are limited in their ability to use various carbohydrates

**Fig 3:**
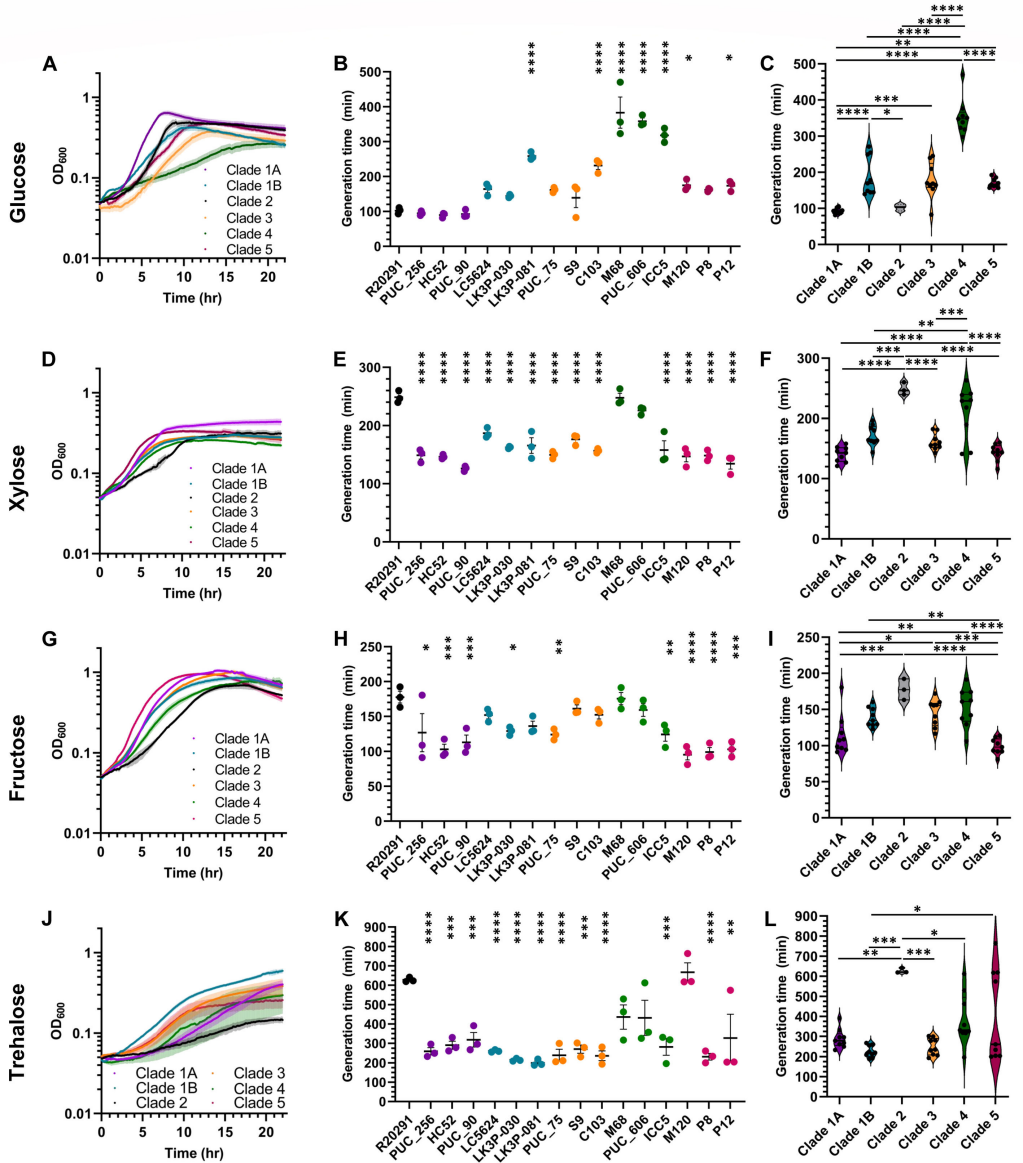
Growth of strains in minimal medium. Strains were grown in CDMM supplemented with either glucose (**A**), xylose (**D**), fructose (**G**), or trehalose (**J**). OD_600_ measurements for each strain were taken every 3 minutes for 22 hours. Data from the most linear portion of the growth curve were used to calculate generation times for growth in CDMM supplemented with glucose (**B and C**), xylose (**E and F**), fructose (**H and I**), or trehalose (**K and L**). Data points represent the average from independent biological triplicates with error bars representing the SEM. For panels B, E, H, and K, Šidák’s multiple comparisons test (comparing all strains to *C. difficile* R20291) was used, while Tukey’s multiple comparisons test (comparing all clades to each other) was used for panels C, F, I, and L. Asterisks indicate *P*-values of * ≤0.05, ** ≤0.02, *** ≤0.01, and **** ≤0.0001.

Given that BHIS is a rich medium, the lack of variation in growth between strains was not entirely surprising. To determine if the strains responded differently in minimal medium, we cultured them in standard CDMM (containing glucose). Under these conditions, all strains exhibited less growth compared to growth in BHIS medium as indicated by noticeably increased generation times (Fig. 3A through C). While the *C. difficile* R20291 strain (clade 2) had a generation time of 100 minutes, *C. difficile* strains LK3P-081 (clade 1B), C103 (clade 3), M68 (clade 4), PUC_606 (clade 4), ICC5 (clade 4), M120 (clade 5), and P12 (clade 5) had slower generation times. The clade 4 strain, *C. difficile* M68, had the slowest generation time at ~400 minutes (Fig. 3B). When these data are grouped by clade, the clade 1A strains had a generation time of ~90 minutes, while the clade 4 strains had a generation time of ~350 minutes (Fig. 3C). Taken together, the data indicate that clade 4 strains tested demonstrate lower growth rates in standard CDMM relative to the other strains.

Because *C. difficile* can incorporate different carbohydrates into its metabolic pathways, we sought to determine if any of the strains favored one carbohydrate source over another. Previous research has identified xylose as an important carbon source for many bacteria (84, 85). Xylose forms a five-carbon, six-member ring like glucose, but it lacks a 6’ carbon and associated hydroxyl group. When testing growth in CDMM supplemented with xylose (CDMM-xyl), we observed slight variations between strains. All strains reached the stationary phase between 6 and 10 hours (Fig. 3D). Contrary to what was observed for growth in standard CDMM, generation times for all strains except *C. difficile* R20291 (246 minutes), *C. difficile* M68 (240 minutes), and *C. difficile* PUC_606 (220 minutes) were below 200 minutes (Fig. 3E). Additionally, while most of the strains grew better than the control in CDMM-xyl, clade 4 strains, once again, appeared to have a decreased growth rate in this medium in comparison to the other clades with an average doubling time of ~210 minutes (Fig. 3F).

Fructose, another common carbohydrate that is important for *C. difficile* metabolism, is the primary carbohydrate found in a medium that allows for selection/isolation of *C. difficile* in clinical environments (TCCFA) (86, 87). Like glucose, it contains six carbons but forms a five-member ring. When grown in CDMM supplemented with fructose, the strains reached a stationary phase between 8 and 12 hours (Fig. 3G). *C. difficile* strains LK3P-030 (clade 1B), PUC_75 (clade 3), and ICC5 (clade 4), as well as all clade 1A and five strains, exhibited generation times of 100–130 minutes. These times were lower than the *C. difficile* R20291 strain (clade 2), which had a 177-minute generation time (Fig. 3H). *C. difficile* strains LC5624 (clade 1B), LK3P-081 (clade 1B), S9 (clade 3), C103 (clade 3), M68 (clade 4), and PUC_606 (clade 4) all grew similarly compared to *C. difficile* R20291 with generation times between 130 and 175 minutes. When grouped by clade, strains from clades 1B, 3, and 4 showed a ~50-minute slower generation time relative to the remaining strains (Fig. 3I).

Finally, we tested the growth of the strains in CDMM supplemented with 50 mM trehalose (CDMM-tre), a concentration previously shown to support the growth of strains spanning multiple ribotypes (25). Trehalose is composed of two α,α−1,1-linked glucose molecules and has, arguably, been associated with an increase in CDI (25, 88, 89). Growth in CDMM-tre was variable, with clade 1B strains reaching the stationary phase around 10 hours of growth and clade 1A strains reaching the stationary phase around 20 hours of growth (Fig. 3J). All strains, except for *C. difficile* M120 (660 minutes), had a lower generation time than the *C. difficile* R20291 strain (clade 2, 620 minutes; Fig. 3K). Additionally, generation times for *C. difficile* strains PUC_256 (clade 1A), HC52 (clade 1A), PUC_90 (clade 1A), LC5624 (clade 1B), LK3P-030 (clade 1B), LK3P-081 (clade 1B), PUC_75 (clade 3), S9 (clade 3), C103 (clade 3), ICC5 (clade 4), P8 (clade 5), and P12 (clade 5) were similar to the generation times observed in CDMM-xyl at ~200–300 minutes. Overall, clade 4 strains had consistently slower growth in CDMM-tre relative to the other clades (Fig. 3L).

### Variations in carbohydrate metabolism protein sequences are not consistent with phenotypic differences

The catabolite control protein, CcpA, is encoded by all strains in this study (Fig. S5). Because there was little variation in the CcpA protein sequence, we hypothesize that there should be no major change in the global regulation of carbohydrate metabolism. In all tested strains, the xylose utilization operon was also present. Some variants appeared in the XylA (isomerase), XylB (kinase), and XylR (transcriptional regulator) protein sequences (Fig. S6 to S8). However, none of these variations were consistent between the clades 1A, 1B, 3, and 5 strains, which had faster generation times compared to the *C. difficile* R20291 strain (Fig. 3D and F).

Previous reports found heterogeneity in the genes responsible for processing trehalose and corresponding differences in the ability of strains from different ribotypes to grow in media in which trehalose was the sole carbon source (25, 89). We observed the same for the strains tested here, specifically when comparing the phosphotrehalase (TreA) protein sequence (Fig. S9). The clade 3 *C*. *difficile* strains PUC_75 and S9 had nonsense mutations in TreA, and *C. difficile* strains C103 (clade 3), M120 (clade 5), P8 (clade 5), and P12 (clade 5) were missing TreA at this locus entirely. The trehalose operon repressor (TreR) also showed some variations and was missing entirely at the canonical locus in the clades 3 and 5 strains (Fig. S10). Both the *treR* and *treA* genes were found in an operon elsewhere in the genomes for the clade 5 strains, and these copies are included in the alignments (Fig. S9 and S10). Clade 3 strain, *C. difficile* C103, contained an intact copy of *treA* at an additional locus but did not possess a corresponding copy of *treR*, suggesting that *treA* may be regulated differently in this strain. The remaining clade 3 strains (*C. difficile* PUC_75 and S9) do not possess an intact copy of *treA*, suggesting the existence of another, non-canonical, trehalose metabolism pathway in these strains.

### Spore production differences are minor between strains

**Fig 4:**
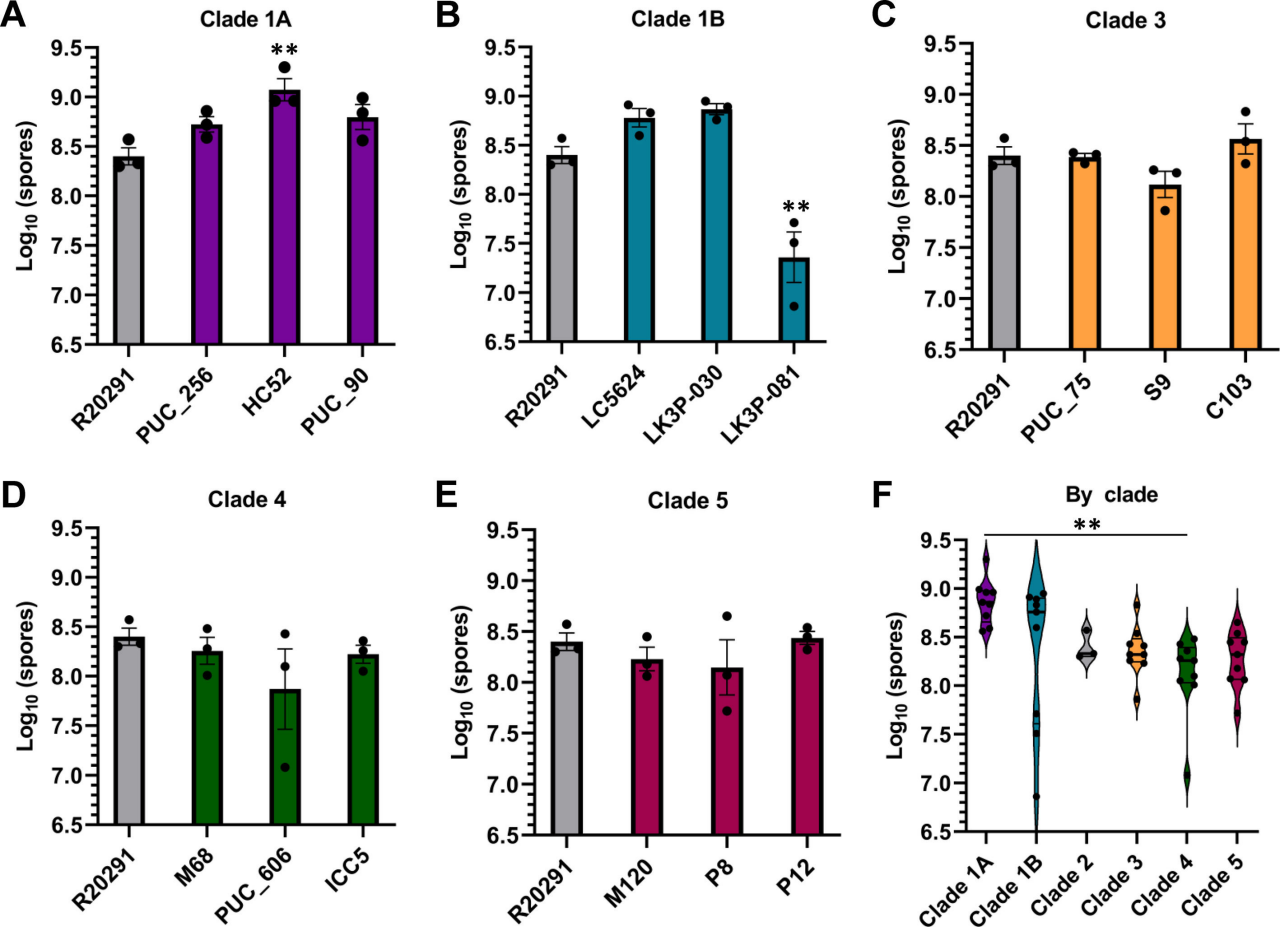
Spore production by strains over 48 hours. The number of spores produced on BHIS over 48 hours for clade 1A (**A**), clade 1B (**B**), clade 3 (**C**), clade 4 (**D**), and clade 5 (**E**) was reported on a log_10_ scale. These same data are grouped by clade in **F**. Data points represent the average from independent biological triplicates with error bars representing the SEM. For A–E, Šidák’s multiple comparisons test (comparing all strains to *C. difficile* R20291) was used, while Tukey’s multiple comparisons test (comparing all clades to each other) was used for **F**. Asterisks indicate a *P*-value of ** ≤0.02.

Given that *C. difficile* is transmitted by spores, changes in sporulation levels could explain why some strains are more prevalent in the clinical setting than others; an increase in spore number may lead to increased disease spread/recurrence. We measured sporulation in each of the strains over a 48-hour period (Fig. 4A through E) and observed a small increase in spore number for the clade 1A strain *C. difficile* HC52 (clade 1A, ~10^9^ spores) and a decrease for the clade 1B strain *C. difficile* LK3P-030 (~10^7^ spores), relative to the *C. difficile* R20291 strain (clade 2, ~10^8^ spores). When these data were grouped by clade, the clade 1A strains produced ~10-fold more spores on average than the clade 4 strains (Fig. 4F).

### *C. difficile* clinical isolates are more sensitive to germinants than the *C. difficile* R20291 lab strain

Bile salts are cholesterol derivatives found in mammalian digestive systems and contribute to the absorption of nutrients (90). These compounds are produced in the liver and circulate through the small intestine before being recycled back to the liver. A small portion of these bile acids escape enterohepatic recirculation and enter the colon (91, 92). Germination by *C. difficile* spores occurs in response to two signals: a bile acid germinant and an amino acid co-germinant (93). Prior work has shown that TA and glycine are the most efficient germinants *in vitro*, and consequently, they are used frequently in germination analyses (51). Because germination is required for the successful outgrowth of vegetative cells from the dormant spore form, we sought to determine how the strains responded to germinants. Previous work from our lab and others has demonstrated that germination efficiency could be quantified as an EC_50_ value using Michaelis-Menten kinetics (51, 52, 59–65).

**Fig 5:**
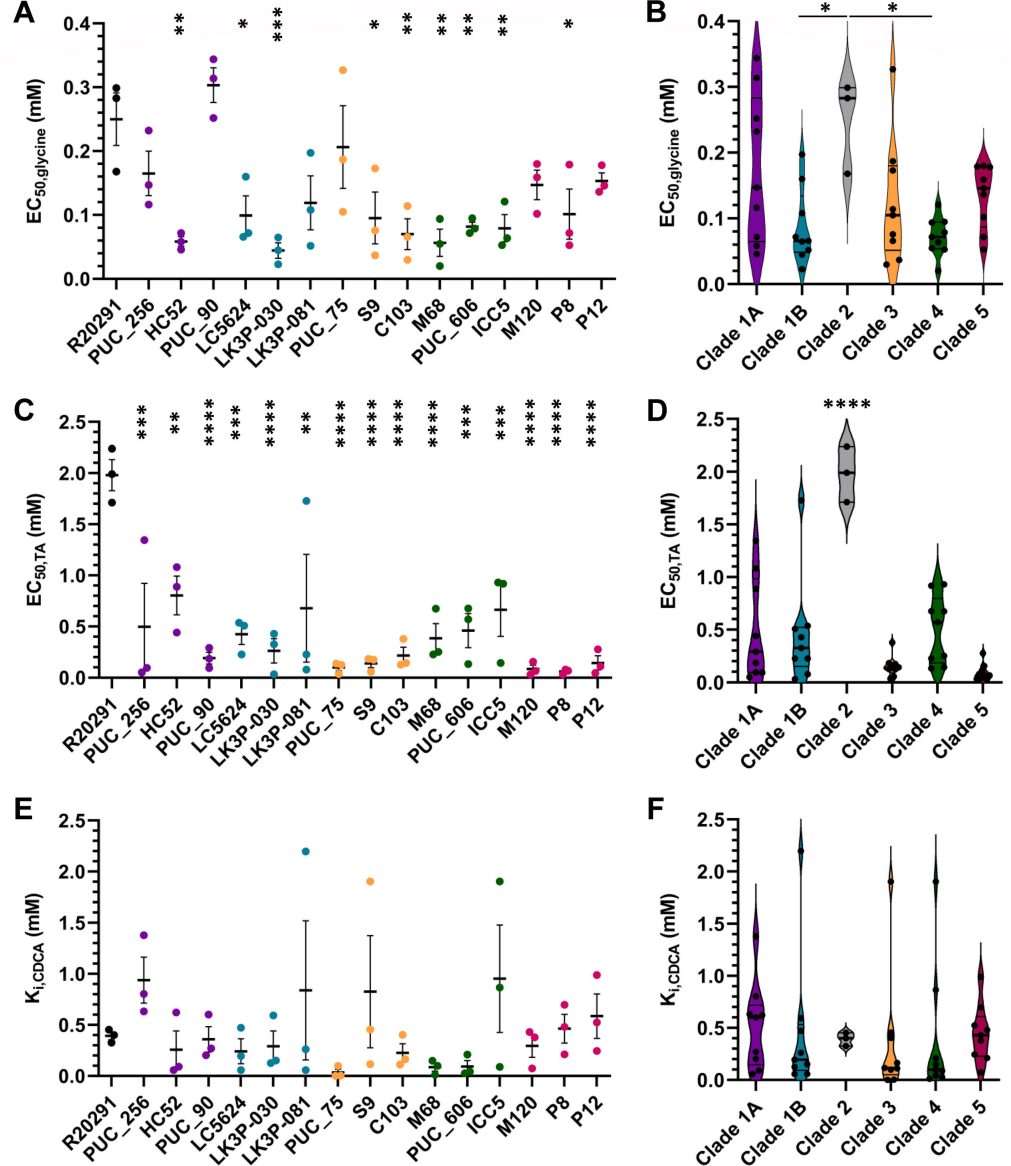
Strain sensitivity to germinants. Germination assays for each strain were performed in the presence of various concentrations of glycine (**A and B**), TA (**C and D**), or TA + CDCA (**E and F**). Germinant sensitivity was calculated using the maximum slope for each condition plotted against (co)-germinant concentration. The data fitted to a linear relationship by taking the inverse of the slope vs concentration plot, and from this, Ki/EC_50_ was calculated with EC_50_ equaling the concentration of germinant that produces the half maximum germination rate. The efficiency of the competitive inhibitor was calculated using the following equation *K*_*i*_ = (inhibitor)/([K_CDCA_/K_TA_] − 1) (61, 62). Data points represent the average from independent biological triplicates with error bars representing the SEM. For A, C, and E, Šidák’s multiple comparisons test (comparing all strains to *C. difficile* R20291) was used, while Tukey’s multiple comparisons test (comparing all clades to each other) was used for panels B, D, and F. Asterisks indicate *P*-values of * ≤0.05, ** ≤0.02, *** ≤0.01, and **** ≤0.0001.

All strains except for *C. difficile* PUC_90 (clade 1A) had a lower EC_50_ value for glycine (the germinant is more potent) compared to the *C. difficile* R20291 strain (clade 2), which had an EC_50,glycine_ of 0.25 mM (Fig. 5A). Clade 1A strains showed the most variation in EC_50,glycine_ with values ranging from 0.06 mM (*C. difficile* HC52) to 0.30 mM (*C. difficile* PUC_90). Clade 4 strains were the most consistent with EC_50,glycine_ values ranging from 0.05 mM to 0.08 mM. The average EC_50,glycine_ value for each clade fell between 0.07 mM and 0.18 mM indicating that glycine sensitivity is consistent between the strains (Fig. 5B). The EC_50,TA_ value for all strains ranged from 0.10 mM (*C. difficile* P8, clade 5) to 0.60 mM (*C. difficile* HC52, clade 1A), all of which were lower than the *C. difficile* R20291 strain (clade 2, 2.0 mM; Fig. 5C). The largest variation was observed in the clade 1A strains with EC_50,TA_ values ranging from 0.20 mM (*C. difficile* PUC_90) to 0.80 mM (*C. difficile* HC52). Clade 5 strains had the least variation with the EC_50,TA_ values ranging from 0.10 mM (*C. difficile* P8) to 0.15 mM (*C. difficile* P12). At the clade level, EC_50,TA_ remained consistent with only slight variations between 0.10 mM for clades 3 and 5 and 0.60 mM for clade 4 (Fig. 5D).

In addition to observing the germination of the strains in response to two of the most efficient germinants, we also determined the response to a known competitive inhibitor of TA-mediated spore germination, CDCA. *K*_*i*_ values for each strain ranged from 0.10 mM for *C. difficile* PUC_75 to 0.90 mM for *C. difficile* strains PUC_256 and ICC5 (Fig. 5E). The *C. difficile* R20291 strain had a *K*_*i*_ value of 0.40 mM. There were no major differences in inhibitor sensitivity compared to the control. Additionally, these data are consistent between clades with only a 0.10 mM difference between the least (0.40 mM, clade 3) and greatest (0.50 mM, clade 1A) *K*_*i*_ values (Fig. 5F).

The bile acid and amino acid co-germinant signals are recognized in *C. difficile* by the pseudoproteases CspC and CspA, respectively (52, 94). These signals are transmitted to CspB, which then activates the cortex lytic enzyme, SleC (95–97). To determine if there was any genetic variation in the proteins responsible for initiating germination, we aligned the CspBA, CspC, and SleC sequences (52, 94, 95, 97, 98). The alignments (Fig. S11 to S13) of each protein revealed some variations compared to the *C. difficile* R20291 strain (clade 2), but none matched any residues that were found to influence germinant sensitivity (95, 98).

### Secretion of toxins A and B varies in the *C. difficile* clinical isolates

**Fig 6:**
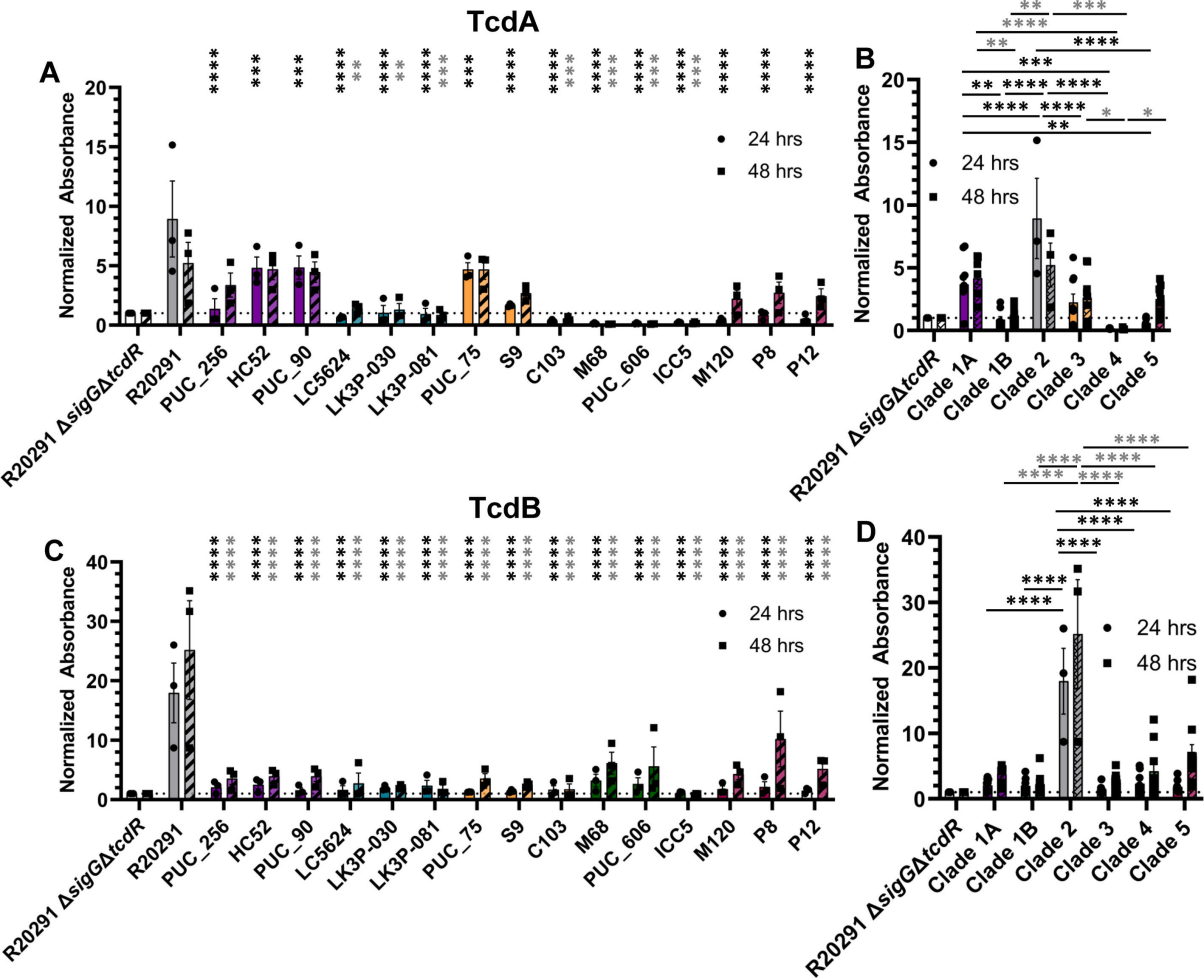
Toxin A/B levels of strains. Exponential-phase cultures of each strain were inoculated into BHIS and grown for 48 hours. At 24 and 48 hours post-inoculation, samples were collected and frozen. Thawed samples were tested for the presence of toxins A (**A and B**) and B (**B and D**) using a commercial ELISA kit from Epitope Diagnostics. Data points represent the average from independent biological triplicates with error bars representing the SEM. The limit of detection as determined by the absorbance of the *C. difficile* R20291 Δ*sigG*Δ*tcdR* strain is indicated by a dotted line. A repeated measure (RM) two-way ANOVA was used to compare each strain to *C. difficile* R20291 (**A and C**) or to compare all clades to each other (**B and D**). Asterisks indicate *P*-values of * ≤0.05, ** ≤0.02, *** ≤0.01, and **** ≤0.0001 with black asterisks designating comparisons between the 24-hour samples and gray asterisks designating comparisons between 48-hour samples.

Here, we used TcdA- and TcdB-specific ELISAs to assess the levels of toxins secreted by the strains. At 24 hours, negligible levels of TcdA were detected in all strains except *C. difficile* strains HC52 (clade 1A), PUC_90 (clade 1A), and PUC_75 (clade 3), which produced an approximately fivefold greater signal than the *C. difficile* R20291 Δ*sigG*Δ*tcdR* negative control (Fig. 6A). At 48 hours, the clades 1A, 3 (except *C. difficile* C103), and 5 strains produced a TcdA signal approximately two- to fivefold greater than the negative control. At both 24 and 48 hours, clade 1A strains produced the most TcdA with a signal approximately fivefold greater than the negative control strain, while absorbance values for the clade 4 strains were below the limit of detection (Fig. 6B). TcdB levels were similar at both 24 and 48 hours between the clades 1A, 1B, and 3 strains (Fig. 6C). Clade 4 strains, *C. difficile* M68 and PUC_606, produced approximately onefold increase in TcdB signal relative to the negative control strain at 24 hours and approximately sixfold signal increase at 48 hours. The clade 5 strains show the largest increase in TcdB signal at 48 hours with approximately 5- to 11-fold increase compared to the negative control strain. TcdB levels were similar across all clades at each tested timepoint at approximately one- to twofold greater than the negative control strain (Fig. 6D). Toxin levels for all strains/clades at both 24 and 48 hours were lower than the *C. difficile* R20291 strain (Fig. 6).

Genomic analysis of the PaLoc (data not shown) indicates high variability within the *tcdA* gene across the clinical strains. Notably, *C. difficile* C103 (clade 3) contains a large (~9 kb) insert between the *tcdA* and *tcdB* genes within the PaLoc, providing a reason for the lack of TcdA signal. Additionally, *C. difficile* ICC5 (clade 4) is missing the vast majority of this locus, which explains the absence of both TcdA and TcdB signals in this strain.

### *C. difficile* strains are equally resistant to bile acid toxicity

**Fig 7:**
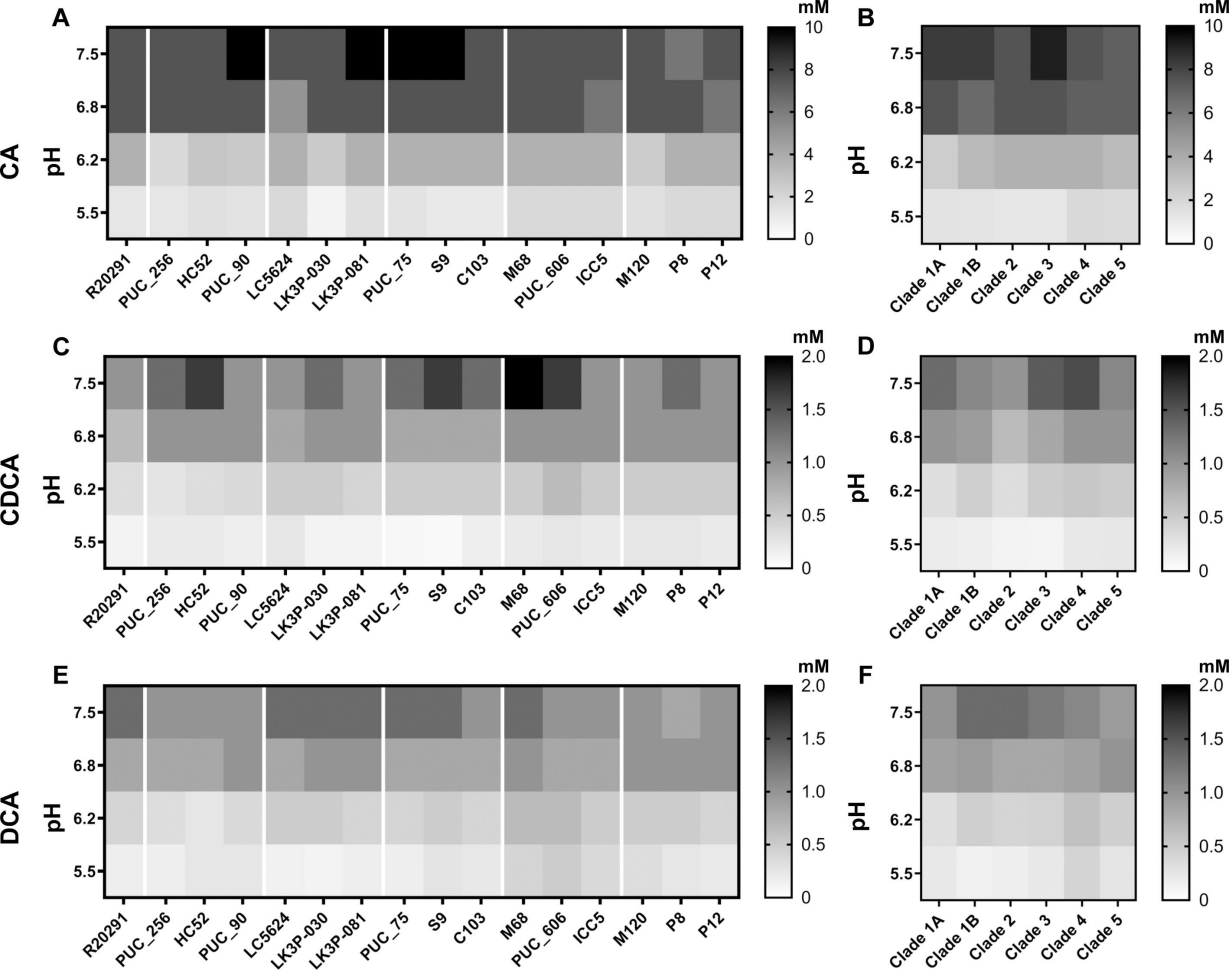
Bile salt sensitivity of strains. Exponential-phase cultures of each strain were inoculated into BHIS of the indicated pH and concentration of CA (**A and B**), CDCA (**C and D**), and DCA (**E and F**). MICs were assessed by the presence/absence of growth after ~18 hours. Black represents the highest tested concentration of bile salt value, while white represents no inhibition. Data represent the average from independent biological triplicates.

Several bile acids are known to inhibit *C. difficile* growth (93, 99, 100). Thus, we sought to determine if any of our strains were equally susceptible to CA (the primary bile acid used to generate TA), DCA (a secondary bile acid generated from 7α-dehydroxylation of CA), and CDCA (an analog of CA) using MIC assays. To mimic the various regions of the colon encountered by *C. difficile*, each assay was performed at pH 7.5, 6.8, 6.2, and 5.5 (101–103). The results indicated little variation in bile acid sensitivity between strains (Fig. 7). At the most neutral pH, the MIC for CA was 7.5 mM. As the pH became more acidic, the MIC decreased eightfold to 0.94 mM (Fig. 7A). A similar trend was seen for CDCA and DCA with an MIC of 1 mM at pH 7.5 and an eightfold decrease in MIC at pH 5.5 (Fig. 7C and E). This data, when grouped by clade, again found no difference in bile acid sensitivity between clades (Fig. 7B, D and F).

### *C. difficile* strains vary in their ability to modify taurine-based bile salts

**Fig 8:**
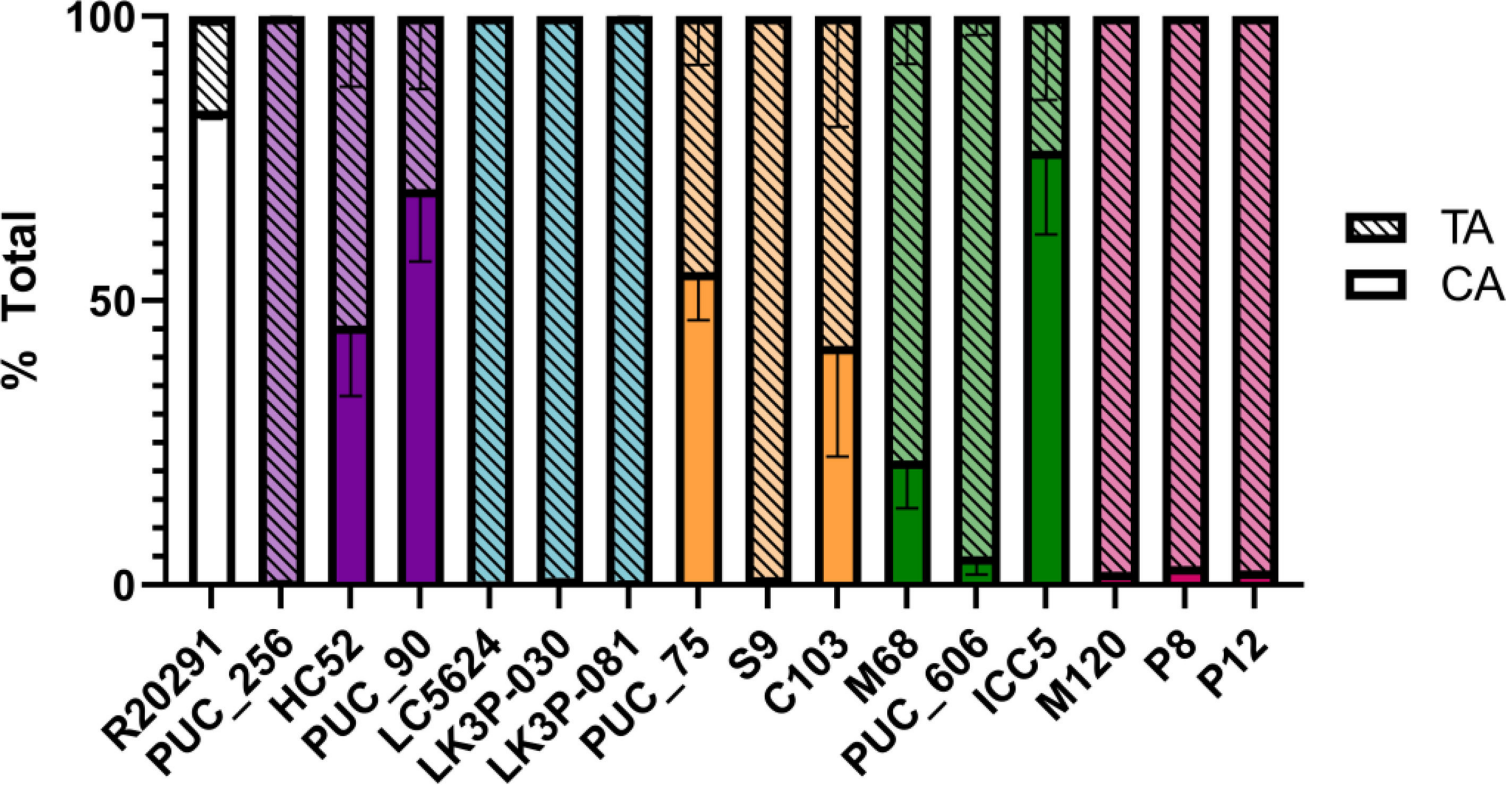
BSHA. Each strain was grown in the presence of 1 mM TA and incubated for 24 hours. The bile salts present in each culture following incubation were identified/quantified by reverse-phase HPLC. Percent deconjugation was calculated using the following formula: percent deconjugation = CA/(TA + CA). Data points represent the average from independent biological triplicates with error bars representing the SEM.

Previous work from our lab demonstrated that some *C. difficile* strains deconjugate taurine-conjugated bile salts (54). While it is currently unknown how this activity contributes to disease outcomes, the introduction of free taurine into the host could impact disease progression. Because the examined strains had various levels of activity, we tested the BSHA of our strains using TA as a substrate. Each strain was incubated with TA, and the amount of its deconjugated product (CA) was quantified (Fig. 8). *C. difficile* PUC_90 (clade 1A) and *C. difficile* ICC5 (clade 4) processed TA at an efficiency comparable to the *C. difficile* control (~70%–80%). *C. difficile* strains HC52 (clade 1A), S9 (clade 3), and PUC_606 (clade 4) had little activity against TA, while the remaining clade 1A, 3, and 4 strains had some activity (though not to the same level as *C. difficile* R20291 [clade 2]). Interestingly, all strains from both clades 1B and 5 had low levels of BSHA against TA.

In prior work, we found that strains that did not deconjugate TA could process TDCA (54). Thus, we tested if clades 1B and 5 strains could deconjugate TDCA (Fig. S14). No detectable amount of DCA was generated by these strains. Taken together, this suggests that BSH activity may not be a core phenotype shared among strains.

### *C. difficile* strains show similar surface motility

Some clade 5 strains have distinct growth morphologies compared to members of other clades (69). We tested surface motility for each strain to determine if we could detect similar morphologies (Fig. S15A through F). Strains from all clades spread similarly from the point of inoculation (Fig. S15G). The size of the protrusions from the central growth ring was the most striking variation, with *C. difficile* LK3P-030 (clade 1B) having the largest projections, and *C. difficile* M68 (clade 4) producing virtually no projections. The quantification of these projections, however, indicated that these differences, while visually apparent, were not statistically significant (Fig. S15H). As previously reported (69), the clade 5 strains had some asymmetric growth of the extensions, which is especially evident in the *C. difficile* P12 strain (clade 5; Fig. S15Fii).

### Most genes known to contribute to the observed phenotypes are found in the core genome

To determine which phenotypes are core processes, we identified the core genome for our strains using Spine (Table S2) (48). While developed for use in *Pseudomonas aeruginosa*, several studies have reported its utility for *C. difficile* (35, 104–107). The genes encoding each protein aligned in this paper were compared to the list of core genome genes produced from the Spine analysis. Most of these genes (*ccpa*, *xylA*, *xylB*, *xylR* (*CD630_RS16335*), *cspBA*, *cspC*, and *sleC*) were found in the set of core genes for our strains, confirming their classification as core processes. Unsurprisingly, the genes corresponding to most variable phenotypes (*treA*, *treR*, *tcdA*, and *tcdB*) were not included in the core genome.

## DISCUSSION

*C. difficile* is a highly variable species with an open pan-genome (22). Phylogenetic analysis indicates significant evolutionary changes between the five main clades as indicated by widespread homologous recombination and horizontal gene transfer (23, 24). Previous research has shown that even members of the same clade/ribotype had varying phenotypes (e.g., toxin activity) (108, 109). Additionally, several studies have observed an apparent correlation between ribotype and clinical outcome, with RT106 (clade 1/2) and RT078 (clade 5) being associated with the worst outcomes/recurrence compared to the commonly studied RT027 (clade 2) (34–42, 110). We hypothesized that despite evolutionary variation, *C. difficile* strains would have core phenotypes that are central to *C. difficile* biology and consequently similar across clades/ribotypes.

All strains had similar growth rates in the rich medium tested, indicating that any differences observed between strains are not due to differences in growth while in liquid BHIS during experimental setup. When growth was assessed in a minimal medium and compared to the laboratory-adapted *C. difficile* R20291 strain (clade 2), most of the clinical strains (except for the clade 1A strains which had similar generation times compared to *C. difficile* R20291) showed decreased growth rates in glucose-containing CDMM and increased growth rates in fructose-/xylose-/trehalose-containing medium. Between the clades, there was some variation, especially in the clade 4 strains which had consistently decreased growth rates in all tested media types. While alignments of the carbohydrate metabolism protein sequences revealed some genetic variation, these variations are not consistent with the observed phenotypic differences. This suggests that variations within the protein sequences themselves are not sufficient to explain the observed phenotypic variation.

Notably, another study reported that RT023 (clade 3 strains) was unable to grow on CDMM-tre (111). Our clade 3 strains grew relatively well in CDMM-tre, which may be the result of differences in experimental setup. Specifically, the strains in this study were grown in CDMM-tre for 24 hours followed by a subculture into the same media and a further 12 hours of growth prior to inoculation of the experimental culture. In the Midani study (111), cells were grown solely in BHIS prior to dilution into the experimental culture. If trehalose metabolism occurs in these strains via an unconventional pathway, as suggested by our genomic data (these strains lack an intact version of the canonical trehalose operon), processing of trehalose may be less efficient during the lag phase of growth. The additional time spent in the trehalose-supplemented minimal medium likely allowed the strains to adapt to the nutrient-limited conditions, reducing the lag phase and resulting in growth observable over 22 hours. Additionally, variation in the makeup of CDMM (defined amino acids vs casamino acids, additional vitamins, or variable lots of each constituent) or unknown strain-specific divergences could explain the differing results between studies.

For the number of spores produced by each strain over a 48-hour period, only two strains (*C. difficile* HC52: clade 1A and *C. difficile* LK3P-081: clade 1B) demonstrated any statistically significant differences compared to *C. difficile* R20291; the largest difference observed between the strains was ~10-fold. Whether this difference has any biological relevance remains to be seen. Given that some studies of *C. difficile* infection in mice report disease development with as few as 100 spores, this difference is unlikely to impact disease formation/progression but might contribute to altered disease spread/recurrence (6, 112).

We observed a decreased EC_50_ value for both TA and glycine for most of our strains compared to the control. This indicates increased sensitivity to these germinants. The EC_50,glycine_ values are less than or similar to the physiologically relevant concentration of glycine (~0.1–0.3 mM), suggesting that these strains have become better adapted to sensing co-germinants in the mammalian gut (113–115). Additionally, the EC_50_,_TA_ values are similar to the estimated concentration of TA in the colon following antibiotic treatment, suggesting that TA-mediated germination in all tested strains occurs efficiently in the gut (116, 117). Previously, the amounts of the Csp and SleC germination proteins present in *C. difficile* spores have been quantified (52). While not tested in this study, variation in the relative amounts of each protein found in each strain or changes in the transcriptional regulation of the proteins could contribute to the observed differences. Moreover, differences in spore coat composition could influence germinant permeability as seen in *Bacillus anthracis* (118). Additionally, we observed no differences in germination in the presence of CDCA, a competitive inhibitor of TA-mediated spore germination. CDCA was an effective inhibitor of TA-mediated germination in all tested strains. These results suggest that anti-germination-based therapies could be broadly applicable in the treatment of CDI. In support of this, recent work on the bile salt analogs, CamSA and CaPA, shows protection against CDI in mouse models of infection (119, 120).

Investigations of *C. difficile* strains isolated during outbreaks have assessed genotypic diversity of ribotypes/sequence types and reported upon virulence (i.e., toxin A/B) related phenotypes (121–123). These studies identified variation within the *C. difficile* PaLoc, which is often correlated to variation in disease severity. This is consistent with our phenotypic observations *in vitro*, especially regarding the variable levels of TcdA secreted by each clade. Notably, the clade 4 strains do not secrete significant amounts of TcdA consistent with their designation as an A^−^B^+^ toxinotype (124). Interestingly, the clinical strains all present with lower toxin levels compared to the *C. difficile* R20291 strain (clade 2). This could be the result of strain specificity in the antibodies from the ELISA kit used to assess toxin levels. Alternatively, differences in toxin secretion could contribute to the variable phenotypes, a possibility that would require further analysis of PaLoc gene expression in each of these strains.

Each of the strains was assessed for its ability to resist the toxic effects of certain bile acids. This assay was performed at various pHs to mimic conditions experienced by vegetative cells in the colon (101–103). We observed little variation in MIC when compared to *C. difficile* R20291 or between strains. As expected, the MIC of each bile salt increased with pH, reflecting the deprotonation of each bile acid (more negative charges), which limits its ability to interact with and disrupt the negatively charged bacterial membrane. At higher pH values, the MIC values for these strains are well above the physiological concentrations of CA, CDCA, and DCA in the colon (~0.12–0.23 mM) (125). This suggests that bile acid inhibition could be primarily effective in lower pH environments. The lack of variation suggests that bile acid toxicity is a core phenotype for *C. difficile*. Moreover, our results suggest that these bile acids may have different effects on *C. difficile* growth depending on the location within the gut.

Recent work from our lab found that *C. difficile* is capable of processing taurine-conjugated bile salts by removing the taurine group (54). When this assay was performed on the strains in this study, they showed varying abilities to deconjugate TA, with clades 1B and 5 members showing low processing levels. These same strains could also not process TDCA, indicating that the proteins responsible for this behavior are either missing, are not expressed, or have different substrates than the two tested here. Interestingly, this phenotype is unique to the clades 1B and 5 strains, which we previously noted have been associated with more severe/recurrent CDI. Because a bile salt hydrolase has not yet been identified in *C. difficile*, it remains unclear if BSHA, or the lack thereof, is relevant to the clinical outcome of CDI. Regardless, because not all the tested strains demonstrated BSHA, this may not be a core phenotype.

Most of the variation seen within each assay is observed in comparison to the control strain, *C. difficile* R20291 (clade 2). This strain was isolated during the Stoke-Manderville outbreak in the early 2000s, has been passed between laboratories, and may have become a laboratory-adapted strain (though still virulent in animal models) (126). Observed differences correspond to an increased fitness of the clinical isolates compared to the *C. difficile* R20291 strain (clade 2) as indicated by increased growth rates in fructose-/xylose-/trehalose-containing medium and increased sensitivity to germinants. This could indicate either a loss of some functions within the *C. difficile* R20291 strain or that *C. difficile*, as a species, has evolved to become more fit in the gut.

Spine analysis of our strains indicated that most of the genes known to encode proteins involved in the various tested phenotypes are members of the core genome. These proteins have functions related to global metabolism regulation, xylose usage, and germination. Additionally, proteins responsible for regulating the *C. difficile* sporulation program (*spo0A*, *sigE*, *sigF*, *sigG*, *etc*.) are also found in the core genome. This supports our designation of these phenotypes as core processes. Consistent with previous reports on the variation in trehalose metabolism and the non-typical arrangement of the trehalose metabolism genes in our clades 3 and 5 strains (Fig. S9 and S10), the *treR* and *treA* genes were not present in the core genome (25, 88, 111). This suggests, consistent with previous findings, that trehalose metabolism is highly variable between species and likely contributes to the variation in disease severity/recurrence observed between strains (25, 88, 89, 111).

When considering the strains independently of *C. difficile* R20291 (clade 2), we observed remarkable phenotypic similarity between strains and no major patterns corresponding to clade/ribotype. This is true of most tested phenotypes except for carbohydrate use, toxin production, and BSHA. This is consistent with previous research that demonstrates that *C. difficile* metabolism and toxin production are highly variable between strains (121, 122, 127, 128). Only identifying the factor responsible for BSHA will test the hypothesis that it also is not a core process. Because CDI severity has been shown to be highly variable within individual outbreaks, we hypothesize that those phenotypes classified here as non-core processes contribute more to variation than do the core processes. This is further supported by an *in vivo* study in which a set of accessory genes could be correlated with high virulence in mice infected with various clinical isolates of *C. difficile* that shared similar core genomes (129). Taken together, these data suggest that the previously observed relationships between ribotype and CDI severity may not be due to changes in these core phenotypes but rather to other influences including currently unidentified host factors.

This study focused only on a small portion of the known *C. difficile* strains and a limited number of phenotypes. Examples of other relevant phenotypes not tested in this paper include cytotoxicity, adherence, serotype, O_2_ tolerance, and outgrowth of a cell post-spore germination (130–132). Variation in these phenotypes between strains would likely indicate a contribution to disease severity. Further analysis of phenotypic variation between strains of all ribotypes/clades both *in vivo* and *in vitro* will expand upon what we have learned here and provide valuable insight into how *C. difficile* might manifest itself in a clinical setting.

## Data Availability

Long-read and Illumina data for all strains have been uploaded to the NCBI BioProject database under accession PRJNA1252054.

## SUPPLEMENTAL MATERIAL

Supplemental figures, part I aem.00964-25-s0001.pdf Figures S1 to S8.

Supplemental figures, part II aem.00964-25-s0002.pdf Figures S9 to S15.

Table S1 and legends aem.00964-25-s0003.docx Legends for Fig. S1 to S15; Table S1, strains, primers, and plasmids used in ELISA.

Table S2 aem.00964-25-s0004.xlsx Core genome list of genes.

ASM does not own the copyrights to Supplemental Material that may be linked to, or accessed through, an article. The authors have granted ASM a non-exclusive, world-wide license to publish the Supplemental Material files. Please contact the corresponding author directly for reuse.

## References

[1] Setlow P, Wang S, Li YQ. 2017. Germination of spores of the orders Bacillales and Clostridiales. Annu Rev Microbiol 71:459–477. doi:10.1146/annurev-micro-090816-093558 28697670

[2] Deakin LJ, Clare S, Fagan RP, Dawson LF, Pickard DJ, West MR, Wren BW, Fairweather NF, Dougan G, Lawley TD. 2012. The Clostridium difficile spo0A gene is a persistence and transmission factor. Infect Immun 80:2704–2711. doi:10.1128/IAI.00147-12 22615253 PMC3434595

[3] Nerber HN, Sorg JA. 2021. The small acid-soluble proteins of Clostridioides difficile are important for UV resistance and serve as a check point for sporulation. PLoS Pathog 17:e1009516. doi:10.1371/journal.ppat.1009516 34496003 PMC8452069

[4] Rodriguez-Palacios A, Lejeune JT. 2011. Moist-heat resistance, spore aging, and superdormancy in Clostridium difficile. Appl Environ Microbiol 77:3085–3091. doi:10.1128/AEM.01589-10 21398481 PMC3126382

[5] Kochan TJ, Shoshiev MS, Hastie JL, Somers MJ, Plotnick YM, Gutierrez-Munoz DF, Foss ED, Schubert AM, Smith AD, Zimmerman SK, Carlson PE Jr, Hanna PC. 2018. Germinant synergy facilitates Clostridium difficile spore germination under physiological conditions. mSphere 3:e00335-18. doi:10.1128/mSphere.00335-18 30185513 PMC6126144

[6] Koenigsknecht MJ, Theriot CM, Bergin IL, Schumacher CA, Schloss PD, Young VB. 2015. Dynamics and establishment of Clostridium difficile infection in the murine gastrointestinal tract. Infect Immun 83:934–941. doi:10.1128/IAI.02768-14 25534943 PMC4333439

[7] Giel JL, Sorg JA, Sonenshein AL, Zhu J. 2010. Metabolism of bile salts in mice influences spore germination in Clostridium difficile. PLoS ONE 5:e8740. doi:10.1371/journal.pone.0008740 20090901 PMC2806926

[8] Abt MC, McKenney PT, Pamer EG. 2016. Clostridium difficile colitis: pathogenesis and host defence. Nat Rev Microbiol 14:609–620. doi:10.1038/nrmicro.2016.108 27573580 PMC5109054

[9] McDonald LC, Gerding DN, Johnson S, Bakken JS, Carroll KC, Coffin SE, Dubberke ER, Garey KW, Gould CV, Kelly C, Loo V, Shaklee Sammons J, Sandora TJ, Wilcox MH. 2018. Clinical practice guidelines for Clostridium difficile infection in adults and children: 2017 update by the Infectious Diseases Society of America (IDSA) and Society for Healthcare Epidemiology of America (SHEA). Clin Infect Dis 66:e1–e48. doi:10.1093/cid/cix1085 29462280 PMC6018983

[10] CDC. 2019. Antibiotic resistance threats in the United States, 2019. U.S. Department of Health and Human Services.

[11] Cohen SH, Tang YJ, Silva J Jr. 2000. Analysis of the pathogenicity locus in Clostridium difficile strains. J Infect Dis 181:659–663. doi:10.1086/315248 10669352

[12] Bagdasarian N, Rao K, Malani PN. 2015. Diagnosis and treatment of Clostridium difficile in adults: a systematic review. JAMA 313:398–408. doi:10.1001/jama.2014.17103 25626036 PMC6561347

[13] Rupnik M, Janezic S. 2016. An update on Clostridium difficile toxinotyping. J Clin Microbiol 54:13–18. doi:10.1128/JCM.02083-15 26511734 PMC4702747

[14] Sebaihia M, Wren BW, Mullany P, Fairweather NF, Minton N, Stabler R, Thomson NR, Roberts AP, Cerdeño-Tárraga AM, Wang H, et al.. 2006. The multidrug-resistant human pathogen Clostridium difficile has a highly mobile, mosaic genome. Nat Genet 38:779–786. doi:10.1038/ng1830 16804543

[15] EnteroBase. 2024. EnteroBase. Available from: https://enterobase.warwick.ac.uk/species/index/clostridium

[16] Information NCfB. 2024. NCBI Genome. Available from: https://www.ncbi.nlm.nih.gov/datasets/genome/?taxon=1496

[17] Perumalsamy S, Riley TV. 2021. Molecular epidemiology of Clostridioides difficile infections in children. J Pediatric Infect Dis Soc 10:S34–S40. doi:10.1093/jpids/piab057 34791401

[18] He M, Sebaihia M, Lawley TD, Stabler RA, Dawson LF, Martin MJ, Holt KE, Seth-Smith HMB, Quail MA, Rance R, Brooks K, Churcher C, Harris D, Bentley SD, Burrows C, Clark L, Corton C, Murray V, Rose G, Thurston S, van Tonder A, Walker D, Wren BW, Dougan G, Parkhill J. 2010. Evolutionary dynamics of Clostridium difficile over short and long time scales. Proc Natl Acad Sci USA 107:7527–7532. doi:10.1073/pnas.0914322107 20368420 PMC2867753

[19] Stabler RA, He M, Dawson L, Martin M, Valiente E, Corton C, Lawley TD, Sebaihia M, Quail MA, Rose G, Gerding DN, Gibert M, Popoff MR, Parkhill J, Dougan G, Wren BW. 2009. Comparative genome and phenotypic analysis of Clostridium difficile 027 strains provides insight into the evolution of a hypervirulent bacterium. Genome Biol 10:R102. doi:10.1186/gb-2009-10-9-r102 19781061 PMC2768977

[20] Bletz S, Janezic S, Harmsen D, Rupnik M, Mellmann A. 2018. Defining and evaluating a core genome multilocus sequence typing scheme for genome-wide typing of Clostridium difficile. J Clin Microbiol 56:e01987-17. doi:10.1128/JCM.01987-17 29618503 PMC5971537

[21] Golchha NC, Nighojkar A, Nighojkar S. 2022. Redefining genomic view of Clostridioides difficile through pangenome analysis and identification of drug targets from its core genome. Drug Target Insights 16:17–24. doi:10.33393/dti.2022.2469 36415217 PMC9669665

[22] Norsigian CJ, Danhof HA, Brand CK, Midani FS, Broddrick JT, Savidge TC, Britton RA, Palsson BO, Spinler JK, Monk JM. 2022. Systems biology approach to functionally assess the Clostridioides difficile pangenome reveals genetic diversity with discriminatory power. Proc Natl Acad Sci USA 119:e2119396119. doi:10.1073/pnas.2119396119 35476524 PMC9170149

[23] Didelot X, Eyre DW, Cule M, Ip CLC, Ansari MA, Griffiths D, Vaughan A, O’Connor L, Golubchik T, Batty EM, Piazza P, Wilson DJ, Bowden R, Donnelly PJ, Dingle KE, Wilcox M, Walker AS, Crook DW, Peto TEA, Harding RM. 2012. Microevolutionary analysis of Clostridium difficile genomes to investigate transmission. Genome Biol 13:R118. doi:10.1186/gb-2012-13-12-r118 23259504 PMC4056369

[24] Knight DR, Elliott B, Chang BJ, Perkins TT, Riley TV. 2015. Diversity and evolution in the genome of Clostridium difficile. Clin Microbiol Rev 28:721–741. doi:10.1128/CMR.00127-14 26085550 PMC4475645

[25] Collins J, Robinson C, Danhof H, Knetsch CW, van Leeuwen HC, Lawley TD, Auchtung JM, Britton RA. 2018. Dietary trehalose enhances virulence of epidemic Clostridium difficile. Nature 553:291–294. doi:10.1038/nature25178 29310122 PMC5984069

[26] Knight DR, Imwattana K, Kullin B, Guerrero-Araya E, Paredes-Sabja D, Didelot X, Dingle KE, Eyre DW, Rodríguez C, Riley TV. 2021. Major genetic discontinuity and novel toxigenic species in Clostridioides difficile taxonomy. Elife 10:e64325. doi:10.7554/eLife.64325 34114561 PMC8241443

[27] Griffiths D, Fawley W, Kachrimanidou M, Bowden R, Crook DW, Fung R, Golubchik T, Harding RM, Jeffery KJM, Jolley KA, Kirton R, Peto TE, Rees G, Stoesser N, Vaughan A, Walker AS, Young BC, Wilcox M, Dingle KE. 2010. Multilocus sequence typing of Clostridium difficile. J Clin Microbiol 48:770–778. doi:10.1128/JCM.01796-09 20042623 PMC2832416

[28] Alonso R, Martín A, Peláez T, Marín M, Rodríguez-Creixéms M, Bouza E. 2005. An improved protocol for pulsed-field gel electrophoresis typing of Clostridium difficile. J Med Microbiol 54:155–157. doi:10.1099/jmm.0.45808-0 15673509

[29] Gürtler V. 1993. Typing of Clostridium difficile strains by PCR-amplification of variable length 16S-23S rDNA spacer regions. J Gen Microbiol 139:3089–3097. doi:10.1099/00221287-139-12-3089 7510324

[30] Toma S, Lesiak G, Magus M, Lo HL, Delmée M. 1988. Serotyping of Clostridium difficile. J Clin Microbiol 26:426–428. doi:10.1128/jcm.26.3.426-428.1988 2833528 PMC266306

[31] Sambol SP, Johnson S, Gerding DN. 2016. Restriction endonuclease analysis typing of Clostridium difficile isolates. Methods Mol Biol 1476:1–13. doi:10.1007/978-1-4939-6361-4_1 27507329

[32] Martin MJ, Clare S, Goulding D, Faulds-Pain A, Barquist L, Browne HP, Pettit L, Dougan G, Lawley TD, Wren BW. 2013. The agr locus regulates virulence and colonization genes in Clostridium difficile 027. J Bacteriol 195:3672–3681. doi:10.1128/JB.00473-13 23772065 PMC3754575

[33] He M, Miyajima F, Roberts P, Ellison L, Pickard DJ, Martin MJ, Connor TR, Harris SR, Fairley D, Bamford KB, et al.. 2013. Emergence and global spread of epidemic healthcare-associated Clostridium difficile. Nat Genet 45:109–113. doi:10.1038/ng.2478 23222960 PMC3605770

[34] Knight DR, Kullin B, Androga GO, Barbut F, Eckert C, Johnson S, Spigaglia P, Tateda K, Tsai PJ, Riley TV. 2019. Evolutionary and genomic insights into Clostridioides difficile sequence type 11: a diverse zoonotic and antimicrobial-resistant lineage of global one health importance. MBio 10:e00446-19. doi:10.1128/mBio.00446-19 30992351 PMC6469969

[35] Kociolek LK, Gerding DN, Hecht DW, Ozer EA. 2018. Comparative genomics analysis of Clostridium difficile epidemic strain DH/NAP11/106. Microbes Infect 20:245–253. doi:10.1016/j.micinf.2018.01.004 29391259 PMC5911408

[36] Tsai CS, Cheng YL, Chen JS, Tsai PJ, Tsai BY, Hsu BM, Huang IH. 2022. Hypervirulent Clostridioides difficile RT078 lineage isolates from the river: a potential reservoir for environmental transmission. J Microbiol Immunol Infect 55:977–981. doi:10.1016/j.jmii.2022.05.002 35739056

[37] Tsai BY, Ko WC, Chen TH, Wu YC, Lan PH, Chen YH, Hung YP, Tsai PJ. 2016. Zoonotic potential of the Clostridium difficile RT078 family in Taiwan. Anaerobe 41:125–130. doi:10.1016/j.anaerobe.2016.06.002 27292030

[38] Li C, Harmanus C, Zhu D, Meng X, Wang S, Duan J, Liu S, Fu C, Zhou P, Liu R, Wu A, Kuijper EJ, Smits WK, Fu L, Sun X. 2018. Characterization of the virulence of a non-RT027, non-RT078 and binary toxin-positive Clostridium difficile strain associated with severe diarrhea. Emerg Microbes Infect 7:211. doi:10.1038/s41426-018-0211-1 30542069 PMC6291415

[39] Knetsch CW, Kumar N, Forster SC, Connor TR, Browne HP, Harmanus C, Sanders IM, Harris SR, Turner L, Morris T, Perry M, Miyajima F, Roberts P, Pirmohamed M, Songer JG, Weese JS, Indra A, Corver J, Rupnik M, Wren BW, Riley TV, Kuijper EJ, Lawley TD. 2018. Zoonotic transfer of Clostridium difficile harboring antimicrobial resistance between farm animals and humans. J Clin Microbiol 56:e01384-17. doi:10.1128/JCM.01384-17 29237792 PMC5824051

[40] Du T, Choi KB, Silva A, Golding GR, Pelude L, Hizon R, Al-Rawahi GN, Brooks J, Chow B, Collet JC, et al.. 2022. Characterization of healthcare-associated and community-associated Clostridioides difficile infections among adults, Canada, 2015-2019. Emerg Infect Dis 28:1128–1136. doi:10.3201/eid2806.212262 35470794 PMC9155897

[41] Almutairi MS, Gonzales-Luna AJ, Alnezary FS, Fallatah SB, Alam MJ, Begum K, Garey KW. 2021. Comparative clinical outcomes evaluation of hospitalized patients infected with Clostridioides difficile ribotype 106 vs. other toxigenic strains. Anaerobe 72:102440. doi:10.1016/j.anaerobe.2021.102440 34461273 PMC8678208

[42] Carlson TJ, Blasingame D, Gonzales-Luna AJ, Alnezary F, Garey KW. 2020. Clostridioides difficile ribotype 106: a systematic review of the antimicrobial susceptibility, genetics, and clinical outcomes of this common worldwide strain. Anaerobe 62:102142. doi:10.1016/j.anaerobe.2019.102142 32007682 PMC7153973

[43] Brehm JN, Sorg JA. 2024. Theophylline-based control of repA on a Clostridioides difficile plasmid for use in allelic exchange. Anaerobe 88:102858. doi:10.1016/j.anaerobe.2024.102858 38692475 PMC11984826

[44] Kolmogorov M, Yuan J, Lin Y, Pevzner PA. 2019. Assembly of long, error-prone reads using repeat graphs. Nat Biotechnol 37:540–546. doi:10.1038/s41587-019-0072-8 30936562

[45] Lin Y, Yuan J, Kolmogorov M, Shen MW, Chaisson M, Pevzner PA. 2016. Assembly of long error-prone reads using de Bruijn graphs. Proc Natl Acad Sci USA 113:E8396–E8405. doi:10.1073/pnas.1604560113 27956617 PMC5206522

[46] Bushnell B. 2014. BBMap: a fast, accurate, splice-aware aligner. Abstr Conference: 9th Annual Genomics of Energy & Environment Meeting; Walnut Creek, CA

[47] Darling AE, Mau B, Perna NT. 2010. progressiveMauve: multiple genome alignment with gene gain, loss and rearrangement. PLoS ONE 5:e11147. doi:10.1371/journal.pone.0011147 20593022 PMC2892488

[48] Ozer EA, Allen JP, Hauser AR. 2014. Characterization of the core and accessory genomes of Pseudomonas aeruginosa using bioinformatic tools Spine and AGEnt. BMC Genomics 15:737. doi:10.1186/1471-2164-15-737 25168460 PMC4155085

[49] Sievers F, Wilm A, Dineen D, Gibson TJ, Karplus K, Li W, Lopez R, McWilliam H, Remmert M, Söding J, Thompson JD, Higgins DG. 2011. Fast, scalable generation of high-quality protein multiple sequence alignments using clustal omega. Mol Syst Biol 7:539. doi:10.1038/msb.2011.75 21988835 PMC3261699

[50] Bhattacharjee D, Francis MB, Ding X, McAllister KN, Shrestha R, Sorg JA. 2016. Reexamining the germination phenotypes of several Clostridium difficile strains suggests another role for the CspC germinant receptor. J Bacteriol 198:777–786. doi:10.1128/JB.00908-15 PMC4810609 26668265

[51] Shrestha R, Sorg JA. 2018. Hierarchical recognition of amino acid co-germinants during Clostridioides difficile spore germination. Anaerobe 49:41–47. doi:10.1016/j.anaerobe.2017.12.001 29221987 PMC5844826

[52] Shrestha R, Cochran AM, Sorg JA. 2019. The requirement for co-germinants during Clostridium difficile spore germination is influenced by mutations in yabG and cspA. PLoS Pathog 15:e1007681. doi:10.1371/journal.ppat.1007681 30943268 PMC6464247

[53] Baloh M, Sorg JA. 2021. Clostridioides difficile SpoVAD and SpoVAE interact and are required for dipicolinic acid uptake into spores. J Bacteriol 203:e0039421. doi:10.1128/JB.00394-21 34424035 PMC8508128

[54] Aguirre AM, Adegbite AO, Sorg JA. 2022. Clostridioides difficile bile salt hydrolase activity has substrate specificity and affects biofilm formation. NPJ Biofilms Microbiomes 8:94. doi:10.1038/s41522-022-00358-0 36450806 PMC9712596

[55] Baloh M, Nerber HN, Sorg JA. 2022. Imaging Clostridioides difficile spore germination and germination proteins. J Bacteriol 204:e0021022. doi:10.1128/jb.00210-22 35762766 PMC9295549

[56] Nerber HN, Baloh M, Brehm JN, Sorg JA. 2024. The small acid-soluble proteins of Clostridioides difficile regulate sporulation in a SpoIVB2-dependent manner. PLoS Pathog 20:e1012507. doi:10.1371/journal.ppat.1012507 39213448 PMC11392383

[57] Osborne MS, Brehm JN, Olivença C, Cochran AM, Serrano M, Henriques AO, Sorg JA. 2024. The impact of YabG mutations on Clostridioides difficile spore germination and processing of spore substrates. Mol Microbiol 122:534–548. doi:10.1111/mmi.15316 39258427 PMC12016784

[58] Edwards AN, McBride SM. 2017. Determination of the in vitro sporulation frequency of Clostridium difficile. Bio Protoc 7. doi:10.21769/BioProtoc.2125 PMC5432021 28516125

[59] Akoachere M, Squires RC, Nour AM, Angelov L, Brojatsch J, Abel-Santos E. 2007. Identification of an in vivo inhibitor of Bacillus anthracis spore germination. J Biol Chem 282:12112–12118. doi:10.1074/jbc.M611432200 17296608

[60] Ramirez N, Abel-Santos E. 2010. Requirements for germination of Clostridium sordellii spores in vitro. J Bacteriol 192:418–425. doi:10.1128/JB.01226-09 19915025 PMC2805323

[61] Ramirez N, Liggins M, Abel-Santos E. 2010. Kinetic evidence for the presence of putative germination receptors in Clostridium difficile spores. J Bacteriol 192:4215–4222. doi:10.1128/JB.00488-10 20562307 PMC2916422

[62] Sorg JA, Sonenshein AL. 2010. Inhibiting the initiation of Clostridium difficile spore germination using analogs of chenodeoxycholic acid, a bile acid. J Bacteriol 192:4983–4990. doi:10.1128/JB.00610-10 20675492 PMC2944524

[63] Abel-Santos E, Dodatko T. 2007. Differential nucleoside recognition during Bacillus cereus 569 (ATCC 10876) spore germination. New J Chem 31:748. doi:10.1039/b616695d

[64] Dodatko T, Akoachere M, Jimenez N, Alvarez Z, Abel-Santos E. 2010. Dissecting interactions between nucleosides and germination receptors in Bacillus cereus 569 spores. Microbiology (Reading) 156:1244–1255. doi:10.1099/mic.0.030270-0 20035009 PMC2889443

[65] Howerton A, Ramirez N, Abel-Santos E. 2011. Mapping interactions between germinants and Clostridium difficile spores. J Bacteriol 193:274–282. doi:10.1128/JB.00980-10 20971909 PMC3019946

[66] Aguirre AM, Yalcinkaya N, Wu Q, Swennes A, Tessier ME, Roberts P, Miyajima F, Savidge T, Sorg JA. 2021. Bile acid-independent protection against Clostridioides difficile infection. PLoS Pathog 17:e1010015. doi:10.1371/journal.ppat.1010015 34665847 PMC8555850

[67] Hong YJ, Turowski M, Lin JT, Yokoyama WH. 2007. Simultaneous characterization of bile acid, sterols, and determination of acylglycerides in feces from soluble cellulose-fed hamsters using HPLC with evaporative light-scattering detection and APCI-MS. J Agric Food Chem 55:9750–9757. doi:10.1021/jf071798+ 17979236

[68] Torchia EC, Labonté ED, Agellon LB. 2001. Separation and quantitation of bile acids using an isocratic solvent system for high performance liquid chromatography coupled to an evaporative light scattering detector. Anal Biochem 298:293–298. doi:10.1006/abio.2001.5379 11700985

[69] Ribis JW, Nieto C, DiBenedetto NV, Mehra A, Dong Q, Nagawa I, Meouche IE, Aldridge BB, Dunlop MJ, Tamayo R, Singh A, Shen A. 2024. Unique growth and morphology properties of Clade 5 Clostridioides difficile strains revealed by single-cell time-lapse microscopy. bioRxiv. doi:10.1101/2024.02.13.580212:2024.02.13.580212 PMC12140426 40397889

[70] Chandra H, Sorg JA, Hassett DJ, Sun X. 2023. Regulatory transcription factors of Clostridioides difficile pathogenesis with a focus on toxin regulation. Crit Rev Microbiol 49:334–349. doi:10.1080/1040841X.2022.2054307 35389761 PMC11209739

[71] Kempher ML, Morris SC, Shadid TM, Menon SK, Ballard JD, West AH. 2022. Response regulator CD1688 is a negative modulator of sporulation in Clostridioides difficile. J Bacteriol 204:e0013022. doi:10.1128/jb.00130-22 35852332 PMC9380558

[72] Johnstone MA, Self WT. 2022. D-proline reductase underlies proline-dependent growth of Clostridioides difficile. J Bacteriol 204:e0022922. doi:10.1128/jb.00229-22 35862761 PMC9380539

[73] Zhu D, Wang S, Sun X. 2021. FliW and CsrA Govern Flagellin (FliC) synthesis and play pleiotropic roles in virulence and physiology of Clostridioides difficile R20291. Front Microbiol 12:735616. doi:10.3389/fmicb.2021.735616 34675903 PMC8523840

[74] Nale JY, Al-Tayawi TS, Heaphy S, Clokie MRJ. 2021. Impact of phage CDHS-1 on the transcription, physiology and pathogenicity of a Clostridioides difficile ribotype 027 strain, R20291. Viruses 13:2262. doi:10.3390/v13112262 34835068 PMC8619979

[75] Edwards AN, Willams CL, Pareek N, McBride SM, Tamayo R. 2021. C-di-GMP inhibits early sporulation in Clostridioides difficile. mSphere 6:e0091921. doi:10.1128/msphere.00919-21 34878288 PMC8653836

[76] Pizarro-Guajardo M, Calderón-Romero P, Romero-Rodríguez A, Paredes-Sabja D. 2020. Characterization of exosporium layer variability of Clostridioides difficile spores in the epidemically relevant strain R20291. Front Microbiol 11:1345. doi:10.3389/fmicb.2020.01345 32714296 PMC7343902

[77] Bhattacharjee D, Sorg JA. 2020. Factors and conditions that impact electroporation of Clostridioides difficile strains. mSphere 5:e00941-19. doi:10.1128/mSphere.00941-19 PMC7056809 32132157

[78] Álvarez R, Ortega-Fuentes C, Queraltó C, Inostroza O, Díaz-Yáñez F, González R, Calderón IL, Fuentes JA, Paredes-Sabja D, Gil F. 2020. Evaluation of functionality of type II toxin-antitoxin systems of Clostridioides difficile R20291. Microbiol Res 239:126539. doi:10.1016/j.micres.2020.126539 32622285

[79] Shrestha R, Sorg JA. 2019. Terbium chloride influences Clostridium difficile spore germination. Anaerobe 58:80–88. doi:10.1016/j.anaerobe.2019.03.016 30926439 PMC6697597

[80] Pizarro-Guajardo M, Ravanal MC, Paez MD, Callegari E, Paredes-Sabja D. 2018. Identification of Clostridium difficile immunoreactive spore proteins of the epidemic strain R20291. Proteomics Clin Appl 12:e1700182. doi:10.1002/prca.201700182 29573213 PMC6370038

[81] Waslawski S, Lo ES, Ewing SA, Young VB, Aronoff DM, Sharp SE, Novak-Weekley SM, Crist AE, Dunne WM, Hoppe-Bauer J, Johnson M, Brecher SM, Newton DW, Walk ST. 2013. Clostridium difficile ribotype diversity at six health care institutions in the United States. J Clin Microbiol 51:1938–1941. doi:10.1128/JCM.00056-13 23554188 PMC3716112

[82] Imwattana K, Knight DR, Kullin B, Collins DA, Putsathit P, Kiratisin P, Riley TV. 2019. Clostridium difficile ribotype 017 - characterization, evolution and epidemiology of the dominant strain in Asia. Emerg Microbes Infect 8:796–807. doi:10.1080/22221751.2019.1621670 31138041 PMC6542179

[83] Shaw HA, Preston MD, Vendrik KEW, Cairns MD, Browne HP, Stabler RA, Crobach MJT, Corver J, Pituch H, Ingebretsen A, Pirmohamed M, Faulds-Pain A, Valiente E, Lawley TD, Fairweather NF, Kuijper EJ, Wren BW. 2020. The recent emergence of a highly related virulent Clostridium difficile clade with unique characteristics. Clin Microbiol Infect 26:492–498. doi:10.1016/j.cmi.2019.09.004 31525517 PMC7167513

[84] Prade RA. 1996. Xylanases: from biology to biotechnology. Biotechnol Genet Eng Rev 13:101–131. doi:10.1080/02648725.1996.10647925 8948110

[85] Moracci M, Cobucci Ponzano B, Trincone A, Fusco S, De Rosa M, van Der Oost J, Sensen CW, Charlebois RL, Rossi M. 2000. Identification and molecular characterization of the first alpha -xylosidase from an archaeon. J Biol Chem 275:22082–22089. doi:10.1074/jbc.M910392199 10801892

[86] George WL, Sutter VL, Citron D, Finegold SM. 1979. Selective and differential medium for isolation of Clostridium difficile. J Clin Microbiol 9:214–219. doi:10.1128/jcm.9.2.214-219.1979 429542 PMC272994

[87] Fishbein SR, Robinson JI, Hink T, Reske KA, Newcomer EP, Burnham C-A, Henderson JP, Dubberke ER, Dantas G. 2022. Multi-omics investigation of Clostridioides difficile-colonized patients reveals pathogen and commensal correlates of C. difficile pathogenesis. Elife 11:e72801. doi:10.7554/eLife.72801 35083969 PMC8794467

[88] Eyre DW, Didelot X, Buckley AM, Freeman J, Moura IB, Crook DW, Peto TEA, Walker AS, Wilcox MH, Dingle KE. 2019. Clostridium difficile trehalose metabolism variants are common and not associated with adverse patient outcomes when variably present in the same lineage. EBioMedicine 43:347–355. doi:10.1016/j.ebiom.2019.04.038 31036529 PMC6558026

[89] Marshall A, McGrath JW, Graham R, McMullan G. 2023. Food for thought-the link between Clostridioides difficile metabolism and pathogenesis. PLoS Pathog 19:e1011034. doi:10.1371/journal.ppat.1011034 36602960 PMC9815643

[90] Martinez-Augustin O, Sanchez de Medina F. 2008. Intestinal bile acid physiology and pathophysiology. World J Gastroenterol 14:5630–5640. doi:10.3748/wjg.14.5630 18837078 PMC2748196

[91] Hofmann AF. 1999. The continuing importance of bile acids in liver and intestinal disease. Arch Intern Med 159:2647–2658. doi:10.1001/archinte.159.22.2647 10597755

[92] Begley M, Gahan CGM, Hill C. 2005. The interaction between bacteria and bile. FEMS Microbiol Rev 29:625–651. doi:10.1016/j.femsre.2004.09.003 16102595

[93] Sorg JA, Sonenshein AL. 2008. Bile salts and glycine as cogerminants for Clostridium difficile spores. J Bacteriol 190:2505–2512. doi:10.1128/JB.01765-07 18245298 PMC2293200

[94] Francis MB, Allen CA, Shrestha R, Sorg JA. 2013. Bile acid recognition by the Clostridium difficile germinant receptor, CspC, is important for establishing infection. PLoS Pathog 9:e1003356. doi:10.1371/journal.ppat.1003356 23675301 PMC3649964

[95] Adams CM, Eckenroth BE, Putnam EE, Doublié S, Shen A. 2013. Structural and functional analysis of the CspB protease required for Clostridium spore germination. PLoS Pathog 9:e1003165. doi:10.1371/journal.ppat.1003165 23408892 PMC3567191

[96] Burns DA, Heap JT, Minton NP. 2010. SleC is essential for germination of Clostridium difficile spores in nutrient-rich medium supplemented with the bile salt taurocholate. J Bacteriol 192:657–664. doi:10.1128/JB.01209-09 19933358 PMC2812441

[97] Gutelius D, Hokeness K, Logan SM, Reid CW. 2014. Functional analysis of SleC from Clostridium difficile: an essential lytic transglycosylase involved in spore germination. Microbiology (Reading) 160:209–216. doi:10.1099/mic.0.072454-0 24140647 PMC3917228

[98] Rohlfing AE, Eckenroth BE, Forster ER, Kevorkian Y, Donnelly ML, Benito de la Puebla H, Doublié S, Shen A. 2019. The CspC pseudoprotease regulates germination of Clostridioides difficile spores in response to multiple environmental signals. PLoS Genet 15:e1008224. doi:10.1371/journal.pgen.1008224 31276487 PMC6636752

[99] Wilson KH. 1983. Efficiency of various bile salt preparations for stimulation of Clostridium difficile spore germination. J Clin Microbiol 18:1017–1019. doi:10.1128/jcm.18.4.1017-1019.1983 6630458 PMC270959

[100] Usui Y, Ayibieke A, Kamiichi Y, Okugawa S, Moriya K, Tohda S, Saito R. 2020. Impact of deoxycholate on Clostridioides difficile growth, toxin production, and sporulation. Heliyon 6:e03717. doi:10.1016/j.heliyon.2020.e03717 32322715 PMC7160582

[101] Payne AN, Zihler A, Chassard C, Lacroix C. 2012. Advances and perspectives in in vitro human gut fermentation modeling. Trends Biotechnol 30:17–25. doi:10.1016/j.tibtech.2011.06.011 21764163

[102] Procházková N, Falony G, Dragsted LO, Licht TR, Raes J, Roager HM. 2023. Advancing human gut microbiota research by considering gut transit time. Gut 72:180–191. doi:10.1136/gutjnl-2022-328166 36171079 PMC9763197

[103] Kettle H, Louis P, Flint HJ. 2022. Process-based modelling of microbial community dynamics in the human colon. J R Soc Interface 19:20220489. doi:10.1098/rsif.2022.0489

[104] Balaji A, Ozer EA, Kociolek LK. 2019. Clostridioides difficile whole-genome sequencing reveals limited within-host genetic diversity in a pediatric cohort. J Clin Microbiol 57:e00559-19. doi:10.1128/JCM.00559-19 31315950 PMC6711918

[105] Knight DR, Squire MM, Collins DA, Riley TV. 2016. Genome analysis of Clostridium difficile PCR ribotype 014 lineage in Australian pigs and humans reveals a diverse genetic repertoire and signatures of long-range interspecies transmission. Front Microbiol 7:2138. doi:10.3389/fmicb.2016.02138 28123380 PMC5225093

[106] Kociolek LK, Ozer EA, Gerding DN, Hecht DW, Patel SJ, Hauser AR. 2018. Whole-genome analysis reveals the evolution and transmission of an MDR DH/NAP11/106 Clostridium difficile clone in a paediatric hospital. J Antimicrob Chemother 73:1222–1229. doi:10.1093/jac/dkx523 29342270 PMC6454525

[107] Ramírez-Vargas G, Goh S, Rodríguez C. 2018. The novel phages phiCD5763 and phiCD2955 represent two groups of big plasmidial siphoviridae phages of Clostridium difficile. Front Microbiol 9:26. doi:10.3389/fmicb.2018.00026 29403466 PMC5786514

[108] Knetsch CW, Terveer EM, Lauber C, Gorbalenya AE, Harmanus C, Kuijper EJ, Corver J, van Leeuwen HC. 2012. Comparative analysis of an expanded Clostridium difficile reference strain collection reveals genetic diversity and evolution through six lineages. Infect Genet Evol 12:1577–1585. doi:10.1016/j.meegid.2012.06.003 22705462

[109] Li Z, Lee K, Rajyaguru U, Jones CH, Janezic S, Rupnik M, Anderson AS, Liberator P. 2020. Ribotype classification of Clostridioides difficile isolates is not predictive of the amino acid sequence diversity of the toxin virulence factors TcdA and TcdB. Front Microbiol 11:1310. doi:10.3389/fmicb.2020.01310 32636819 PMC7318873

[110] Martínez-Meléndez A, Morfin-Otero R, Villarreal-Treviño L, Baines SD, Camacho-Ortíz A, Garza-González E. 2020. Molecular epidemiology of predominant and emerging Clostridioides difficile ribotypes. J Microbiol Methods 175:105974. doi:10.1016/j.mimet.2020.105974 32531232

[111] Midani FS, Danhof HA, Mathew N, Ardis CK, Garey KW, Spinler JK, Britton RA. 2025. Emerging Clostridioides difficile ribotypes have divergent metabolic phenotypes. mSystems 10:e0107524. doi:10.1128/msystems.01075-24 40013784 PMC11915817

[112] Dong Q, Harper S, McSpadden E, Son SS, Allen MM, Lin H, Smith RC, Metcalfe C, Burgo V, Woodson C, Sundararajan A, Rose A, McMillin M, Moran D, Little J, Mullowney M, Sidebottom AM, Shen A, Fortier LC, Pamer EG. 2024. Protection against Clostridioides difficile disease by a naturally avirulent C. difficile strain. bioRxiv. doi:10.1101/2024.05.06.592814 PMC11731898 39610252

[113] Ahlman B, Leijonmarck CE, Lind C, Vinnars E, Wernerman J. 1993. Free amino acids in biopsy specimens from the human colonic mucosa. J Surg Res 55:647–653. doi:10.1006/jsre.1993.1198 8246499

[114] Ahlman B, Ljungqvist O, Persson B, Bindslev L, Wernerman J. 1995. Intestinal amino acid content in critically ill patients. JPEN J Parenter Enteral Nutr 19:272–278. doi:10.1177/0148607195019004272 8523625

[115] Alves A, Bassot A, Bulteau AL, Pirola L, Morio B. 2019. Glycine metabolism and its alterations in obesity and metabolic diseases. Nutrients 11:1356. doi:10.3390/nu11061356 31208147 PMC6627940

[116] Theriot CM, Koenigsknecht MJ, Carlson PE Jr, Hatton GE, Nelson AM, Li B, Huffnagle GB, Z Li J, Young VB. 2014. Antibiotic-induced shifts in the mouse gut microbiome and metabolome increase susceptibility to Clostridium difficile infection. Nat Commun 5:3114. doi:10.1038/ncomms4114 24445449 PMC3950275

[117] Wexler AG, Guiberson ER, Beavers WN, Shupe JA, Washington MK, Lacy DB, Caprioli RM, Spraggins JM, Skaar EP. 2021. Clostridioides difficile infection induces a rapid influx of bile acids into the gut during colonization of the host. Cell Rep 36:109683. doi:10.1016/j.celrep.2021.109683 34496241 PMC8445666

[118] Carr KA, Janes BK, Hanna PC. 2010. Role of the gerP operon in germination and outgrowth of Bacillus anthracis spores. PLoS One 5:e9128. doi:10.1371/journal.pone.0009128 20161744 PMC2817736

[119] Yip C, Phan JR, Abel-Santos E. 2023. Mechanism of germination inhibition of Clostridioides difficile spores by an aniline substituted cholate derivative (CaPA). J Antibiot (Tokyo) 76:335–345. doi:10.1038/s41429-023-00612-3 37016015 PMC10406169

[120] Yip C, Okada NC, Howerton A, Amei A, Abel-Santos E. 2021. Pharmacokinetics of CamSA, a potential prophylactic compound against Clostridioides difficile infections. Biochem Pharmacol 183:114314. doi:10.1016/j.bcp.2020.114314 33152344 PMC7770080

[121] Meléndez-Sánchez D, Hernández L, Ares M, Méndez Tenorio A, Flores-Luna L, Torres J, Camorlinga-Ponce M. 2024. Genomic and phenotypic studies among Clostridioides difficile isolates show a high prevalence of clade 2 and great diversity in clinical isolates from Mexican adults and children with healthcare-associated diarrhea. Microbiol Spectr 12:e0394723. doi:10.1128/spectrum.03947-23 38864670 PMC11218462

[122] Muñoz M, Restrepo-Montoya D, Kumar N, Iraola G, Camargo M, Díaz-Arévalo D, Roa-Molina NS, Tellez MA, Herrera G, Ríos-Chaparro DI, Birchenall C, Pinilla D, Pardo-Oviedo JM, Rodríguez-Leguizamón G, Josa DF, Lawley TD, Patarroyo MA, Ramírez JD. 2019. Integrated genomic epidemiology and phenotypic profiling of Clostridium difficile across intra-hospital and community populations in Colombia. Sci Rep 9:11293. doi:10.1038/s41598-019-47688-2 31383872 PMC6683185

[123] Dingle KE, Elliott B, Robinson E, Griffiths D, Eyre DW, Stoesser N, Vaughan A, Golubchik T, Fawley WN, Wilcox MH, Peto TE, Walker AS, Riley TV, Crook DW, Didelot X. 2014. Evolutionary history of the Clostridium difficile pathogenicity locus. Genome Biol Evol 6:36–52. doi:10.1093/gbe/evt204 24336451 PMC3914685

[124] Drudy D, Fanning S, Kyne L. 2007. Toxin A-negative, toxin B-positive Clostridium difficile. Int J Infect Dis 11:5–10. doi:10.1016/j.ijid.2006.04.003 16857405

[125] Hamilton JP, Xie G, Raufman J-P, Hogan S, Griffin TL, Packard CA, Chatfield DA, Hagey LR, Steinbach JH, Hofmann AF. 2007. Human cecal bile acids: concentration and spectrum. Am J Physiol Gastrointest Liver Physiol 293:G256–G263. doi:10.1152/ajpgi.00027.2007 17412828

[126] Monteford J, Bilverstone TW, Ingle P, Philip S, Kuehne SA, Minton NP. 2021. What’s a SNP between friends: the lineage of Clostridioides difficile R20291 can effect research outcomes. Anaerobe 71:102422. doi:10.1016/j.anaerobe.2021.102422 34343672 PMC8556159

[127] Riedel T, Wetzel D, Hofmann JD, Plorin SPEO, Dannheim H, Berges M, Zimmermann O, Bunk B, Schober I, Spröer C, Liesegang H, Jahn D, Overmann J, Groß U, Neumann-Schaal M. 2017. High metabolic versatility of different toxigenic and non-toxigenic Clostridioides difficile isolates. Int J Med Microbiol 307:311–320. doi:10.1016/j.ijmm.2017.05.007 28619474

[128] Norsigian CJ, Danhof HA, Brand CK, Oezguen N, Midani FS, Palsson BO, Savidge TC, Britton RA, Spinler JK, Monk JM. 2020. Systems biology analysis of the Clostridioides difficile core-genome contextualizes microenvironmental evolutionary pressures leading to genotypic and phenotypic divergence. NPJ Syst Biol Appl 6:31. doi:10.1038/s41540-020-00151-9 33082337 PMC7576604

[129] Lewis BB, Carter RA, Ling L, Leiner I, Taur Y, Kamboj M, Dubberke ER, Xavier J, Pamer EG. 2017. Pathogenicity locus, core genome, and accessory gene contributions to Clostridium difficile virulence. MBio 8:e00885-17. doi:10.1128/mBio.00885-17 28790208 PMC5550754

[130] Joshi LT, Phillips DS, Williams CF, Alyousef A, Baillie L. 2012. Contribution of spores to the ability of Clostridium difficile to adhere to surfaces. Appl Environ Microbiol 78:7671–7679. doi:10.1128/AEM.01862-12 22923404 PMC3485709

[131] Merrigan MM, Venugopal A, Roxas JL, Anwar F, Mallozzi MJ, Roxas BAP, Gerding DN, Viswanathan VK, Vedantam G. 2013. Surface-layer protein A (SlpA) is a major contributor to host-cell adherence of Clostridium difficile. PLoS One 8:e78404. doi:10.1371/journal.pone.0078404 24265687 PMC3827033

[132] Saund K, Pirani A, Lacy DB, Hanna PC, Snitkin E. 2022. Strain variation in Clostridioides difficile cytotoxicity associated with genomic variation at both pathogenic and nonpathogenic loci. mSphere 7:e0017422. doi:10.1128/msphere.00174-22 35766503 PMC9241522

