# Supplementary material for: Phenotypic analysis of various Clostridioides difficile ribotypes reveals consistency among core processes
Source: Appl Environ Microbiol. 2025 Jun 24;91(7):e00964-25. doi: 10.1128/aem.00964-25 (PMC12285255; doi:10.1128/aem.00964-25)
Supplement: Supplemental figures, part I — Figures S1 to S8. [file aem.00964-25-s0001.pdf]

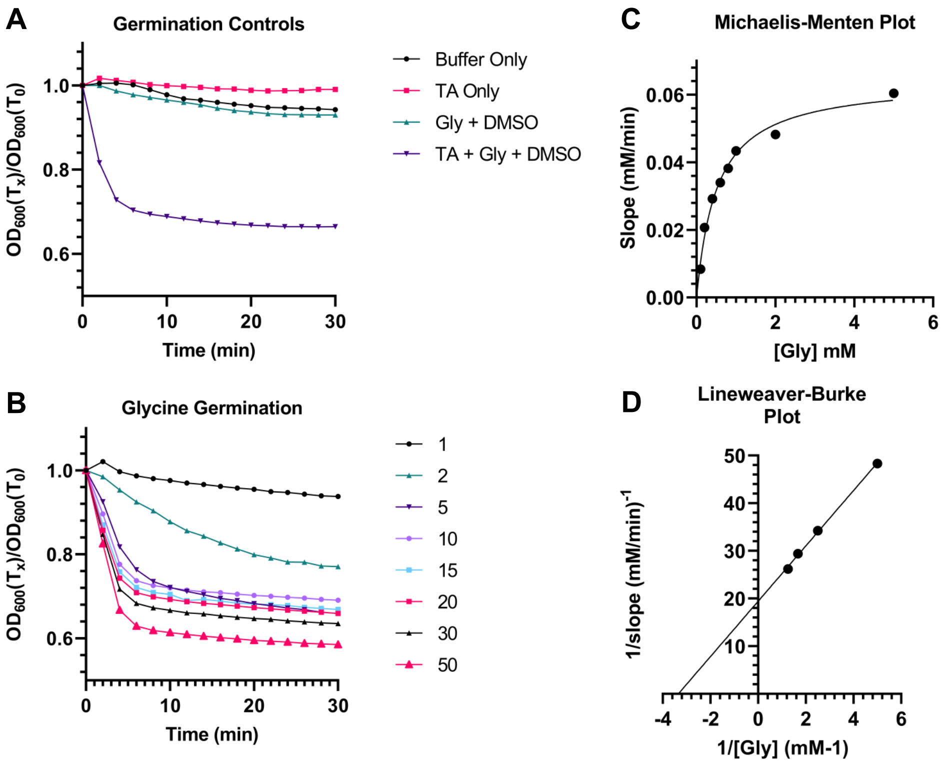
A
Germination Controls
Buffer Only
TA Only
Gly + DMSO
TA + Gly + DMSO
OD600(Tx)/OD600(T0)
Time (min)
B
Glycine Germination
1
2
5
10
15
20
30
50
OD600(Tx)/OD600(T0)
Time (min)
C
Michaelis-Menten Plot
Slope (mM/min)
[Gly] mM
D
Lineweaver-Burke Plot
1/slope (mM/min)-1
1/[Gly] (mM-1)

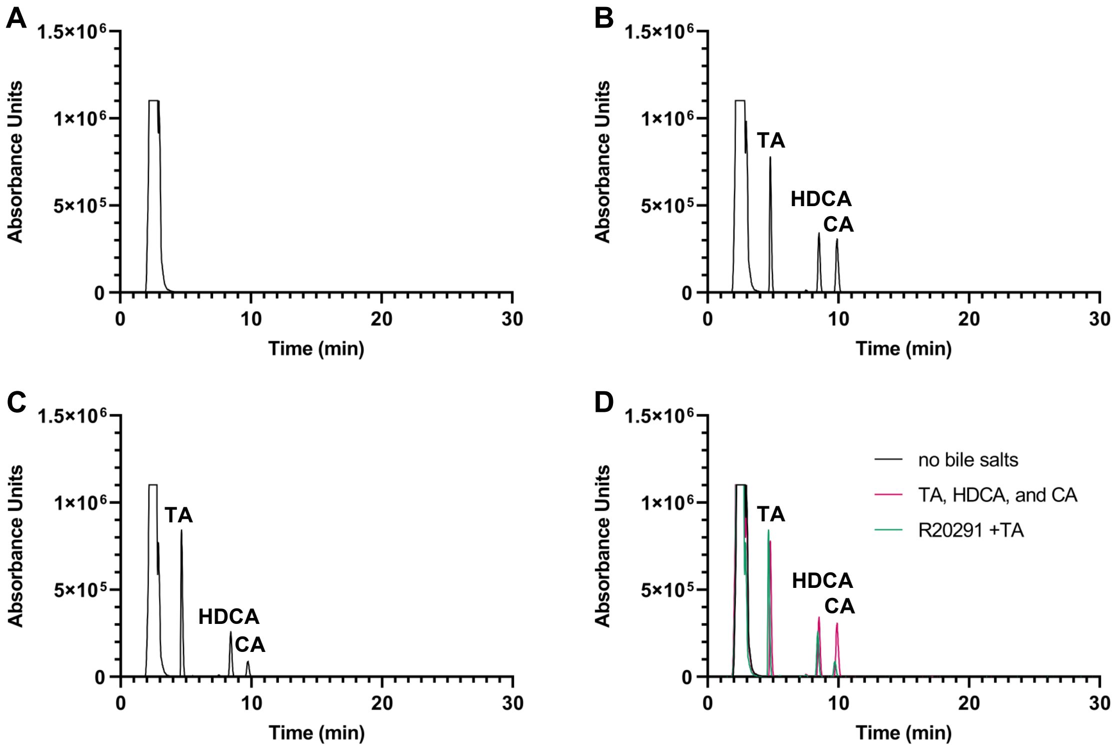

A
B
C
D
1.5×10^6
1×10^6
5×10^5
0
Absorbance Units
Time (min)
0
10
20
30
TA
HDCA
CA
no bile salts
TA, HDCA, and CA
R20291 +TA

A

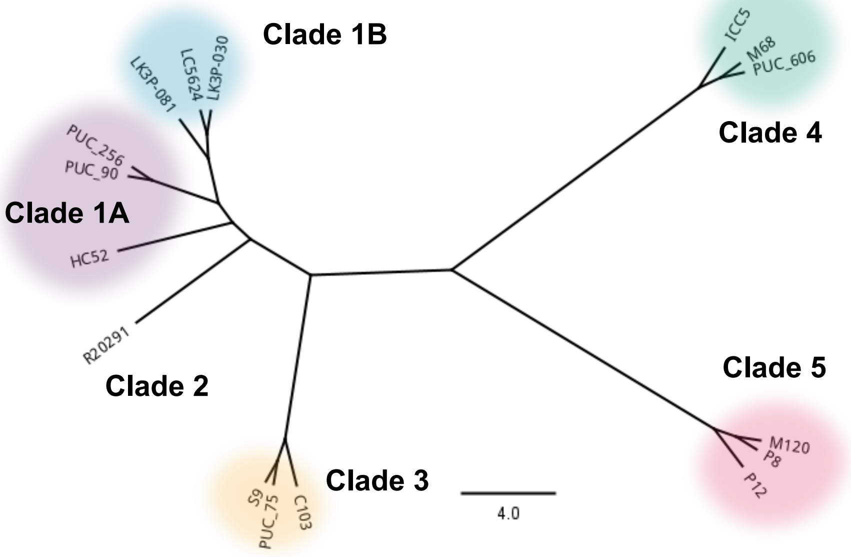
Clade 1B
LK3P-081
LC5624
LK3P-030
PUC_256
PUC_90
Clade 1A
HC52
R20291
Clade 2
S9
PUC_75
C103
Clade 3
4.0
ICC5
M68
PUC_606
Clade 4
Clade 5
M120
P8
P12

B

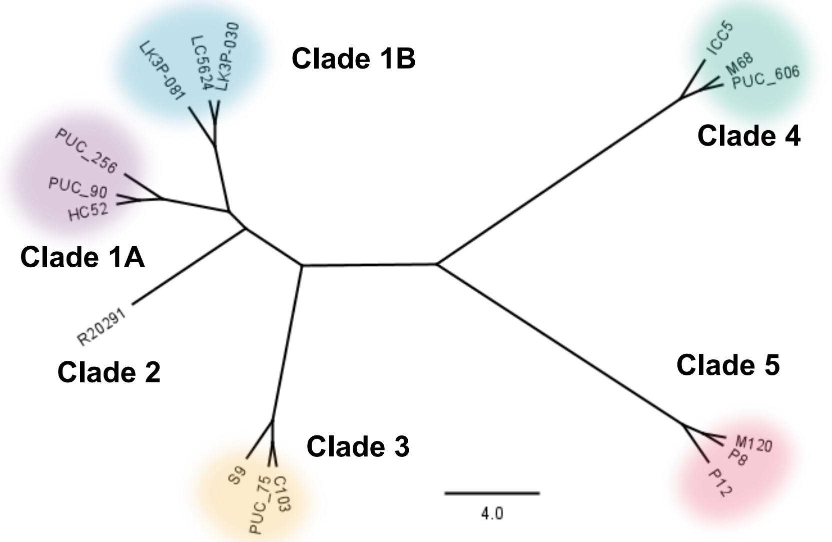
LK3P-081
LC5624
LK3P-030
Clade 1B
PUC_256
PUC_90
HC52
Clade 1A
R20291
Clade 2
S9
PUC_75
C103
Clade 3
4.0
ICC5
M68
PUC_606
Clade 4
Clade 5
M120
P8
P12

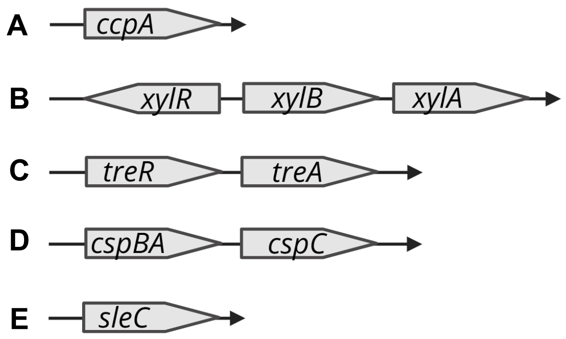
A
ccpA
B
xylR
xylB
xylA
C
treR
treA
D
cspBA
cspC
E
sleC

CcpA

```
          1       10        20        30        40        50        60        70        80        90
R20291    MKGNITIKDVAKQAGVSISTVSRVINDSKPVTDEVKQKVLEVIKETGYIPNPLARSLVTKKSQLIGVIVPEVSDSFVNEVLNGIEEVAKMYDYD

HC52      MKGNITIKDVAKQAGVSISTVSRVINDSKPVTDEVKQKVLEVIKETGYIPNPLARSLVTKKSQLIGVIVPEVSDSFVNEVLNGIEEVAKMYDYD
PUC_256   MKGNITIKDVAKQAGVSISTVSRVINDSKPVTDEVKQKVLEVIKETGYIPNPLARSLVTKKSQLIGVIVPEVSDSFVNEVLNGIEEVAKMYDYD
PUC_90    MKGNITIKDVAKQAGVSISTVSRVINDSKPVTDEVKQKVLEVIKETGYIPNPLARSLVTKKSQLIGVIVPEVSDSFVNEVLNGIEEVAKMYDYD
LC5624    MKGNITIKDVAKQAGVSISTVSRVINDSKPVTDEVKQKVLEVIKETGYIPNPLARSLVTKKSQLIGVIVPEVSDSFVNEVLNGIEEVAKMYDYD
LK3P-030  MKGNITIKDVAKQAGVSISTVSRVINDSKPVTDEVKQKVLEVIKETGYIPNPLARSLVTKKSQLIGVIVPEVSDSFVNEVLNGIEEVAKMYDYD
PUC_75    MKGNITIKDVAKQAGVSISTVSRVINDSKPVTDEVKQKVLEVIKETGYIPNPLARSLVTKKSQLIGVIVPEVSDSFVNEVLNGIEEVAKMYDYD
LK3P-081  MKGNITIKDVAKQAGVSISTVSRVINDSKPVTDEVKQKVLEVIKETGYIPNPLARSLVTKKSQLIGVIVPEVSDSFVNEVLNGIEEVAKMYDYD
C103      MKGNITIKDVAKQAGVSISTVSRVINDSKPVTDEVKQKVLEVIKETGYIPNPLARSLVTKKSQLIGVIVPEVSDSFVNEVLNGIEEVAKMYDYD
S9        MKGNITIKDVAKQAGVSISTVSRVINDSKPVTDEVKQKVLEVIKETGYIPNPLARSLVTKKSQLIGVIVPEVSDSFVNEVLNGIEEVAKMYDYD
M68       MKGNITIKDVAKQAGVSISTVSRVINDSKPVTDEVKQKVLEVIKETGYIPNPLARSLVTKKSQLIGVIVPEVSDSFVNEVLNGIEEVAKMYDYD
PUC_606   MKGNITIKDVAKQAGVSISTVSRVINDSKPVTDEVKQKVLEVIKETGYIPNPLARSLVTKKSQLIGVIVPEVSDSFVNEVLNGIEEVAKMYDYD
ICC5      MKGNITIKDVAKQAGVSISTVSRVINDSKPVTDEVKQKVLEVIKETGYIPNPLARSLVTKKSQLIGVIVPEVSDSFVNEVLNGIEEVAKMYDYD
M120      MKGNITIKDVAKQAGVSISTVSRVINDSKPVTDEVKQKVLEVIKETGYIPNPLARSLVTKKSQLIGVIVPEVSDSFVNEVLNGIEEVAKMYDYD
P8        MKGNITIKDVAKQAGVSISTVSRVINDSKPVTDEVKQKVLEVIKETGYIPNPLARSLVTKKSQLIGVIVPEVSDSFVNEVLNGIEEVAKMYDYD
P12       MKGNITIKDVAKQAGVSISTVSRVINDSKPVTDEVKQKVLEVIKETGYIPNPLARSLVTKKSQLIGVIVPEVSDSFVNEVLNGIEEVAKMYDYD
```

```
              100       110       120       130       140       150       160       170       180
R20291    ILLANTYSDKEQELKSINLLRAKQVEGIVMISWIVEQEHINYIQNCGIPATYISKTARNYDIYTVSTSNEEATFDMTEHLIKKGHEKIAFIMTS

HC52      ILLANTYSDKEQELKSINLLRAKQVEGIVMISWIVEQEHINYIQNCGIPATYISKTARNYDIYTVSTSNEEATFDMTEHLIKKGHEKIAFIMTS
PUC_256   ILLANTYSDKEQELKSINLLRAKQVEGIVMISWIVEQEHINYIQNCGIPATYISKTARNYDIYTVSTSNEEATFDMTEHLIKKGHEKIAFIMTS
PUC_90    ILLANTYSDKEQELKSINLLRAKQVEGIVMISWIVEQEHINYIQNCGIPATYISKTARNYDIYTVSTSNEEATFDMTEHLIKKGHEKIAFIMTS
LC5624    ILLANTYSDKEQELKSINLLRAKQVEGIVMISWIVEQEHINYIQNCGIPATYISKTARNYDIYTVSTSNEEATFDMTEHLIKKGHEKIAFIMTS
LK3P-030  ILLANTYSDKEQELKSINLLRAKQVEGIVMISWIVEQEHINYIQNCGIPATYISKTARNYDIYTVSTSNEEATFDMTEHLIKKGHEKIAFIMTS
PUC_75    ILLANTYSDKEQELKSINLLRAKQVEGIVMISWIVEQEHINYIQNCGIPATYISKTARNYDIYTVSTSNEEATFDMTEXLIKKGHEKIAFIMTS
LK3P-081  ILLANTYSDKEQELKSINLLRAKQVEGIVMISWIVEQEHINYIQNCGIPATYISKTARNYDIYTVSTSNEEATFDMTEHLIKKGHEKIAFIMTS
C103      ILLANTYSDKEQELKSINLLRAKQVEGIVMISWIVEQEHINYIQNCGIPATYISKTARNYDIYTVSTSNEEATFDMTEHLIKKGHEKIAFIMTS
S9        ILLANTYSDKEQELKSINLLRAKQVEGIVMISWIVEQEHINYIQNCGIPATYISKTARNYDIYTVSTSNEEATFDMTEHLIKKGHEKIAFIMTS
M68       ILLANTYSDKEQELKSINLLRAKQVEGIVMISWIVEQEHINYIQNCGIPATYISKTARNYDIYTVSTSNEEATFDMTEHLIKKGHEKIAFIMTS
PUC_606   ILLANTYSDKEQELKSINLLRAKQVEGIVMISWIVEQEHINYIQNCGIPATYISKTARNYDIYTVSTSNEEATFDMTEHLIKKGHEKIAFIMTS
ICC5      ILLANTYSDKEQELKSINLLRAKQVEGIVMISWIVEQEHINYIQNCGIPATYISKTARNYDIYTVSTSNEEATFDMTEHLIKKGHEKIAFIMTS
M120      ILLANTYSDKEQELKSINLLRAKQVEGIVMISWIVEQEHINYIQNCGIPATYISKTARNYDIYTVSTSNEEATFDMTEHLIKKGHEKIAFIMTS
P8        ILLANTYSDKEQELKSINLLRAKQVEGIVMISWIVEQEHINYIQNCGIPATYISKTARNYDIYTVSTSNEEATFDMTEHLIKKGHEKIAFIMTS
P12       ILLANTYSDKEQELKSINLLRAKQVEGIVMISWIVEQEHINYIQNCGIPATYISKTARNYDIYTVSTSNEEATFDMTEHLIKKGHEKIAFIMTS
```

```
           190       200       210       220       230       240       250       260       270       280
R20291    KDDTVLEMERLAGYEKALSNNNIELDKSLIKYGGTDYESGYNSMKELLDDGIIPHAAFVTGDEAAIGAINAICDAGYKVPEDISVAGFNDVKIA

HC52      KDDTVLEMERLAGYEKALSNNNIELDKSLIKYGGTDYESGYNSMKELLDDGIIPHAAFVTGDEAAIGAINAICDAGYKVPEDISVAGFNDVKIA
PUC_256   KDDTVLEMERLAGYEKALSNNNIELDKSLIKYGGTDYESGYNSMKELLDDGIIPHAAFVTGDEAAIGAINAICDAGYKVPEDISVAGFNDVKIA
PUC_90    KDDTVLEMERLAGYEKALSNNNIELDKSLIKYGGTDYESGYNSMKELLDDGIIPHAAFVTGDEAAIGAINAICDAGYKVPEDISVAGFNDVKIA
LC5624    KDDTVLEMERLAGYEKALSNNNIELDKSLIKYGGTDYESGYNSMKELLDDGIIPHAAFVTGDEAAIGAINAICDAGYKVPEDISVAGFNDVKIA
LK3P-030  KDDTVLEMERLAGYEKALSNNNIELDKSLIKYGGTDYESGYNSMKELLDDGIIPHAAFVTGDEAAIGAINAICDAGYKVPEDISVAGFNDVKIA
PUC_75    KDDTVLEMERLAGYEKALSNNNIELDKSLIKYGGTDYESGYNSMKELLDDGIIPHAAFVTGDEAAIGAINAICDAGYKVPEDISVAGFNDVKIA
LK3P-081  KDDTVLEMERLAGYEKALSNNNIELDKSLIKYGGTDYESGYNSMKELLDDGIIPHAAFVTGDEAAIGAINAICDAGYKVPEDISVAGFNDVKIA
C103      KDDTVLEMERLAGYEKALSNNNIELDKSLIKYGGTDYESGYNSMKELLDDGIIPHAAFVTGDEAAIGAINAICDAGYKVPEDISVAGFNDVKIA
S9        KDDTVLEMERLAGYEKALSNNNIELDKSLIKYGGTDYESGYNSMKELLDDGIIPHAAFVTGDEAAIGAINAICDAGYKVPEDISVAGFNDVKIA
M68       KDDTVLEMERLAGYEKALSNNNIELDKSLIKYGGTDYESGYNSMKELLDDGIIPHAAFVTGDEAAIGAINAICDAGYKVPEDISVAGFNDVKIA
PUC_606   KDDTVLEMERLAGYEKALSNNNIELDKSLIKYGGTDYESGYNSMKELLDDGIIPHAAFVTGDEAAIGAINAICDAGYKVPEDISVAGFNDVKIA
ICC5      KDDTVLEMERLAGYEKALSNNNIELDKSLIKYGGTDYESGYNSMKELLDDGIIPHAAFVTGDEAAIGAINAICDAGYKVPEDISVAGFNDVKIA
M120      KDDTVLEMERLAGYEKALSNNNIELDKSLIKYGGTDYESGYNSMKELLDDGIIPHAAFVTGDEAAIGAINAICDAGYKVPEDISVSGFNDVKIA
P8        KDDTVLEMERLAGYEKALSNNNIELDKSLIKYGGTDYESGYNSMKELLDDGIIPHAAFVTGDEAAIGAINAICDAGYKVPEDISVSGFNDVKIA
P12       KDDTVLEMERLAGYEKALSNNNIELDKSLIKYGGTDYESGYNSMKELLDDGIIPHAAFVTGDEAAIGAINAICDAGYKVPEDISVSGFNDVKIA
```

```
                 290       300       310       320         338
R20291    RMYRPKLTTVYQPLYDMGAVAIRMVIKLINKELIENKKIELPYRIVDRESVTERKK

HC52      RMYRPKLTTVYQPLYDMGAVAIRMVIKLINKELIENKKIELPYRIVDRESVTERKK
PUC_256   RMYRPKLTTVYQPLYDMGAVAIRMVIKLINKELIENKKIELPYRIVDRESVTERKK
PUC_90    RMYRPKLTTVYQPLYDMGAVAIRMVIKLINKELIENKKIELPYRIVDRESVTERKK
LC5624    RMYRPKLTTVYQPLYDMGAVAIRMVIKLINKELIENKKIELPYRIVDRESVTERKK
LK3P-030  RMYRPKLTTVYQPLYDMGAVAIRMVIKLINKELIENKKIELPYRIVDRESVTERKK
PUC_75    RMYRPKLTTVYQPLYDMGAVAIRMVIKLINKELIENKKIELPYRIVDRESVTERKK
LK3P-081  RMYRPKLTTVYQPLYDMGAVAIRMVIKLINKELIENKKIELPYRIVDRESVTERKK
C103      RMYRPKLTTVYQPLYDMGAVAIRMVIKLINKELIENKKIELPYRIVDRESVTERKK
S9        RMYRPKLTTVYQPLYDMGAVAIRMVIKLINKELIENKKIELPYRIVDRESVTERKK
M68       RMYRPKLTTVYQPLYDMGAVAIRMVIKLINKELIENKKIELPYRIVDRESVTERKK
PUC_606   RMYRPKLTTVYQPLYDMGAVAIRMVIKLINKELIENKKIELPYRIVDRESVTERKK
ICC5      RMYRPKLTTVYQPLYDMGAVAIRMVIKLINKELIENKKIELPYRIVDRESVTERKK
M120      KMYRPKLTTVYQPLYDMGAVAIRMVIKLINKELIENKKIELPYRIVDRESVIERKK
P8        KMYRPKLTTVYQPLYDMGAVAIRMVIKLINKELIENKKIELPYRIVDRESVIERKK
P12       KMYRPKLTTVYQPLYDMGAVAIRMVIKLINKELIENKKIELPYRIVDRESVIERKK
```

XylA

```
           1        10        20        30        40        50        60        70        80        90
R20291     MSEIFKGIGQIKFEGVKSDNELAFRYYNPEQVVGNKTMKEHLRFAMSYWHTLCGEGNDPFGVGTVERPWNNVTDPIEIAKIKVDAGFEFMSKMG
PUC_256    MSEIFKGIGQIKFEGVKSDNELAFRYYNPEQVVGNKTMKEHLRFAMSYWHTLCGEGNDPFGVGTVERPWNNITDPIEIAKIKVDAGFEFMSKMG
HC52       MSEIFKGIGQIKFEGVKSDNELAFRYYNPEQVVGNKTMKEHLRFAMSYWHTLCGEGNDPFGVGTVERPWNNITDPIEIAKIKVDAGFEFMSKMG
PUC_90     MSEIFKGIGQIKFEGVKSDNELAFRYYNPEQVVGNKTMKEHLRFAMSYWHTLCGEGNDPFGVGTVERPWNNITDPIEIAKIKVDAGFEFMSKMG
LC5624     MSEIFKGIGQIKFEGVKSDNELAFRYYNPEQVVGNKTMKEHLRFAMSYWHTLCGEGNDPFGVGTVERPWNNITDPIEIAKIKVDAGFEFMSKMG
LK3P-030   MSEIFKGIGQIKFEGVKSDNELAFRYYNPEQVVGNKTMKEHLRFAMSYWHTLCGEGNDPFGVGTVERPWNNITDPIEIAKIKVDAGFEFMSKMG
LK3P-081   MSEIFKGIGQIKFEGVKSDNELAFRYYNPEQVVGNKTMKEHLRFAMSYWHTLCGEGNDPFGVGTVERPWNNITDPIEIAKIKVDAGFEFMSKMG
PUC_75     MSEIFKGIGQIKFEGVKSDNELAFRYYNPEQVVGNKTMKEHLRFAMSYWHTLCGEGNDPFGVGTVERPWNNITDPIEIAKIKVDAGFEFMSKMG
S9         MSEIFKGIGQIKFEGVKSDNELAFRYYNPEQVVGNKTMKEHLRFAMSYWHTLCGEGNDPFGVGTVERPWNNITDPIEIAKIKVDAGFEFMSKMG
C103       MSEIFKGIGQIKFEGVKSDNELAFRYYNPEQVVGNKTMKEHLRFAMSYWHTLCGEGNDPFGVGTVERPWNNITDPIEIAKIKVDAGFEFMSKMG
M68        MSEIFKGIGQIKFEGVKSDNELAFRYYNPEQVVGNKTMKEHLRFAMSYWHTLCGEGNDPFGVGTVERPWNNITDPIEIAKIKVDAGFEFMSKMG
PUC_606    MSEIFKGIGQIKFEGVKSDNELAFRYYNPEQVVGNKTMKEHLRFAMSYWHTLCGEGNDPFGVGTVERPWNNITDPIEIAKIKVDAGFEFMSKMG
ICC5       MSEIFKGIGQIKFEGVKSDNELAFRYYNPEQVVGNKTMKEHLRFAMSYWHTLCGEGNDPFGVGTVERPWNNITDPIEIAKIKVDAGFEFMSKMG
M120       MNEIFKGIGQIKFEGVKSNNELAFRYYNPEQVVGNKTMKEHLRFAMSYWHTLCGEGNDPFGVGTVERPWNNITDPIEIAKIKVDAGFEFMSKMG
P8         MNEIFKGIGQIKFEGVKSNNELAFRYYNPEQVVGNKTMKEHLRFAMSYWHTLCGEGNDPFGVGTVERPWNNITDPIEIAKIKVDAGFEFMSKMG
P12        MNEIFKGIGQIKFEGVKSNNELAFRYYNPEQVVGNKTMKEHLRFAMSYWHTLCGEGNDPFGVGTVERPWNNITDPIEIAKIKVDAGFEFMSKMG

                100       110       120       130       140       150       160       170       180
R20291     IEYFCFHDRDIAPEGRDLEETNKILDEIVEYIKVNMEKTGIKLLWGTANMFGNPRFVHGASTTCNADVYAYAAAQVKKAMEITKYLGGENFVFW
PUC_256    IEYFCFHDRDIAPEGRDLEETNKILDEIVEYIKVNMEKTGIKLLWGTANMFGNPRFVHGASTTCNADVYAYAAAQVKKAMEITKYLGGENFVFW
HC52       IEYFCFHDRDIAPEGRDLEETNKILDEIVEYIKVNMEKTGIKLLWGTANMFGNPRFVHGASTTCNADVYAYAAAQVKKAMEITKYLGGENFVFW
PUC_90     IEYFCFHDRDIAPEGRDLEETNKILDEIVEYIKVNMEKTGIKLLWGTANMFGNPRFVHGASTTCNADVYAYAAAQVKKAMEITKYLGGENFVFW
LC5624     IEYFCFHDRDIAPEGRDLEETNKILDEIVEYIKVNMEKTGIKLLWGTANMFGNPRFVHGASTTCNADVYAYAAAQVKKAMEITKYLGGENFVFW
LK3P-030   IEYFCFHDRDIAPEGRDLEETNKILDEIVEYIKVNMEKTGIKLLWGTANMFGNPRFVHGASTTCNADVYAYAAAQVKKAMEITKYLGGENFVFW
LK3P-081   IEYFCFHDRDIAPEGRDLEETNKILDEIVEYIKVNMEKTGIKLLWGTANMFGNPRFVHGASTTCNADVYAYAAAQVKKAMEITKYLGGENFVFW
PUC_75     IEYFCFHDRDIAPEGRDLEETNKILDEIVEYIKANMEKTGIKLLWGTANMFGNPRFVHGASTTCNADVYAYAAAQVKKAMEITKYLGGENFVFW
S9         IEYFCFHDRDIAPEGRDLEETNKILDEIVEYIKANMEKTGIKLLWGTANMFGNPRFVHGASTTCNADVYAYAAAQVKKAMEITKYLGGENFVFW
C103       IEYFCFHDRDIAPEGRDLEETNKILDEIVEYIKANMEKTGIKLLWGTANMFGNPRFVHGASTTCNADVYAYAAAQVKKAMEITKYLGGENFVFW
M68        IEYFCFHDRDIAPEGRDLEETNKILDEIVEYIKANMEKTGIKLLWGTANMFGNPRFVHGASTTCNADVYAYAAAQVKKAMEITKYLGGENFVFW
PUC_606    IEYFCFHDRDIAPEGRDLEETNKILDEIVEYIKANMEKTGIKLLWGTANMFGNPRFVHGASTTCNADVYAYAAAQVKKAMEITKYLGGENFVFW
ICC5       IEYFCFHDRDIAPEGRDLEETNKILDEIVEYIKANMEKTGIKLLWGTANMFGNPRFVHGASTTCNADVYAYAAAQVKKAMEITKYLGGENFVFW
M120       IEYFCFHDRDIAPEGRDLEETNKILDEIVEYIKANMEKTGIKLLWGTANMFGNPRFVHGASTTCNADVYAYAAAQVKKAMEITKYLGGENFVFW
P8         IEYFCFHDRDIAPEGRDLEETNKILDEIVEYIKANMEKTGIKLLWGTANMFGNPRFVHGASTTCNADVYAYAAAQVKKAMEITKYLGGENFVFW
P12        IEYFCFHDRDIAPEGRDLEETNKILDEIVEYIKANMEKTGIKLLWGTANMFGNPRFVHGASTTCNADVYAYAAAQVKKAMEITKYLGGENFVFW

            190       200       210       220       230       240       250       260       270       280
R20291     GGREGYETLLNTNTELEMDNFARFLQMAVDYAKEIGFTGQFLIEPKPKEPTKHQYDFDTATVLGFLRKYNLDKYFKMNIEANHATLAGHTFQHE
PUC_256    GGREGYETLLNTNTELEMDNFARFLQMAVDYAKEIGFTGQFLIEPKPKEPTKHQYDFDTATVLGFLRKYNLDKYFKMNIEANHATLAGHTFQHE
HC52       GGREGYETLLNTNTELEMDNFARFLQMAVDYAKEIGFTGQFLIEPKPKEPTKHQYDFDTATVLGFLRKYNLDKYFKMNIEANHATLAGHTFQHE
PUC_90     GGREGYETLLNTNTELEMDNFARFLQMAVDYAKEIGFTGQFLIEPKPKEPTKHQYDFDTATVLGFLRKYNLDKYFKMNIEANHATLAGHTFQHE
LC5624     GGREGYETLLNTNTELEMDNFARFLQMAVDYAKEIGFSGQFLIEPKPKEPTKHQYDFDTATVLGFLRKYNLDKYFKMNIEANHATLAGHTFQHE
LK3P-030   GGREGYETLLNTNTELEMDNFARFLQMAVDYAKEIGFSGQFLIEPKPKEPTKHQYDFDTATVLGFLRKYNLDKYFKMNIEANHATLAGHTFQHE
LK3P-081   GGREGYETLLNTNTELEMDNFARFLQMAVDYAKEIGFTGQFLIEPKPKEPTKHQYDFDTATVLGFLRKYNLDKYFKMNIEANHATLAGHTFQHE
PUC_75     GGREGYETLLNTNTELEMDNFARFLQMAVDYAKEIGFTGQFLIEPKPKEPTKHQYDFDTATVLGFLRKYNLDKYFKVNIEANHATLAGHTFQHE
S9         GGREGYETLLNTNTELEMDNFARFLQMAVDYAKEIGFTGQFLIEPKPKEPTKHQYDFDTATVLGFLRKYNLDKYFKVNIEANHATLAGHTFQHE
C103       GGREGYETLLNTNTELEMDNFARFLQMAVDYAKEIGFTGQFLIEPKPKEPTKHQYDFDTATVLGFLRKYNLDKYFKVNIEANHATLAGHTFQHE
M68        GGREGYETLLNTNTELEMDNFARFLQMAVDYAKEIGFTGQFLIEPKPKEPTKHQYDFDTATVLGFLRKYNLDKYFKVNIEANHATLAGHTFQHE
PUC_606    GGREGYETLLNTNTELEMDNFARFLQMAVDYAKEIGFTGQFLIEPKPKEPTKHQYDFDTATVLGFLRKYNLDKYFKVNIEANHATLAGHTFQHE
ICC5       GGREGYETLLNTNTELEMDNFARFLQMAVDYAKEIGFTGQFLIEPKPKEPTKHQYDFDTATVLGFLRKYNLDKYFKVNIEANHATLAGHTFQHE
M120       GGREGYETLLNTNTELEMDNFARFLQMAVDYAKEIGFTGQFLIEPKPKEPTKHQYDFDTATVLGFLRKYNLDKYFKVNIEANHATLAGHTFQHE
P8         GGREGYETLLNTNTELEMDNFARFLQMAVDYAKEIGFTGQFLIEPKPKEPTKHQYDFDTATVLGFLRKYNLDKYFKVNIEANHATLAGHTFQHE
P12        GGREGYETLLNTNTELEMDNFARFLQMAVDYAKEIGFTGQFLIEPKPKEPTKHQYDFDTATVLGFLRKYNLDKYFKVNIEANHATLAGHTFQHE

                  290       300       310       320       330       340       350       360       370
R20291     LNIARINNVLGSIDANQGDLLLGWDTDQFPTNIYDATLAMYEVLKQGGIAPGGFNFDSKVRRASFEVEDLFLAYIAGMDTFAKGLLIAHKLLED
PUC_256    LNIARINNVLGSIDANQGDLLLGWDTDQFPTNIYDATLAMYEVLKQGGIAPGGFNFDSKVRRASFEVEDLFLAYIAGMDTFAKGLLIAHKLLED
HC52       LNIARINNVLGSIDANQGDLLLGWDTDQFPTNIYDATLAMYEVLKQGGIAPGGFNFDSKVRRASFEVEDLFLAYIAGMDTFAKGLLIAHKLLED
PUC_90     LNIARINNVLGSIDANQGDLLLGWDTDQFPTNIYDATLAMYEVLKQGGIAPGGFNFDSKVRRASFEVEDLFLAYIAGMDTFAKGLLIAHKLLED
LC5624     LNIARINNVLGSIDANQGDLLLGWDTDQFPTNIYDATLAMYEVLKQGGIAPGGFNFDSKVRRASFEVEDLFLAYIAGMDTFAKGLLIAHKLLED
LK3P-030   LNIARINNVLGSIDANQGDLLLGWDTDQFPTNIYDATLAMYEVLKQGGIAPGGFNFDSKVRRASFEVEDLFLAYIAGMDTFAKGLLIAHKLLED
LK3P-081   LNIARINNVLGSIDANQGDLLLGWDTDQFPTNIYDATLAMYEVLKQGGIAPGGFNFDSKVRRASFEVEDLFLAYIAGMDTFAKGLLIAHKLLED
PUC_75     LNIARINNVLGSIDANQGDLLLGWDTDQFPTNIYDATLAMYEVLKQGGIAPGGFNFDSKVRRASFEVEDLFLAYIAGMDTFAKGLLIAHKLLED
S9         LNIARINNVLGSIDANQGDLLLGWDTDQFPTNIYDATLAMYEVLKQGGIAPGGFNFDSKVRRASFEVEDLFLAYIAGMDTFAKGLLIAHKLLED
C103       LNIARINNVLGSIDANQGDLLLGWDTDQFPTNIYDATLAMYEVLKQGGIAPGGFNFDSKVRRASFEVEDLFLAYIAGMDTFAKGLLIAHKLLED
M68        LNIARINNVLGSIDANQGDLLLGWDTDQFPTNIYDATLAMYEVLKQGGIAPGGFNFDSKVRRASFEVEDLFLAYIAGMDTFAKGLLIAHKLLED
PUC_606    LNIARINNVLGSIDANQGDLLLGWDTDQFPTNIYDATLAMYEVLKQGGIAPGGFNFDSKVRRASFEVEDLFLAYIAGMDTFAKGLLIAHKLLED
ICC5       LNIARINNVLGSIDANQGDLLLGWDTDQFPTNIYDATLAMYEVLKQGGIAPGGFNFDSKVRRASFEVEDLFLAYIAGMDTFAKGLLIAHKLLED
M120       LNIARINNVLGSIDANQGDLLLGWDTDQFPTNIYDATLAMYEVLKQGGIAPGGFNFDSKVRRASFEVEDLFLAYIAGMDTFAKGLLIAHKLLED
P8         LNIARINNVLGSIDANQGDLLLGWDTDQFPTNIYDATLAMYEVLKQGGIAPGGFNFDSKVRRASFEVEDLFLAYIAGMDTFAKGLLIAHKLLED
P12        LNIARINNVLGSIDANQGDLLLGWDTDQFPTNIYDATLAMYEVLKQGGIAPGGFNFDSKVRRASFEVEDLFLAYIAGMDTFAKGLLIAHKLLED

             380       390       400       410       420       430       445
R20291     EVFENFTKERYASFSEGIGKDIVEGKVGFKELESYALQMPVIKNKSGRQEMLEAILNRYIYEVDTISNK
PUC_256    EVFENFTKERYASFSEGIGKDIVEGKVGFKELESYALQMPVIKNKSGRQEMLEAILNRYIYEVDTISNK
HC52       EVFENFTKERYASFSEGIGKDIVEGKVGFKELESYALQMPVIKNKSGRQEMLEAILNRYIYEVDTISNK
PUC_90     EVFENFTKERYASFSEGIGKDIVEGKVGFKELESYALQMPVIKNKSGRQEMLEAILNRYIYEVDTISNK
LC5624     EVFENFTKERYASFSEGIGKDIVEGKVGFKELESYALQMPVIKNKSGRQEMLEAILNRYIYEVDTISNK
LK3P-030   EVFENFTKERYASFSEGIGKDIVEGKVGFKELESYALQMPVIKNKSGRQEMLEAILNRYIYEVDTISNK
LK3P-081   EVFENFTKERYASFSEGIGKDIVEGKVGFKELESYALQMPVIKNKSGRQEMLEAILNRYIYEVDTISNK
PUC_75     EVFENFXKERYASFSEGIGKDIVEGKVGFKELESYALQMPVIKNKSGRQEMLEAILNRYIYEVDTISNK
S9         EVFENFTKERYASFSEGIGKDIVEGKVGFKELESYALQMPVIKNKSGRQEMLEAILNRYIYEVDTISNK
C103       EVFENFIKERYASFSEGIGKDIVEGKVGFKELESYALQMPVIKNKSGRQEMLEAILNRYIYEVDTISNK
M68        EVFENFTKERYASFSEGIGKDIVEGKVGFKELESYALQMPVIKNKSGRQEMLEAILNRYIYEVDTISNK
PUC_606    EVFENFTKERYASFSEGIGKDIVEGKVGFKELESYALQMPVIKNKSGRQEMLEAILNRYIYEVDTISNK
ICC5       EVFENFTKERYASFSEGIGKDIVEGKVGFKELESYALQMPVIKNKSGRQEMLEAILNRYIYEVDTISNK
M120       EVFENFTKERYASFSEGIGKDIVEGKVGFKELESYALQMPVIKNKSGRQEMLEAILNRYIYEVDTISNK
P8         EVFENFTKERYASFSEGIGKDIVEGKVGFKELESYALQMPVIKNKSGRQEMLEAILNRYIYEVDTISNK
P12        EVFENFTKERYASFSEGIGKDIVEGKVGFKELESYALQMPVIKNKSGRQEMLEAILNRYIYEVDTISNK
```

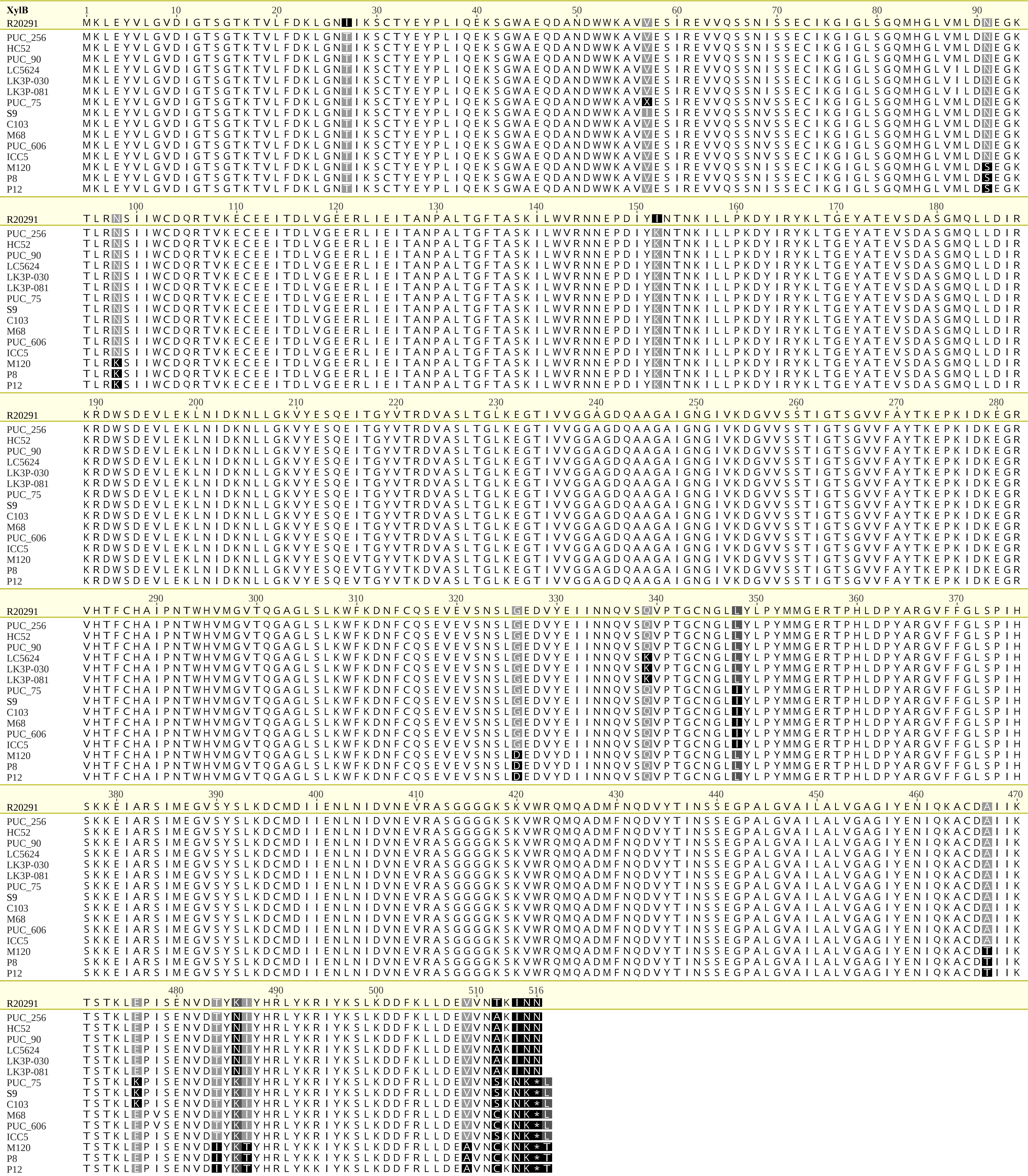
XylB
R20291
PUC_256
HC52
PUC_90
LC5624
LK3P-030
LK3P-081
PUC_75
S9
C103
M68
PUC_606
ICC5
M120
P8
P12

XylR

```
           1                                                                                          90
R20291     MVTDKYTIREMNERLVLEQIIKNGPISRASIASTIGLNKATISAITKKLIDESLVHEIGIGNSTHSGGRKPILLVFNKCAGISLSMDIGYDYIF
PUC_256    MVTDKYTIREMNERLVLEQIIKNGPISRASIASTIGLNKATISAITKKLIDESLVHEIGIGNSTHSGGRKPILLVFNKCAGISLSMDIGYDYIF
HC52       MVTDKYTIREMNERLVLEQIIKNGPISRASIASTIGLNKATISAITKKLIDESLVHEIGIGNSTHSGGRKPILLVFNKCAGISLSMDIGYDYIF
PUC_90     MVTDKYTIREMNERLVLEQIIKNGPISRASIASTIGLNKATISAITKKLIDESLVHEIGIGNSTHSGGRKPILLVFNKCAGISLSMDIGYDYIF
LC5624     MVTDKYTIREMNERLVLEQIIKNGPISRASIASTIGLNKATISAITKKLIDESLVHEIGIGNSTHSGGRKPILLVFNKCAGISLSMDIGYDYIF
LK3P-030   MVTDKYTIREMNERLVLEQIIKNGPISRASIASTIGLNKATISAITKKLIDESLVHEIGIGNSTHSGGRKPILLVFNKCAGISLSMDIGYDYIF
LK3P-081   MVTDKYTIREMNERLVLEQIIKNGPISRASIASTIGLNKATISAITKKLIDESLVHEIGIGNSTHSGGRKPILLVFNKCAGISLSMDIGYDYIF
PUC_75     MVTDKYTIREMNERLVLEQIIKNGPISRASIASTIGLNKATISAITKKLIDESLVHEIGIGNSTHSGGRKPILLVFNKCAGISLSMDIGYDYIF
S9         MVTDKYTIREMNERLVLEQIIKNGPISRASIASTIGLNKATISAITKKLIDESLVHEIGIGNSTHSGGRKPILLVFNKCAGISLSMDIGYDYIF
C103       MVTDKYTIREMNERLVLEQIIKNGPISRASIASTIGLNKATISAITKKLIDESLVHEIGIGNSTHSGGRKPILLVFNKCAGISLSMDIGYDYIF
M68        MVTDKYTIREMNERLVLEQIIKNGPISRASIASTIGLNKATISAITKKLIDESLVHEIGIGNSTHSGGRKPILLVFNKCAGISLSMDIGYDYIF
PUC_606    MVTDKYTIREMNERLVLEQIIKNGPISRASIASTIGLNKATISAITKKLIDESLVHEIGIGNSTHSGGRKPILLVFNKCAGISLSMDIGYDYIF
ICC5       MVTDKYTIREMNERLVLEQIIKNGPISRASIASTIGLNKATISAITKKLIDESLVHEIGIGNSTHSGGRKPILLVFNKCAGISLSMDIGYDYIF
M120       MVTDKYTIREMNERLVLEQIIKNGPISRASIASTIGLNKATISAITKKLIDESLVHEIGIGNSTHSGGRKPILLVFNKCAGISLSMDIGYDYIF
P8         MVTDKYTIREMNERLVLEQIIKNGPISRASIASTIGLNKATISAITKKLIDESLVHEIGIGNSTHSGGRKPILLVFNKCAGISLSMDIGYDYIF
P12        MVTDKYTIREMNERLVLEQIIKNGPISRASIASTIGLNKATISAITKKLIDESLVHEIGIGNSTHSGGRKPILLVFNKCAGISLSMDIGYDYIF

           100                                                                                    180
R20291     SSLSYLDGTIINSKKLTDIQVSKDNVIQLIDEIINSYNISKIDTPYKVIGLTLAIHGITCENKVLFTPYYNLNEIDLYSILSKKYDFPIHIENE
PUC_256    SSLSYLDGTIINSKKLTDIQVSKDNVIQLIDEIINSYNISKIDTPYKVIGLTLAIHGITCENKVLFTPYYNLNEIDLYSILSKKYDFPIHIENE
HC52       SSLSYLDGTIINSKKLTDIQVSKDNVIQLIDEIINSYNISKIDTPYKVIGLTLAIHGITCENKVLFTPYYNLNEIDLYSILSKKYDFPIHIENE
PUC_90     SSLSYLDGTIINSKKLTDIQVSKDNVIQLIDEIINSYNISKIDTPYKVIGLTLAIHGITCENKVLFTPYYNLNEIDLYSILSKKYDFPIHIENE
LC5624     SSLSYLDGTIINSKKLTDIQVSKDNVIQLIDEIINSYNISKIDTPYKVIGLTLAIHGITCENKVLFTPYYNLNEIDLYSILSKKYDFPIHIENE
LK3P-030   SSLSYLDGTIINSKKLTDIQVSKDNVIQLIDEIINSYNISKIDTPYKVIGLTLAIHGITCENKVLFTPYYNLNEIDLYSILSKKYDFPIHIENE
LK3P-081   SSLSYLDGTIINSKKLTDIQVSKDNVIQLIDEIINSYNISKIDTPYKVIGLTLAIHGITCENKVLFTPYYNLNEIDLYSILSKKYDFPIHIENE
PUC_75     SSLSYLDGTIINSKKLTDIQVSKDNVIQLIDEIINSYNISKIDTPYKVIGLTLAIHGITCENKVLFTPYYNLNEIDLYSILSKKYDFPIHIENE
S9         SSLSYLDGTIINSKKLTDIQVSKDNVIQLIDEIINSYNISKIDTPYKVIGLTLAIHGITCENKVLFTPYYNLNEIDLYSILSKKYDFPIHIENE
C103       SSLSYLDGTIINSKKLTDIQVSKDNVIQLIDEIINSYNISKIDTPYKVIGLTLAIHGITCENKVLFTPYYNLNEIDLYSILSKKYDFPIHIENE
M68        SSLSYLDGTIINSKKLTDIQVSKDNVIQLIDEIINSYNISKIDTPYKVIGLTLAIHGITCENKVLFTPYYNLNEIDLYSILSKKYDFPIHIENE
PUC_606    SSLSYLDGTIINSKKLTDIQVSKDNVIQLIDEIINSYNISKIDTPYKVIGLTLAIHGITCENKVLFTPYYNLNEIDLYSILSKKYDFPIHIENE
ICC5       SSLSYLDGTIINSKKLTDIQVSKDNVIQLIDEIINSYNISKIDTPYKVIGLTLAIHGITCENKVLFTPYYNLNEIDLYSILSKKYDFPIHIENE
M120       SSMSYLDGTIINSKKLTDIQVSKDNVIQLIDEIINSYNISKIDTPYKVIGLTLAIHGITCENKVLFTPYYNLNEIDLYSILSKKYDFPIHIENE
P8         SSMSYLDGTIINSKKLTDIQVSKDNVIQLIDEIINSYNISKIDTPYKVIGLTLAIHGITCENKVLFTPYYNLNEIDLYSILSKKYDFPIHIENE
P12        SSMSYLDGTIINSKKLTDIQVSKDNVIQLIDEIINSYNISKIDTPYKVIGLTLAIHGITCENKVLFTPYYNLNEIDLYSILSKKYDFPIHIENE

           190                                                                                    280
R20291     ANLTALAENTFSTVHNSLLSLSIHSGFGSGIIINNKLYSGRNGMSGEIGHTIIMPNGKLCPCGNRGCLEQYCSEKKVFEQLSSLENIPKIDSDI
PUC_256    ANLTALAENTFSTVHNSLLSLSIHSGFGSGIIINNKLYSGRNGMSGEIGHTIIMPNGKLCPCGNRGCLEQYCSEKKVFEQLSSLENIPKIDSDI
HC52       ANLTALAENTFSTVHNSLLSLSIHSGFGSGIIINNKLYSGRNGMSGEIGHTIIMPNGKLCPCGNRGCLEQYCSEKKVFEQLSSLENIPKIDSDI
PUC_90     ANLTALAENTFSTVHNSLLSLSIHSGFGSGIIINNKLYSGRNGMSGEIGHTIIMPNGKLCPCGNRGCLEQYCSEKKVFEQLSSLENIPKIDSDI
LC5624     ANLTALAENTFSTVHNSLLSLSIHSGFGSGIIINNKLYSGRNGMSGEIGHTIIMPNGKLCPCGNRGCLEQYCSEKKVFEQLSSLENIPKIDSDI
LK3P-030   ANLTALAENTFSTVHNSLLSLSIHSGFGSGIIINNKLYSGRNGMSGEIGHTIIMPNGKLCPCGNRGCLEQYCSEKKVFEQLSSLENIPKIDSDI
LK3P-081   ANLTALAENTFSTVHNSLLSLSIHSGFGSGIIINNKLYSGRNGMSGEIGHTIIMPNGKLCPCGNRGCLEQYCSEKKVFEQLSSLENIPKIDSDI
PUC_75     ANLTALAEXTFSTVHNSLLSLSIHSGFGSGIIINNKLYSGRNGMSGEIGHTIIMPNGKLCPCGNRGCLEQYCSEKKVFEQLSSLENIPKIDSDI
S9         ANLTALAENTFSTVHNSLLSLSIHSGFGSGIIINNKLYSGRNGMSGEIGHTIIMPNGKLCPCGNRGCLEQYCSEKKVFEQLSSLENIPKIDSDI
C103       ANLTALAENTFSTVHNSLLSLSIHSGFGSGIIINNKLYSGRNGMSGEIGHTIIMPNGKLCPCGNRGCLEQYCSEKKVFEQLSSLENIPKIDSDI
M68        ANLTALAENTFSTVHNSLLSLSIHSGFGSGIIINNKLYSGRNGMSGEIGHTIIMPNGKLCPCGNRGCLEQYCSEKKVFEQLSSLENIPKIDSDI
PUC_606    ANLTALAENTFSTVHNSLLSLSIHSGFGSGIIINNKLYSGRNGMSGEIGHTIIMPNGKLCPCGNRGCLEQYCSEKKVFEQLSSLENIPKIDSDI
ICC5       ANLTALAENTFSTVHNSLLSLSIHSGFGSGIIINNKLHSGRNGMSGEIGHTIIMPNGKLCPCGNRGCLEQYCSEKKVFEQLSSLENIPKIDSDI
M120       ANLTALAENTFSTVHDSLLSLSIHSGFGSGIIINNKLYSGRNGMSGEIGHTIIMPNGKLCPCGNRGCLEQYCSEKKVFEQLSSLENIPKIDSDI
P8         ANLTALAENTFSTVHDSLLSLSIHSGFGSGIIINNKLYSGRNGMSGEIGHTIIMPNGKLCPCGNRGCLEQYCSEKKVFEQLSSLENIPKIDSDI
P12        ANLTALAENTFSTVHDSLLSLSIHSGFGSGIIINNKLYSGRNGMSGEIGHTIIMPNGKLCPCGNRGCLEQYCSEKKVFEQLSSLENIPKIDSDI

           290                                                                                370
R20291     VKQLYYEDNQNAKKVIHEFCSYLTIAINNAITTYAPEIIYLNSQIISDIPEILQITKDMLVSSFNKGINIEISSLGSEASLYGGSAVNIKSFLN
PUC_256    VKQLYYEDNHNAKKVIHEFCSYLTIAINNAITTYAPEIIYLNSQIISDIPEILQITKDMLVSSFNKGINIEISSLGSEASLYGGSAVNIKSFLN
HC52       VKQLYYEDNHNAKKVIHEFCSYLTIAINNAITTYAPEIIYLNSQIISDIPEILQITKDMLVSSFNKGINIEISSLGSEASLYGGSAVNIKSFLN
PUC_90     VKQLYYEDNHNAKKVIHEFCSYLTIAINNAITTYAPEIIYLNSQIISDIPEILQITKDMLVSSFNKGINIEISSLGSEASLYGGSAVNIKSFLN
LC5624     VKQLYYEDNQNAKKVIHEFCSYLTIAINNAITTYAPEIIYLNSQIISDIPEILQITKDMLVSSFNKGINIEISSLGSEASLYGGSAVNIKSFLN
LK3P-030   VKQLYYEDNQNAKKVIHEFCSYLTIAINNAITTYAPEIIYLNSQIISDIPEILQITKDMLVSSFNKGINIEISSLGSEASLYGGSAVNIKSFLN
LK3P-081   VKQLYYEDNQNAKKVIHEFCSYLTIAINNAITTYAPEIIYLNSQIISDIPEILQITKDMLVSSFNKGINIEISSLGSEASLYGGSAVNIKSFLN
PUC_75     VKQLYYEDNQNTKKVIHEFCSYLTIAINNAITTYAPEIIYLNSQIISDIPEILQITKDMLVSSFNKGINIEISSLGSEASLYGGSAVNIKSFLN
S9         VKQLYYEDNQNTKKVIHEFCSYLTIAINNAITTYAPEIIYLNSQIISDIPEILQITKDMLVSSFNKGINIEISSLGSEASLYGGSAVNIKSFLN
C103       VKQLYYEDNQNTKKVIHEFCSYLTIAINNAITTYAPEIIYLNSQIISDIPEILQITKDMLVSSFNKGINIEISSLGSEASLYGGSAVNIKSFLN
M68        VKQLYYEDNQNAKKVIHEFCSYLTIAINNAITTYAPEIIYLNSQIISDIPEILQITKDMLVSSFNKGINIEISSLGSEASLYGGSAVNIKSFLN
PUC_606    VKQLYYEDNQNAKKVIHEFCSYLTIAINNAITTYAPEIIYLNSQIISDIPEILQITKDMLVSSFNKGINIEISSLGSEASLYGGSAVNIKSFLN
ICC5       VKQLYYEDNQNAKKVIHEFCSYLTIAINNAITTYAPEIIYLNSQIISDIPEILQITKDMLVSSFNKGINIEISSLGSEASLYGGSAVNIKSFLN
M120       VKKLYYEDNQNTKKVIHEFCSYLTIAINNAITTYAPEIIYLNSQIISDIPEILQITKDMLVSSFNKGINIEISSLGSEASLYGGSAVNIKSFLN
P8         VKKLYYEDNQNTKKVIHEFCSYLTIAINNAITTYAPEIIYLNSQIISDIPEILQITKDMLVSSFNKGINIEISSLGSEASLYGGSAVNIKSFLN
P12        VKKLYYEDNQNTKKVIHEFCSYLTIAINNAITTYAPEIIYLNSQIISDIPEILQITKDMLVSSFNKGINIEISSLGSEASLYGGSAVNIKSFLN

                    386
R20291     IQNLTLINEI
PUC_256    IQNLTLINEI
HC52       IQNLTLINEI
PUC_90     IQNLTLINEI
LC5624     IQNLTLINEI
LK3P-030   IQNLTLINEI
LK3P-081   IQNLTLINEI
PUC_75     IQNLTLINEI
S9         IQNLTLINEI
C103       IQNLTLINEI
M68        IQNLTLINEI
PUC_606    IQNLTLINEI
ICC5       IQNLTLINEI
M120       IQNLTLINEI
P8         IQNLTLINEI
P12        IQNLTLINEI
```
