# Supplementary material for: Phenotypic analysis of various Clostridioides difficile ribotypes reveals consistency among core processes
Source: Appl Environ Microbiol. 2025 Jun 24;91(7):e00964-25. doi: 10.1128/aem.00964-25 (PMC12285255; doi:10.1128/aem.00964-25)
Supplement: Supplemental figures, part II — Figures S9 to S15. [file aem.00964-25-s0002.pdf]

TreA

```
          1      8         18        28        38        48        58        68        78        88
R20291    MKNWWKKATVYQIYPKSFKDSNNDGIGDINGIIEKLDYLYSLGVDLLWLTPMYVSPQRDNGYDIEDYYNIDPKYGTMSDFEKLLKEAHKRD
PUC_256   MKNWWKKATVYQIYPKSFKDSNNDGIGDINGIIEKLDYLYYLGVDLLWLTPMYVSPQRDNGYDIEDYYNIDPKYGTMSDFEKLLKEAHKRD
HC52      MKNWWKKATVYQIYPKSFKDSNNDGIGDINGIIEKLDYLYYLGVDLLWLTPMYVSPQRDNGYDIEDYYNIDPKYGTMSDFEKLLKEAHKRD
PUC_90    MKNWWKKATVYQIYPKSFKDSNNDGIGDINGIIEKLDYLYYLGVDLLWLTPMYVSPQRDNGYDIEDYYNIDPKYGTMSDFEKLLKEAHKRD
LC5624    MKNWWKKATVYQIYPKSFKDSNNDGIGDINGIIEKLDYLYSLGVDLLWLTPMYVSPQRDNGYDIEDYYNIDPKYGTMSDFEKLLKEAHKRD
LK3P-030  MKNWWKKATVYQIYPKSFKDSNNDGIGDINGIIEKLDYLYSLGVDLLWLTPMYVSPQRDNGYDIEDYYNIDPKYGTMSDFEKLLKEAHKRD
LK3P-081  MKNWWKKATVYQIYPKSFKDSNNDGIGDINGIIEKLDYLYYLGVDLLWLTPMYVSPQRDNGYDIEDYYNIDPKYGTMSDFEKLLKEAHKRD
M68       MKNWWKKATVYQIYPKSFKDSNNDGIGDINGIIEKLDYLSSLGVDLLWLTPMYVSPQRDNGYDIEDYYNIDPKYGTMSDFEKLLKEAHKRD
ICC5      MKNWWKKATVYQIYPKSFKDSNNDGIGDINGIIEKLDYLSSLGVDLLWLTPMYVSPQRDNGYDIEDYYNIDPKYGTMSDFEKLLKEAHKRD
PUC_606   MKNWWKKATVYQIYPKSFKDSNNDGIGDINGIIEKLDYLSSLGVDLLWLTPMYVSPQRDNGYDIEDYYNIDPKYGTMSDFEKLLKEAHKRD

                98        108       118       128       138       148       158       168       178
R20291    IKIMMDMVLNHTSTEHKWFKESKKSKDNPYRDYYFWKDAKPDGSVPNNWISRFSGTAWKYDETTNQYYLHLFEETQADLNLENEKVREECYKV
PUC_256   IKIMMDMVLNHTSTEHKWFKESKKSKDNPYRDYYFWKDAKPDGSVPNNWISRFSGTAWKYDETTNQYYLHLFEETQADLNWENEKVREECYKI
HC52      IKIMMDMVLNHTSTEHKWFKESKKSKDNPYRDYYFWKDAKPDGSVPNNWISRFSGTAWKYDETTNQYYLHLFEETQADLNWENEKVREECYKI
PUC_90    IKIMMDMVLNHTSTEHKWFKESKKSKDNPYRDYYFWKDAKPDGSVPNNWISRFSGTAWKYDETTNQYYLHLFEETQADLNWENEKVREECYKI
LC5624    IKIMMDMVLNHTSTEHKWFKESKKSKDNPYRDYYFWKDAKPDGSVPNNWISRFSGTAWKYDETTNQYYLHLFEETQADLNWENEKVREECYKI
LK3P-030  IKIMMDMVLNHTSTEHKWFKESKKSKDNPYRDYYFWKDAKPDGSVPNNWISRFSGTAWKYDETTNQYYLHLFEETQADLNWENEKVREECYKI
LK3P-081  IKIMMDMVLNHTSTEHKWFKESKKSKDNPYRDYYFWKDAKPDGSVPNNWISRFSGTAWKYDETTNQYYLHLFEETQADLNWENEKVREECYKI
M68       IKIMMDMVLNHTSTEHKWFKESKKSKDNPYRDYYFWKDAKPDGSVPNNWISRFSGTAWKYDETTNQYYLHLFEETQADLNWENEKVREECYKI
ICC5      IKIMMDMVLNHTSTEHKWFKESKKSKDNPYRDYYFWKDAKPDGSVPNNWISRFSGTAWKYDETTNQYYLHLFEETQADLNWENEKVREECYKI
PUC_606   IKIMMDMVLNHTSTEHKWFKESKKSKDNPYRDYYFWKDAKPDGSVPNNWISRFSGTAWKYDETTNQYYLHLFEETQADLNWENEKVREECYKI

             188       198       208       218       228       238       248       258       268
R20291    LEFWADKGIDGFRLDVVNLLSKTPGLPDDPITGPKGDGRTHYADGPRIHEYLHNMNQKVFKPKNIVTVGEMSSTTPEECINYTRENREELSMV
PUC_256   LEFWADKGIDGFRLDVVNLLSKTPGLPDDPITGPKGDGRTHYADGPRIHEYLHNMNQKVFKPKNIVTVGEMSSTTPEECINYTRENREELSMV
HC52      LEFWADKGIDGFRLDVVNLLSKTPGLPDDPITGPKGDGRTHYADGPRIHEYLHNMNQKVFKPKNIVTVGEMSSTTPEECINYTRENREELSMV
PUC_90    LEFWADKGIDGFRLDVVNLLSKTPGLPDDPITGPKGDGRTHYADGPRIHEYLHNMNQKVFKPKNIVTVGEMSSTTPEECINYTRENREELSMV
LC5624    LEFWADKGIDGFRLDVVNLLSKTPGLPDDPITGPKGDGRTHYADGPRIHEYLHNMNQKVFKPKNIVTVGEMSSTTPEECINYTRENREELSMV
LK3P-030  LEFWADKGIDGFRLDVVNLLSKTPGLPDDPITGPKGDGRTHYADGPRIHEYLHNMNQKVFKPKNIVTVGEMSSTTPEECINYTRENREELSMV
LK3P-081  LEFWADKGIDGFRLDVVNLLSKTPGLPDDPITGPKGDGRTHYADGPRIHEYLHNMNQKVFKPKNIVTVGEMSSTTPEECINYTRENREELSMV
M68       LEFWADKGIDGFRLDVVNLLSKTPGLPDDPITGPKGDGRTHYADGPRIHEYLHNMNQKVFKPKNIVTVGEMSSTTPEECINYTRENREELSMV
ICC5      LEFWADKGIDGFRLDVVNLLSKTPGLPDDPITGPKGDGRTHYADGPRIHEYLHNMNQKVFKPKNIVTVGEMSSTTPEECINYTRENREELSMV
PUC_606   LEFWADKGIDGFRLDVVNLLSKTPGLPDDPITGPKGDGRTHYADGPRIHEYLHNMNQKVFKPKNIVTVGEMSSTTPEECINYTRENREELSMV

          278       288       298       308       318       328       338       348       358       368
R20291    FSFHHMKTDYKGGAKWTNEMFSLDKLKKAQSDFQYKMYEGKGWNALFYSNHDQPRALSRFGDDRFFRQESAKMLAITLFGLQGTTYIYQGEEI
PUC_256   FSFHHMKTDYKGGAKWTNEMFSLDKLKKAQSDFQYKMYEGKGWNALFYSNHDQPRALSRFGDDRFFRQESAKMLAITLFGLQGTTYIYQGEEI
HC52      FSFHHMKTDYKGGAKWTNEMFSLDKLKKAQSDFQYKMYEGKGWNALFYSNHDQPRALSRFGDDRFFRQESAKMLAITLFGLQGTTYIYQGEEI
PUC_90    FSFHHMKTDYKGGAKWTNEMFSLDKLKKAQSDFQYKMYEGKGWNALFYSNHDQPRALSRFGDDRFFRQESAKMLAITLFGLQGTTYIYQGEEI
LC5624    FSFHHMKTDYKGGAKWTNEMFSLDKLKKAQSDFQYKMYEGKGWNALFYSNHDQPRALSRFGDDRFFRQESAKMLAITLFGLQGTTYIYQGEEI
LK3P-030  FSFHHMKTDYKGGAKWTNEMFSLDKLKKAQSDFQYKMYEGKGWNALFYSNHDQPRALSRFGDDRFFRQESAKMLAITLFGLQGTTYIYQGEEI
LK3P-081  FSFHHMKTDYKGGAKWTNEMFSLDKLKKAQSDFQYKMYEGKGWNALFYSNHDQPRALSRFGDDRFFRQESAKMLAITLFGLQGTTYIYQGEEI
M68       FSFHHMKTDYKGGAKWTNEMFSLDKLKKAQSDFQYKMYEGKGWNALFYSNHDQPRALSRFGDDRFFRQESAKMLAITLFGLQGTTYIYQGEEI
ICC5      FSFHHMKTDYKGGAKWTNEMFSLDKLKKAQSDFQYKMYEGKGWNALFYSNHDQPRALSRFGDDRFFRQESAKMLAITLFGLQGTTYIYQGEEI
PUC_606   FSFHHMKTDYKGGAKWTNEMFSLDKLKKAQSDFQYKMYEGKGWNALFYSNHDQPRALSRFGDDRFFRQESAKMLAITLFGLQGTTYIYQGEEI

                  378       388       398       408       418       428       438       448       458
R20291    GMTNAYFTKIDEYDDAESKNAYYHMIKSGIDEKEALLILQQKSRDNARTPMQWDNTIYKGFSNHKPWLKVNNDNISVENELNDSRSVFYTYQR
PUC_256   GMTNAYFTKIDEYDDAESKNAYYHMIKSGIDEKEALLILQQKSRDNARTPMQWDNTIYKGFSNHKPWLKVNNDNISVENELNDSRSVFYTYQR
HC52      GMTNAYFTKIDEYDDAESKNAYYHMIKSGIDEKEALLILQQKSRDNARTPMQWDNTIYKGFSNHKPWLKVNNDNISVENELNDSRSVFYTYQR
PUC_90    GMTNAYFTKIDEYDDAESKNAYYHMIKSGIDEKEALLILQQKSRDNARTPMQWDNTIYKGFSNHKPWLKVNNDNISVENELNDSRSVFYTYQR
LC5624    GMTNAYFTKIDEYDDAESKNAYYHMIKSGIDEKEALLILQQKSRDNARTPMQWDNTIYKGFSNHKPWLKVNNDNISVENELNDSRSVFYTYQR
LK3P-030  GMTNAYFTKIDEYDDAESKNAYYHMIKSGIDEKEALLILQQKSRDNARTPMQWDNTIYKGFSNHKPWLKVNNDNISVENELNDSRSVFYTYQR
LK3P-081  GMTNAYFTKIDEYDDAESKNAYYHMIKSGIDEKEALLILQQKSRDNARTPMQWDNTIYKGFSNHKPWLKVNNDNISVENELNDSRSVFYTYQR
M68       GMTNAYFTKIDEYDDAESKNAYYHMIKSGIDEKEALLILQQKSRDNARTPMQWDNTIYKGFSNHKPWLKVNNDNISVENELNDSRSVFYTYQR
ICC5      GMTNAYFTKIDEYDDAESKNAYYHMIKSGIDEKEALLILQQKSRDNARTPMQWDNTIYKGFSNHKPWLKVNNDNISVENELNDSRSVFYTYQR
PUC_606   GMTNAYFTKIDEYDDAESKNAYYHMIKSGIDEKEALLILQQKSRDNARTPMQWDNTIYKGFSNHKPWLKVNNDNISVENELNDSRSVFYTYQR

              468       478       488       498       508       518       528       538             555
R20291    LIKYRKQYDIFTEGTYRLLDENHKNLFVYEREYKNQNLLVISNFTHDNVVYKLPETYLNKLNYTILETNYTRDIIENKMEIKPYESIMIYYK
PUC_256   LIKYRKQYDIFTEGTYRLLDENHKNLFVYEREYKNQNLLVISNFTHDNVVYKLPETYLNKLNYTILETNYTRDIIENKMEIKPYESIMIYYK
HC52      LIKYRKQYDIFTEGTYRLLDENHKNLFVYEREYKNQNLLVISNFTHDNVVYKLPETYLNKLNYTILETNYTRDIIENKMEIKPYESIMIYYK
PUC_90    LIKYRKQYDIFTEGTYRLLDENHKNLFVYEREYKNQNLLVISNFTHDNVVYKLPETYLNKLNYTILETNYTRDIIENKMEIKPYESIMIYYK
LC5624    LIKYRKQYDIFTEGTYRLLDENHKNLFVYEREYKNQNLLVISNFTHDNVVYKLPETYLNKLNYTILETNYTRDIIENKMEIKPYESIMIYYK
LK3P-030  LIKYRKQYDIFTEGTYRLLDENHKNLFVYEREYKNQNLLVISNFTHDNVVYKLPETYLNKLNYTILETNYTRDIIENKMEIKPYESIMIYYK
LK3P-081  LIKYRKQYDIFTEGTYRLLDENHKNLFVYEREYKNQNLLVISNFTHDNVVYKLPETYLNKLNYTILETNYTRDIIENKMEIKPYESIMIYYK
M68       LIKYRKQYDIFTEGTYRLLDENHKNLFVYEREYKKQNLLVISNFTHDNVVYKLPETYLNKLNYTILETNYTRDIIENEMEIKPYESIMIYYK
ICC5      LIKYRKQYDIFTEGTYRLLDENHKNLFVYEREYKKQNLLVISNFTHDNVVYKLPETYLNKLNYTILETNYTRDIIENEMEIKPYESIMIYYK
PUC_606   LIKYRKQYDIFTEGTYRLLDENHKNLFVYEREYKKQNLLVISNFTHDNVVYKLPETYLNKLNYTILETNYTRDIIENEMEIKPYESIMIYYK
```

TreA

```
      1        10        20        30        40        50        60        70        80        90        100       110
M120  MKNWWKKATVYQIYPKSFKDSNNDGIGDINGIIEKLDYLSSLGVDLLWLTPMYVSPQRDNGYDIEDYYNIDPKYGTMSDFEELLKEAHKRDLKIMMDMVLNHTSTEHKWFK
P8    MKNWWKKATVYQIYPKSFKDSNNDGIGDINGIIEKLDYLSSLGVDLLWLTPMYVSPQRDNGYDIEDYYNIDPKYGTMSDFEELLKEAHKRDLKIMMDMVLNHTSTEHKWFK
P12   MKNWWKKATVYQIYPKSFKDSNNDGIGDINGIIEKLDYLSSLGVDLLWLTPMYVSPQRDNGYDIEDYYNIDPKYGTMSDFEELLKEAHKRDLKIMMDMVLNHTSTEHKWFK

      120       130       140       150       160       170       180       190       200       210       220
M120  ESKKSKDNPYRDYYFWKDAKPDGSVPNNWISRFSGTAWKYDKTTNQYYLHLFEETQADLNWENEKVREECYKILEFWADKGIDGFRLDVVNLLSKTPGLPDDPITGPKGDG
P8    ESKKSKDNPYRDYYFWKDAKPDGSVPNNWISRFSGTAWKYDKTTNQYYLHLFEETQADLNWENEKVREECYKILEFWADKGIDGFRLDVVNLLSKTPGLPDDPITGPKGDG
P12   ESKKSKDNPYRDYYFWKDAKPDGSVPNNWISRFSGTAWKYDKTTNQYYLHLFEETQADLNWENEKVREECYKILEFWADKGIDGFRLDVVNLLSKTPGLPDDPITGPKGDG

      230       240       250       260       270       280       290       300       310       320       330
M120  RTHYADGPRIHEYLHNMNQKVFKPKNIVTVGEMSSTTPEECINYTRENREELSMVFSFHHMKTDYKGGVKWTNEMFSLDKLKKAQSDFQYKMYEGKGWNALFYSNHDQPRA
P8    RTHYADGPRIHEYLHNMNQKVFKPKNIVTVGEMSSTTPEECINYTRENREELSMVFSFHHMKTDYKGGVKWTNEMFSLDKLKKAQSDFQYKMYEGKGWNALFYSNHDQPRA
P12   RTHYADGPRIHEYLHNMNQKVFKPKNIVTVGEMSSTTPEECINYTRENREELSMVFSFHHMKTDYKGGVKWTNEMFSLDKLKKAQSDFQYKMYEGKGWNALFYSNHDQPRA

      340       350       360       370       380       390       400       410       420       430       440
M120  LSRFGDDRFFRQESAKMLAITLFGLQGTTYIYQGEEIGMTNAYFTKIDEYDDAESKNAYYHMIKSGIDEKEALIILQQKSRDNARTPMQWDNTIYKGFSNHKPWLKVNNDN
P8    LSRFGDDRFFRQESAKMLAITLFGLQGTTYIYQGEEIGMTNAYFTKIDEYDDAESKNAYYHMIKSGIDEKEALIILQQKSRDNARTPMQWDNTIYKGFSNHKPWLKVNNDN
P12   LSRFGDDRFFRQESAKMLAITLFGLQGTTYIYQGEEIGMTNAYFTKIDEYDDAESKNAYYHMIKSGIDEKEALIILQQKSRDNARTPMQWDNTIYKGFSNHKPWLKVNNDN

      450       460       470       480       490       500       510       520       530       540       555
M120  ISVENELNDSHSVFYTYQRLIKYRKQYDIFTEGTYRLLDENHKNLFVYEREYKNQNILVISNFTHDNVVYKLPETYLNKLNYTILETNYTRDTIENEMEIKPYESIMIYCK
P8    ISVENELNDSHSVFYTYQRLIKYRKQYDIFTEGTYRLLDENHKNLFVYEREYKNQNILVISNFTHDNVVYKLPETYLNKLNYTILETNYTRDTIENEMEIKPYESIMIYCK
P12   ISVENELNDSHSVFYTYQRLIKYRKQYDIFTEGTYRLLDENHKNLFVYEREYKNQNILVISNFTHDNVVYKLPETYLNKLNYTILETNYTRDTIENEMEIKPYESIMIYCK
```

TreR

Strain	1–93
R20291	MAKNKFSEIYEVLKEEILDGKYTSNMMLPTELQLIERFSCSRNTVRRAISQLNTEGYVQSIKGKGVVVLENSCSNDFFLNMHNFKGVESIVED
PUC_256	MAKNKFSEIYEVLKEEILDGKYTSNMMLPTELQLIERFSCSRNTVRRAISQLNTEGYVQSIKGKGVVVLENSCSNDFFLNMHNFKGVESIVED
HC52	MAKNKFSEIYEVLKEEILDGKYTSNMMLPTELQLIERFSCSRNTVRRAISQLNTEGYVQSIKGKGVVVLENSCSNDFFLNMHNFKGVESIVED
PUC_90	MAKNKFSEIYEVLKEEILDGKYTSNMMLPTELQLIERFSCSRNTVRRAISQLNTEGYVQSIKGKGVVVLENSCSNDFFLNMHNFKGVESIVED
LC5624	MAKNKFSEIYEVLKEEILDGKYTSNMMLPTELQLIERFSCSRNTVRRAISQLNTEGYVQSIKGKGVVVLENSCSNDFFLNMHNFKGVESIVED
LK3P-030	MAKNKFSEIYEVLKEEILDGKYTSNMMLPTELQLIERFSCSRNTVRRAISQLNTEGYVQSIKGKGVVVLENSCSNDFFLNMHNFKGVESIVED
LK3P-081	MAKNKFSEIYEVLKEEILDGKYTSNMMLPTELQLIERFSCSRNTVRRAISQLNTEGYVQSIKGKGVVVLENSCSNDFFLNMHNFKGVESIVED
M68	MAKNKFSEIYEVLKEEILDGKYTSNMMLPTELQLIERFSCSRNTVRRAISQLNAEGYVQSIKGKGVVVLENSCSNDFFLNMHNFKGVESIVED
PUC_606	MAKNKFSEIYEVLKEEILDGKYTSNMMLPTELQLIERFSCSRNTVRRAISQLNAEGYVQSIKGKGVVVLENSCSNDFFLNMHNFKGVESIVED
ICC5	MAKNKFSEIYEVLKEEILDGKYTSNMMLPTELQLIERFSCSRNTVRRAISQLNAEGYVQSIKGKGVVVLENSCSNDFFLNMHNFKGVESIVED

Strain	94–186
R20291	KKVNTATSVLHFSKILIDNKLSKKTGFKVGSEVYYLHRLRYIDNIPKILDINYFLCSIVKDLDVSIAQGSIYKYIEECIGTKIVSSRKIFKIE
PUC_256	KKVNTATSVLHFSKILIDNKLSKKTGFKVGSEVYYLHRLRYIDNIPKILDINYFLCSIVKDLDVSIAQGSIYKYIEECLGTKIVSSRKIFKIE
HC52	KKVNTATSVLHFSKILIDNKLSKKTGFKVGSEVYYLHRLRYIDNIPKILDINYFLCSIVKDLDVSIAQGSIYKYIEECLGTKIVSSRKIFKIE
PUC_90	KKVNTATSVLHFSKILIDNKLSKKTGFKVGSEVYYLHRLRYIDNIPKILDINYFLCSIVKDLDVSIAQGSIYKYIEECLGTKIVSSRKIFKIE
LC5624	KKVNTATSVLHFSKILIDNKLSKKTGFKVGSEVYYLHRLRYIDNIPKILDINYFLCSIVKDLDVSIAQGSIYKYIEECLGTKIVSSRKIFKIE
LK3P-030	KKVNTATSVLHFSKILIDNKLSKKTGFKVGSEVYYLHRLRYIDNIPKILDINYFLCSIVKDLDVSIAQGSIYKYIEECLGTKIVSSRKIFKIE
LK3P-081	KKVNTATSVLHFSKILIDNKLSKKTGFKVGSEVYYLHRLRYIDNIPKILDINYFLCSIVKDLDVSIAQGSIYKYIEECLGTKIVSSRKIFKIE
M68	KKVNTATSVLHFSKILIDNKLSKKTGFKVGSEVYYLHRLRYIDNIPKILDINYFLCSIVKNLDVSIAQGSIYKYIEESLGTKIVSSRKIFKIE
PUC_606	KKVNTATSVLHFSKILIDNKLSKKTGFKVGSEVYYLHRLRYIDNIPKILDINYFLCSIVKNLDVSIAQGSIYKYIEESLGTKIVSSRKIFKIE
ICC5	KKVNTATSVLHFSKILIDNKLSKKTGFKVGSEVYYLHRLRYIDNIPKILDINYFLCSIVKDLDVSIAQGSIYKYIEESLGTKIVSSRKIFKIE

Strain	187–240
R20291	KATELELKTLPLNDYNCVGVIKNSVYTDDGKLFEYTESKHTPETFVFMDVTQRY
PUC_256	KATELELKTLPLNDYNCVGVIKNSVYTDDGKLFEYTESKHTPETFVFMDVTQRY
HC52	KATELELKTLPLNDYNCVGVIKNSVYTDDGKLFEYTESKHTPETFVFMDVTQRY
PUC_90	KATELELKTLPLNDYNCVGVIKNSVYTDDGKLFEYTESKHTPETFVFMDVTQRY
LC5624	KATELELKTLPLNDYNCVGVIKNSVYTDDGKLFEYTESKHTPETFVFMDVTQRY
LK3P-030	KATELELKTLPLNDYNCVGVIKNSVYTDDGKLFEYTESKHTPETFVFMDVTQRY
LK3P-081	KATELELKTLPLNDYNCVGVIKNSVYTDDGKLFEYTESKHTPETFVFMDVTQRY
M68	KATELELKTLPLNDYNCVGVIKNSVYTDDGKLFEYTESKHTPETFVFMDVTQRY
PUC_606	KATELELKTLPLNDYNCVGVIKNSVYTDDGKLFEYTESKHTPETFVFMDVTQRY
ICC5	KATELELKTLPLNDYNCVGVIKNSVYTDDGKLFEYTESKHTPETFVFMDVTQRY

TreR

```
        1        10        20        30        40        50        60        70        80        90        100       110       120
M120    MAKNKFSEIYEVLKEEILDGKYTSNMMLPTELQLIERFSCSRNTVRRAISQLNAEGYVQSIKGKGVVVLENSCSNDFFLNMHNFKGVESIVEDKKVNTATSVLHFSKILIDNKLSKKTGF
P8      MAKNKFSEIYEVLKEEILDGKYTSNMMLPTELQLIERFSCSRNTVRRAISQLNAEGYVQSIKGKGVVVLENSCSNDFFLNMHNFKGVESIVEDKKVNTATSVLHFSKILIDNKLSKKTGF
P12     MAKNKFSEIYEVLKEEILDGKYTSNMMLPTELQLIERFSCSRNTVRRAISQLNAEGYVQSIKGKGVVVLENSCSNDFFLNMHNFKGVESIVEDKKVNTATSVLHFSKILIDNKLSKKTGF

                 130       140       150       160       170       180       190       200       210       220       230       240
M120    KIGSEVYYLHRLRYIDNVPKILDINYFLCSIVKDLDVSIAQGSIYKYIEECLGTKIVSSRKIFKIEKATELELKTLPLNDYNCVGVIKNSVYTDDGKLFEYTESKHTPETFVFMDVTQRY
P8      KIGSEVYYLHRLRYIDNVPKILDINYFLCSIVKDLDVSIAQGSIYKYIEECLGTKIVSSRKIFKIEKATELELKTLPLNDYNCVGVIKNSVYTDDGKLFEYTESKHTPETFVFMDVTQRY
P12     KIGSEVYYLHRLRYIDNVPKILDINYFLCSIVKDLDVSIAQGSIYKYIEECLGTKIVSSRKIFKIEKATELELKTLPLNDYNCVGVIKNSVYTDDGKLFEYTESKHTPETFVFMDVTQRY
```

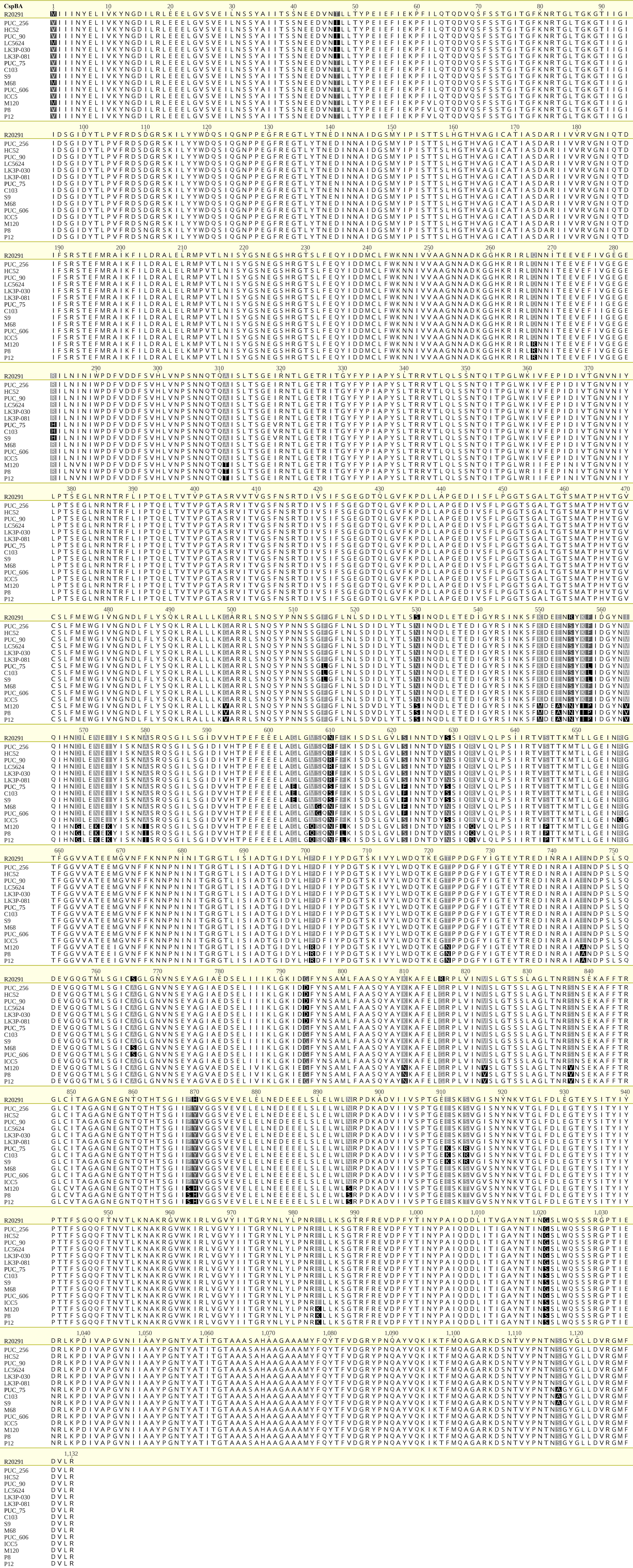
CspBA
R20291
PUC_256
HC52
PUC_90
LC5624
LK3P-030
LK3P-081
PUC_75
C103
S9
M68
PUC_606
ICC5
M120
P8
P12

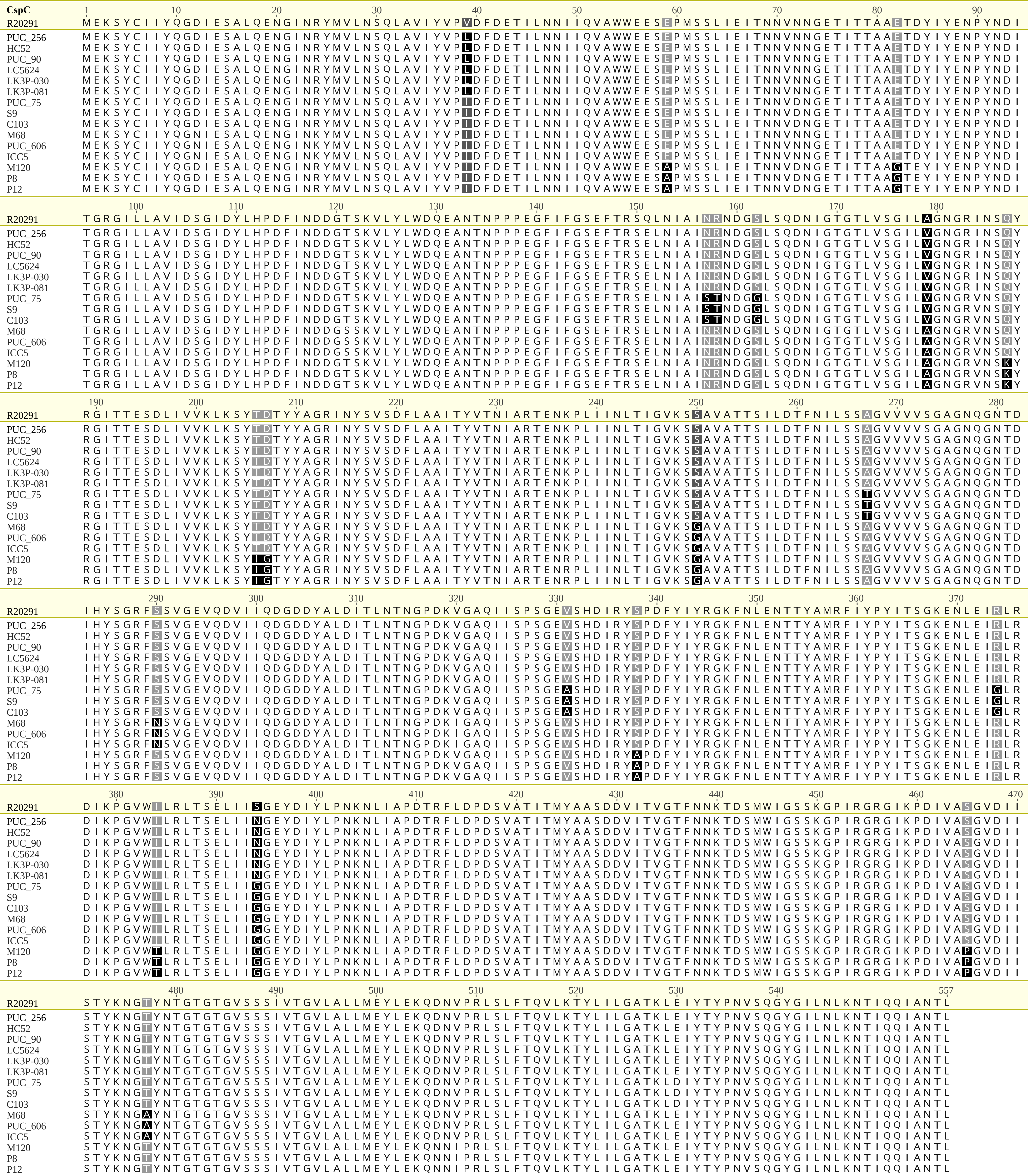
CspC
R20291
PUC_256
HC52
PUC_90
LC5624
LK3P-030
LK3P-081
PUC_75
S9
C103
M68
PUC_606
ICC5
M120
P8
P12

SleC

```
           1        10        20        30        40        50        60        70        80        90
R20291     MQDGFLTVSIIDATNNRPIQNAVVNIYSMSNGSQSSSTLYQNLRSNESGQVTGLVLPAPDVDYSLQPSDVRPYSQYIVEAIADGYETVVIEGTQ

PUC_256    MQDGFLTVSIIDATNNRPIQNAVVNIYSMSNGSQSSSTLYQNLRSNESGQVTGLVLPAPDVDYSLQPSDVRPYSQYIVEAIADGYETVVIEGTQ
HC52       MQDGFLTVSIIDATNNRPIQNAVVNIYSMSNGSQSSSTLYQNLRSNESGQVTGLVLPAPDVDYSLQPSDVRPYSQYIVEAIADGYETVVIEGTQ
PUC_90     MQDGFLTVSIIDATNNRPIQNAVVNIYSMSNGSQSSSTLYQNLRSNESGQVTGLVLPAPDVDYSLQPSDVRPYSQYIVEAIADGYETVVIEGTQ
LC5624     MQDGFLTVSIIDATNNRPIQNAVVNIYSMSNGSQSSSTLYQNLRSNESGQVTGLVLPAPDVDYSLQPSDVRPYSQYIVEAIADGYETVVIEGTQ
LK3P-030   MQDGFLTVSIIDATNNRPIQNAVVNIYSMSNGSQSSSTLYQNLRSNESGQVTGLVLPAPDVDYSLQPSDVRPYSQYIVEAIADGYETVVIEGTQ
LK3P-081   MQDGFLTVSIIDATNNRPIQNAVVNIYSMSNGSQSSSTLYQNLRSNESGQVTGLVLPAPDVDYSLQPSDVRPYSQYIVEAIADGYETVVIEGTQ
PUC_75     MQDGFLTVSIIDATNNRPIQNAVVNIYSMSNGSQSSSTLYQNLRSNESGQVTGLVLPAPDVDYSLQPSDVRPYSQYIVEAIADGYETVVIEGTQ
S9         MQDGFLTVSIIDATNNRPIQNAVVNIYSMSNGSQSSSTLYQNLRSNESGQVTGLVLPAPDVDYSLQPSDVRPYSQYIVEAIADGYETVVIEGTQ
C103       MQDGFLTVSIIDATNNRPIQNAVVNIYSMSNGSQSSSTLYQNLRSNESGQVTGLVLPAPDVDYSLQPSDVRPYSQYIVEAIADGYETVVIEGTQ
M68        MQDGFLTVSIIDATNNRPIQNAVVNIYSMSNGSQSSSTLYQNLRSNESGQVTGLVLPAPDVDYSLQPSDVRPYSQYIVEAIADGYETVVIEGTQ
PUC_606    MQDGFLTVSIIDATNNRPIQNAVVNIYSMSNGSQSSSTLYQNLRSNESGQVTGLVLPAPDVDYSLQPSDVRPYSQYIVEAIADGYETVVIEGTQ
ICC5       MQDGFLTVSIIDATNNRPIQNAVVNIYSMSNGSQSSSTLYQNLRSNESGQVTGLVLPAPDVDYSLQPSDVRPYSQYIVEAIADGYETVVIEGTQ
M120       MQDGFLTVSIIDATNNRPIQNAVVNIYSMSNGSQSSSTLYQNLRSNESGQVTGLVLPAPDVDYSLQPSDVRPYSQYIVEAIADGYETVVIEGTQ
P8         MQDGFLTVSIIDATNNRPIQNAVVNIYSMSNGSQSSSTLYQNLRSNESGQVTGLVLPAPDVDYSLQPSDVRPYSQYIVEAIADGYETVVIEGTQ
P12        MQDGFLTVSIIDATNNRPIQNAVVNIYSMSNGSQSSSTLYQNLRSNESGQVTGLVLPAPDVDYSLQPSDVRPYSQYIVEAIADGYETVVIEGTQ
```

```
                100       110       120       130       140       150       160       170       180
R20291     LLATIVARQGVPMSPRTRSKRSFSRQSELIFDIGEHTLYGTYPPKIPESNLKPLPPPTGFVVLDNPVVPEFIVVHDGLPEDSSAPNYWIPFKEY

PUC_256    LLATIEARQGVPMSPRTRSKRSFSRQSELIFDIGEHTLYGTYPPKIPESNLKPLPPPTGFVVLDNPVVPEFIVVHDGLPEDSSAPNYWIPFKEY
HC52       LLATIEARQGVPMSPRTRSKRSFSRQSELIFDIGEHTLYGTYPPKIPESNLKPLPPPTGFVVLDNPVVPEFIVVHDGLPEDSSAPNYWIPFKEY
PUC_90     LLATIEARQGVPMSPRTRSKRSFSRQSELIFDIGEHTLYGTYPPKIPESNLKPLPPPTGFVVLDNPVVPEFIVVHDGLPEDSSAPNYWIPFKEY
LC5624     LLATIEARQGVPMSPRTRSKRSFSRQSELIFDIGEHTLYGTYPPKIPESNLKPLPPPTGFVVLDNPVVPEFIVVHDGLPEDSSAPNYWIPFKEY
LK3P-030   LLATIEARQGVPMSPRTRSKRSFSRQSELIFDIGEHTLYGTYPPKIPESNLKPLPPPTGFVVLDNPVVPEFIVVHDGLPEDSSAPNYWIPFKEY
LK3P-081   LLATIEARQGVPMSPRTRSKRSFSRQSELIFDIGEHTLYGTYPPKIPESNLKPLPPPTGFVVLDNPVVPEFIVVHDGLPEDSSAPNYWIPFKEY
PUC_75     LLATIEARQGVPMSPRTRSKRSFSRQSELIFDIGEHTLYGTYPPKIPESNLKPLPPPTGFVVLDNPVVPEFIVVHDGLPEDSSAPNYWIPFKEY
S9         LLATIEARQGVPMSPRTRSKRSFSRQSELIFDIGEHTLYGTYPPKIPESNLKPLPPPTGFVVLDNPVVPEFIVVHDGLPEDSSAPNYWIPFKEY
C103       LLATIEARQGVPMSPRTRSKRSFSRQSELIFDIGEHTLYGTYPPKIPESNLKPLPPPTGFVVLDNPVVPEFIVVHDGLPEDSSAPNYWIPFKEY
M68        LLATIEARQGVPMSPRTRSKRSFSRQSELIFDIGEHTLYGTYPPKIPESNLKPLPPPTGFVVLDNPVVPEFIVVHDGLPEDSSAPNYWIPFKEY
PUC_606    LLATIEARQGVPMSPRTRSKRSFSRQSELIFDIGEHTLYGTYPPKIPESNLKPLPPPTGFVVLDNPVVPEFIVVHDGLPEDSSAPNYWIPFKEY
ICC5       LLATIEARQGVPMSPRTRSKRSFSRQSELIFDIGEHTLYGTYPPKIPESNLKPLPPPTGFVVLDNPVVPEFIVVHDGLPEDSSAPNYWIPFKEY
M120       LLATIEARQGVPMSPRTRSKRSFSRQSELIFDIGEHTLYGTYPPKIPESNLKPLPPPTGFVVLDNPVVPEFIVVHDGLPEDSSAPNYWIPFKEY
P8         LLATIEARQGVPMSPRTRSKRSFSRQSELIFDIGEHTLYGTYPPKIPESNLKPLPPPTGFVVLDNPVVPEFIVVHDGLPEDSSAPNYWIPFKEY
P12        LLATIEARQGVPMSPRTRSKRSFSRQSELIFDIGEHTLYGTYPPKIPESNLKPLPPPTGFVVLDNPVVPEFIVVHDGLPEDSSAPNYWIPFKEY
```

```
            190       200       210       220       230       240       250       260       270       280
R20291     IKNIASSEIYSTWPEQTIYANVIAIISFTLNRVFTEWYRNKGYNFTITSTTAYDHKFINNRNLFEPINVVVDAIFNTFIKRPPTSRQPLLAQYC

PUC_256    IKNIASSEIYSTWPEQTIYANVIAIISFTLNRVFTEWYRNKGYNFTITSTTAYDHKFINNRNLFEPINVVVDAIFNTFIKRPPTSRQPLLAQYC
HC52       IKNIASSEIYSTWPEQTIYANVIAIISFTLNRVFTEWYRNKGYNFTITSTTAYDHKFINNRNLFEPINVVVDAIFNTFIKRPPTSRQPLLAQYC
PUC_90     IKNIASSEIYSTWPEQTIYANVIAIISFTLNRVFTEWYRNKGYNFTITSTTAYDHKFINNRNLFEPINVVVDAIFNTFIKRPPTSRQPLLAQYC
LC5624     IKNIASSEIYSTWPEQTIYANVIAIISFTLNRVFTEWYRNKGYNFTITSTTAYDHKFINNRNLFEPINVVVDAIFNTFIKRPPTSRQPLLAQYC
LK3P-030   IKNIASSEIYSTWPEQTIYANVIAIISFTLNRVFTEWYRNKGYNFTITSTTAYDHKFINNRNLFEPINVVVDAIFNTFIKRPPTSRQPLLAQYC
LK3P-081   IKNIASSEIYSTWPEQTIYANVIAIISFTLNRVFTEWYRNKGYNFTITSTTAYDHKFINNRNLFEPINVVVDAIFNTFIKRPPTSRQPLLAQYC
PUC_75     IKNIASSEIYSTWPEQTIYANVIAIISFTLNRVFTEWYRNKGYNFTITSTTSYDHKFINNRNLFEPINVVVDAIFNTFIKRPPTSRQPLLAQYC
S9         IKNIASSEIYSTWPEQTIYANVIAIISFTLNRVFTEWYRNKGYNFTITSTTSYDHKFINNRNLFEPINVVVDAIFNTFIKRPPTSRQPLLAQYC
C103       IKNIASSEIYSTWPEQTIYANVIAIISFTLNRVFTEWYRNKGYNFTITSTTSYDHKFINNRNLFEPINVVVDAIFNTFIKRPPTSRQPLLAQYC
M68        IKNIASSEIYSTWPEQTIYANVIAIISFTLNRVFTEWYRNKGYNFTITSTTAYDHKFINNRNLFEPINVVVDAIFNTFIKRPPTSRQPLLAQYC
PUC_606    IKNIASSEIYSTWPEQTIYANVIAIISFTLNRVFTEWYRNKGYNFTITSTTAYDHKFINNRNLFEPINVVVDAIFNTFIKRPPTSRQPLLAQYC
ICC5       IKNIASSEIYSTWPEQTIYANVIAIISFTLNRVFTEWYRNKGYNFTITSTTAYDHKFINNRNLFEPINVVVDAIFNTFIKRPPTSRQPLLAQYC
M120       IKNIASSEIYSTWPEQTIYANVIAIISFTLNRVFTEWYRNKGYNFTITSTTTYDHKFINNRNLFEPINVVVDAIFNTFIKRPPTSRQPLLAQYC
P8         IKNIASSEIYSTWPEQTIYANVIAIISFTLNRVFTEWYRNKGYNFTITSTTTYDHKFINNRNLFEPINVVVDAIFNTFIKRPPTSRQPLLAQYC
P12        IKNIASSEIYSTWPEQTIYANVIAIISFTLNRVFTEWYRNKGYNFTITSTTTYDHKFINNRNLFEPINVVVDAIFNTFIKRPPTSRQPLLAQYC
```

```
                  290       300       310       320       330       340       350       360       370
R20291     DGQKSQCPDQMTQWGSKDLGDQGYDYESILRYFYGDEIVFERAPIVSGVPVSFPGTTLQVGSSGQYVRTIQNQLNAISNSYPAVPKVIEDGIYG

PUC_256    DGQKSQCPDQMTQWGSKDLGDQGYDYESILRYFYGDEIVFERAPIVSGVPVSFPGTTLQVGSSGQYVRTIQNQLNAISNSYPAVPKVIEDGIYG
HC52       DGQKSQCPDQMTQWGSKDLGDQGYDYESILRYFYGDEIVFERAPIVSGVPVSFPGTTLQVGSSGQYVRTIQNQLNAISNSYPAVPKVIEDGIYG
PUC_90     DGQKSQCPDQMTQWGSKDLGDQGYDYESILRYFYGDEIVFERAPIVSGVPVSFPGTTLQVGSSGQYVRTIQNQLNAISNSYPAVPKVIEDGIYG
LC5624     DGQKSQCPDQMTQWGSKDLGDQGYDYESILRYFYGDEIVFERAPIVSGVPVSFPGTTLQVGSSGQYVRTIQNQLNAISNSYPAVPKVIEDGIYG
LK3P-030   DGQKSQCPDQMTQWGSKDLGDQGYDYESILRYFYGDEIVFERAPIVSGVPVSFPGTTLQVGSSGQYVRTIQNQLNAISNSYPAVPKVIEDGIYG
LK3P-081   DGQKSQCPDQMTQWGSKDLGDQGYDYESILRYFYGDEIVFERAPIVSGVPVSFPGTTLQVGSSGQYVRTIQNQLNAISNSYPAVPKVIEDGIYG
PUC_75     DGQKSQCPDQMTQWGSKDLGDQGYDYESILRYFYGDEIVFERAPIVSGVPVSFPGTTLQVGSSGQYVRTIQNQLNAISNSYPAVPKVIEDGIYG
S9         DGQKSQCPDQMTQWGSKDLGDQGYDYESILRYFYGDEIVFERAPIVSGVPVSFPGTTLQVGSSGQYVRTIQNQLNAISNSYPAVPKVIEDGIYG
C103       DGQKSQCPDQMTQWGSKDLGDQGYDYESILRYFYGDEIVFERAPIVSGVPVSFPGTTLQVGSSGQYVRTIQNQLNAISNSYPAVPKVIEDGIYG
M68        DGQKSQCPDQMTQWGSKDLGDQGYDYESILRYFYGDEIVFERAPIVSGVPVSFPGTTLQVGSSGQYVRTIQNQLNAISNSYPAVPKVIEDGIYG
PUC_606    DGQKSQCPDQMTQWGSKDLGDQGYDYESILRYFYGDEIVFERAPIVSGVPVSFPGTTLQVGSSGQYVRTIQNQLNAISNSYPAVPKVIEDGIYG
ICC5       DGQKSQCPDQMTQWGSKDLGDQGYDYESILRYFYGDEIVFERAPIVSGVPVSFPGTTLQVGSSGQYVRTIQNQLNAISNSYPAVPKVIEDGIYG
M120       DGQKSQCPDQMTQWGSKDLGDQGYDYESILRFFYGDEIVFERAPIVSGVPVSFPGTTLQVGSSGQYVRTIQNQLNAISNSYPAVPKVIEDGIYG
P8         DGQKSQCPDQMTQWGSKDLGDQGYDYESILRFFYGDEIVFERAPIVSGVPVSFPGTTLQVGSSGQYVRTIQNQLNAISNSYPAVPKVIEDGIYG
P12        DGQKSQCPDQMTQWGSKDLGDQGYDYESILRFFYGDEIVFERAPIVSGVPVSFPGTTLQVGSSGQYVRTIQNQLNAISNSYPAVPKVIEDGIYG
```

```
              380       390       400       410         423
R20291     ADTENAVKIFQGIFGLPQSGVVDFKTWYEISRVYVATTRIASLNPLI

PUC_256    TDTENAVKIFQGIFGLPQSGVVDFKTWYEISRVYVATTRIASLNPLI
HC52       TDTENAVKIFQGIFGLPQSGVVDFKTWYEISRVYVATTRIASLNPLI
PUC_90     TDTENAVKIFQGIFGLPQSGVVDFKTWYEISRVYVATTRIASLNPLI
LC5624     TDTENAVKIFQGIFGLPQSGVVDFKTWYEISRVYVATTRIASLNPLI
LK3P-030   TDTENAVKIFQGIFGLPQSGVVDFKTWYEISRVYVATTRIASLNPLI
LK3P-081   TDTENAVKIFQGIFGLPQSGVVDFKTWYEISRVYVATTRIASLNPLI
PUC_75     TDTENAVKIFQGIFGLPQSGVVDFKTWYEISRVYVATTRIASLNPLI
S9         TDTENAVKIFQGIFGLPQSGVVDFKTWYEISRVYVATTRIASLNPLI
C103       TDTENAVKIFQGIFGLPQSGVVDFKTWYEISRVYVATTRIASLNPLI
M68        TDTENAVKIFQGIFGLPQSGVVDFKTWYEISRVYVATTRIASLNPLI
PUC_606    TDTENAVKIFQGIFGLPQSGVVDFKTWYEISRVYVATTRIASLNPLI
ICC5       TDTENAVKIFQGIFGLPQSGVVDFKTWYEISRVYVATTRIASLNPLI
M120       TDTENAVRIFQGIFGLPQSGVVDFKTWYEISRVYVATTRIASLNPLI
P8         TDTENAVRIFQGIFGLPQSGVVDFKTWYEISRVYVATTRIASLNPLI
P12        TDTENAVRIFQGIFGLPQSGVVDFKTWYEISRVYVATTRIASLNPLI
```

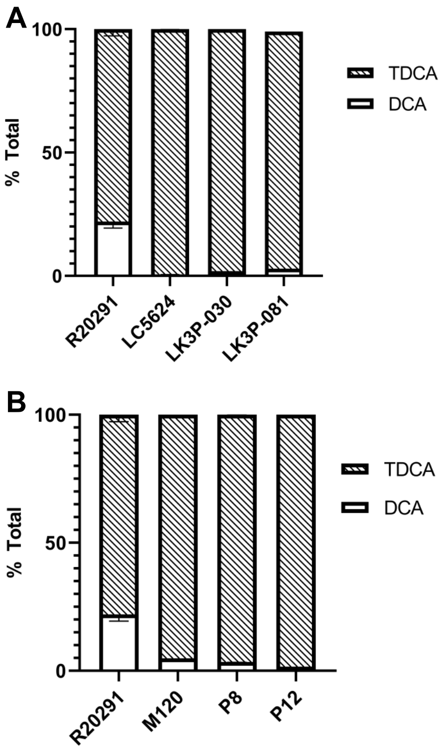
A
100
50
0
% Total
TDCA
DCA
R20291
LC5624
LK3P-030
LK3P-081
B
100
50
0
% Total
TDCA
DCA
R20291
M120
P8
P12

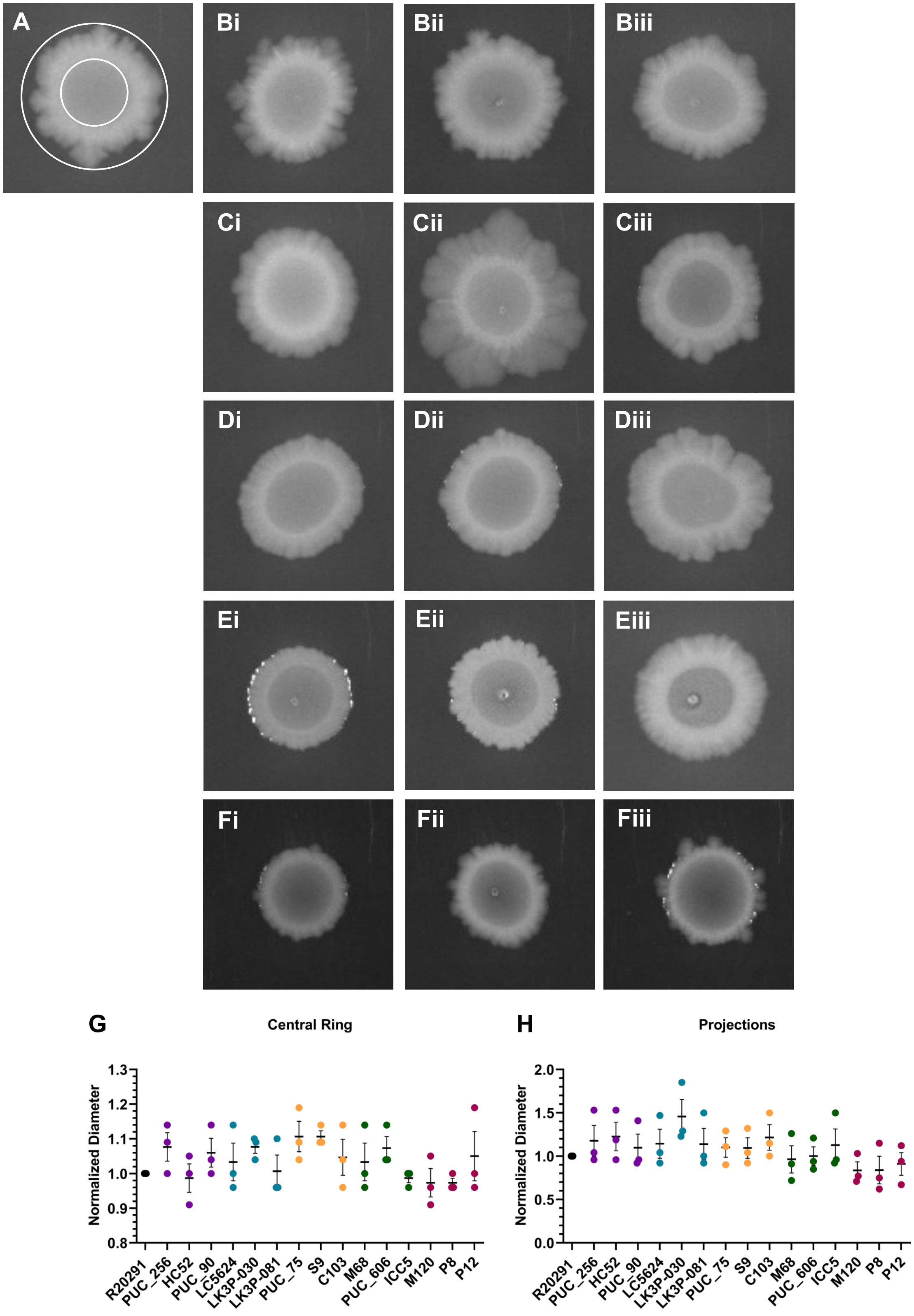
A
Bi
Bii
Biii
Ci
Cii
Ciii
Di
Dii
Diii
Ei
Eii
Eiii
Fi
Fii
Fiii
G
Central Ring
Normalized Diameter
1.3
1.2
1.1
1.0
0.9
0.8
R20291
PUC_256
HC52
PUC_90
LC5624
LK3P-030
LK3P-081
PUC_75
S9
C103
M68
PUC_606
ICC5
M120
P8
P12
H
Projections
Normalized Diameter
2.0
1.5
1.0
0.5
0.0
R20291
PUC_256
HC52
PUC_90
LC5624
LK3P-030
LK3P-081
PUC_75
S9
C103
M68
PUC_606
ICC5
M120
P8
P12
