# Supplementary material for: Phenotypic analysis of various Clostridioides difficile ribotypes reveals consistency among core processes
Source: Appl Environ Microbiol. 2025 Jun 24;91(7):e00964-25. doi: 10.1128/aem.00964-25 (PMC12285255; doi:10.1128/aem.00964-25)
Supplement: Table S1 and legends — Legends for Fig. S1 to S15; Table S1, strains, primers, and plasmids used in ELISA. [file aem.00964-25-s0003.docx]

**Supplemental Material**

**Figure S1: *C. difficile* R20291 controls and EC_50,glycine_ assay**

A) Germination of purified *C. difficile* R20291 spores under control conditions; buffer only, TA only, Gly + DMSO, and TA + Gly + DMSO. These controls were run on every germination assay plate. B) Germination of purified *C. difficile* R20291 spores exposed to the indicated concentrations of glycine. C) Michaelis-Menten plot generated using the greatest slopes determined from the germination curves shown in panel B. D) Lineweaver-Burke plot generated by taking the reciprocal of at least 4 data points form panel C. The slope of this plot is the EC_50_ value reported in the main text.

**Figure S2: *C. difficile* R20291 BSHA controls**

Chromatograms for R20291 without bile salt treatment (A), with TA, HDCA, and CA added after centrifugation (B), and with TA added prior to the 24 hour incubation (C). The chromatograms from A-C are overlayed in (D) to confirm the identity of the peaks.

**Figure S3: Additional phylogenies**

The neighbor joining phylogeny generated for the strains in this study derived from LCBs 115, a 1,078,944 bp segment (A) and 95, a 251,467 bp segment extracted from the MAUVE alignment (B). The phylogeny was constructed using the Geneious Tree Builder application in Geneious Prime software using the Tamura-Nei genetic distance model. Strains are grouped by their respective ribotypes / clades with the scale bar representing the number of substitutions per 1000 bp.

**Figure S4: Organization of phenotypically relevant genes**

Operon organization of catabolite control protein *ccpA* (A)*,* the xylose metabolism genes *xylR, xylB,* and *xylA* (B), the trehalose metabolism genes *treR* and *treA* (C), and the germination proteins *cspBA, cspC* (D) and *SleC* (E). This schematic was generated using BioRender for the genes encoding the protein sequences aligned in the following supplemental figures.

**Figure S5: ClustalOmega alignment of CcpA**

The gene sequence of *ccpA* was extracted from the MAUVE alignment. The sequences were translated and aligned using ClustalOmega. Similarity to the *C. difficile* R20291 control is indicated by shading with white indicating 100% similarity and black indicating <60 % identity.

**Figure S6: ClustalOmega alignment of XylA**

The gene sequence of *xylA* was extracted from the MAUVE alignment. The sequences were translated and aligned using ClustalOmega. Similarity to the *C. difficile* R20291 control is indicated by shading with white indicating 100% similarity and black indicating <60 % identity.

**Figure S7: ClustalOmega alignment of XylB**

The gene sequence of *xylB* was extracted from the MAUVE alignment. The sequences were translated and aligned using ClustalOmega. Similarity to the *C. difficile* R20291 control is indicated by shading with white indicating 100% similarity and black indicating <60 % identity.

**Figure S8: ClustalOmega alignment of XylR**

The gene sequence of *xylR* was extracted from the MAUVE alignment. The sequences were translated and aligned using ClustalOmega. Similarity to the *C. difficile* R20291 control is indicated by shading with white indicating 100% similarity and black indicating <60 % identity.

**Figure S9: ClustalOmega alignment of TreA**

The gene sequence of *treA* was extracted from the MAUVE alignment. The sequences were translated and aligned using ClustalOmega. Similarity to the *C. difficile* R20291 control is indicated by shading with white indicating 100% similarity and black indicating <60 % identity.

**Figure S10: ClustalOmega alignment of TreR**

The gene sequence of *treR* was extracted from the MAUVE alignment. The sequences were translated and aligned using ClustalOmega. Similarity to the *C. difficile* R20291 control is indicated by shading with white indicating 100% similarity and black indicating <60 % identity.

**Figure S11: ClustalOmega alignment of CspBA**

The gene sequence of *cspBA* was extracted from the MAUVE alignment. The sequences were translated and aligned using ClustalOmega. Similarity to the *C. difficile* R20291 control is indicated by shading with white indicating 100% similarity and black indicating <60 % identity.

**Figure S12: ClustalOmega alignment of CspC**

The gene sequence of *cspC* was extracted from the MAUVE alignment. The sequences were translated and aligned using ClustalOmega. Similarity to the *C. difficile* R20291 control is indicated by shading with white indicating 100% similarity and black indicating <60 % identity.

**Figure S13: ClustalOmega alignment of SleC**

The gene sequence of *sleC* was extracted from the MAUVE alignment. The sequences were translated and aligned using ClustalOmega. Similarity to the *C. difficile* R20291 control is indicated by shading with white indicating 100% similarity and black indicating <60 % identity.

**Figure S14: Bile salt hydrolase activity - TDCA**

Each strain was grown in the presence of 1 mM TDCA and incubated for 24 hours. The bile salts present in each culture following incubation were identified / quantified by reverse-phase high performance liquid chromatography (HPLC). Percent deconjugation was calculated using the following formula: % deconjugation = DCA / (TDCA+DCA). Data points represent the average from independent biological triplicates with error bars representing the standard error of the mean.

**Figure S15: Surface motility assay**

Stationary phase cultures were spotted into BHIS and incubated for 5 days prior to imaging. Each panel is a representative image from three distinct biological replicates. A) *C. difficile* R20291. B) Clade 1A-RT014-020 strains: Bi) *C. difficile* PUC_256, Bii) *C. difficile* HC52, and Biii) *C. difficile* PUC_90. C) Clade 1B-RT106 strains: Ci) *C. difficile* LC5624, Cii) *C. difficile* LK3P-030, and Ciii) *C. difficile*. LK3P-081. D) Clade 3-RT023 strains: Di) *C. difficile* PUC_75, Dii) *C. difficile* S9, and Diii) *C. difficile* C103. E) Clade 4-RT017 strains: Ei) *C. difficile* M68, Eii) *C. difficile* PUC_606, and Eiii) *C. difficile* ICC5. F) Clade 5-RT078 strains: Fi) *C. difficile* M120, Fii) *C. difficile* P8, and Fiii) *C. difficile* P12. G) The diameter of the central growth ring (the smaller circle shown in panel A) was quantified for each strain and normalized to the value obtained for R20291. H) The diameter of growth including the projections (the larger circle shown in panel A) was quanitifed for each strain and normalized to the value obtained for *C. difficile* R20291. Šidák’s multiple comparisons test (comparing all strains to *C. difficile* R20291) was used. No statistically significant differences between strains were found. **Table S1: Strains, primers, and plasmids used in the ELISA assay**

| **Strain/Plasmid/Primer** | **Phenotype/Sequence** | **Source** |
| --- | --- | --- |
| MAB28 | *C. difficile* R20291 Δ*sigG*Δ*tcdR* | This study |
| pJB94 |  | (43) |
| pMAB50 |  | This study |
| pMAB55 |  | This study |
| 173: 5' catP 3 | atggtatttgaaaaaattgataaaaatag | This study |
| 174: 3' catP 2 | ttaactatttatcaattcctgcaattcg | This study |
| 2980: delsigG_mutconf_FW | gaatatcacagtcctacatatctagacttg | This study |
| 2981:  delsigG_mutconf_RV | ttaaaggagttgtttctattttctcatacg | This study |
| 2992: deltcdR_mutconf_FW | aatgattgattaagttaaaaatgtgcatg | This study |
| 2993: deltcdR_mutconf_RV | ggctttatttctaccagactttttatatg | This study |
| 3814: deltcdR_UP_FW | ggaaacagctatgaccgcggccgcaaagatactattttagtcttgaaaatatttagtttg | This study |
| 3815: deltcdR_UP_RV | tattgtaaatatttctttaaattcattaaaaaatcatcctctcttatatttataatgatg | This study |
| 3816: deltcdR_DN_FW | tataaatataagagaggatgattttttaatgaatttaaagaaatatttacaatagaaatc | This study |
| 3817: deltcdR_DN_RV | catgtctgcaggcctcgagtctgttaaactatttatatcttttaattttaaatatttttc | This study |
| 3819: pJB94_int_RV | tgctgcaaggcgattaagttg | This study |
| 4133: delsigG_LH_FW | tatcaggaaacagctatgaccgcggccgctaaaaatataaaattggcaacttatgcttcg | This study |
| 4134: delsigG_LH_RV | aaaataatgtttacagtccatggattacatattttcagtcctctctcaaaagtattcatc | This study |
| 4135: delsigG_RH_FW | tttgagagaggactgaaaatatgtaatccatggactgtaaacattattttaatttaaatg | This study |
| 4136: delsigG_RH_RV | agtgccaagcttgcatgtctgcaggcctcgagtaaacctctttttacatgattcacactc | This study |
